# Stepwise N–H bond formation from N_2_-derived iron nitride, imide and amide intermediates to ammonia†
†Electronic supplementary information (ESI) available: Synthetic, spectroscopic, and structural details; independent syntheses of some compounds. CCDC 1450155–1450161. For ESI and crystallographic data in CIF or other electronic format see DOI: 10.1039/c6sc00423g


**DOI:** 10.1039/c6sc00423g

**Published:** 2016-06-14

**Authors:** K. Cory MacLeod, Sean F. McWilliams, Brandon Q. Mercado, Patrick L. Holland

**Affiliations:** a Department of Chemistry , Yale University , 225 Prospect Street , New Haven , Connecticut 06520 , USA . Email: patrick.holland@yale.edu

## Abstract

Reduction of N_2_ to ammonia in nature and in electrocatalysis takes place through 1-proton/1-electron steps, motivating efforts to experimentally study the steps during proton/electron transfer to well-characterized N_2_-derived species with bridging nitrides. We report here the protonation and reduction reactions of an N_2_-derived iron bis(nitride) complex (Rodriguez *et al.*, *Science*, 2011, **334**, 780). We isolate and definitively characterize triiron imido and amido intermediates that lie along the path to ammonia formation, and Mössbauer spectroscopy shows the oxidation level of iron atoms in these mixed-valence clusters. The first two H atoms add to one of the two nitrides of the bis(nitride) complex, and the proton-coupled electron transfer in the second step can be concerted or stepwise depending on the sources of protons and electrons. The characterization of partially protonated nitrides and their mechanisms of formation are expected to guide efforts to convert N_2_ to ammonia with mild acids.

## Abstract 2

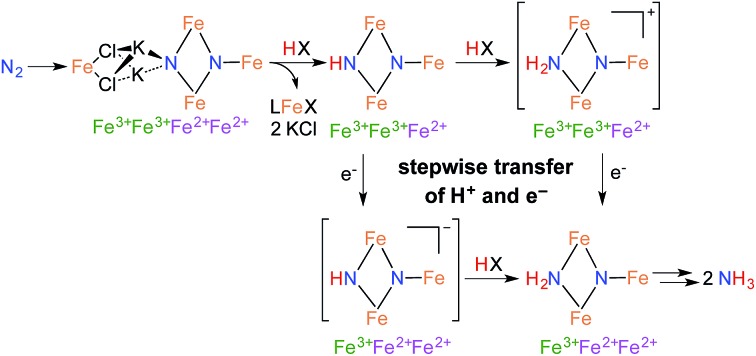
The pathway from N_2_ to NH_3_ at low-coordinate iron complexes is shown through crystallographic and spectroscopic characterization of intermediates, including bridging nitride, imide, and amides. Proton-coupled electron transfer plays a key role in the transformations.

## Introduction

The formation of ammonia from N_2_ plays a critical role in the Earth's nitrogen cycle, and supports the growth of plants that are the nutritional basis of the human population.^1^ Conversion of N_2_ into bioavailable ammonia takes place through both natural (nitrogenase enzymes) and industrial (Haber–Bosch) processes, and both systems use multi-iron active sites to achieve this transformation. The industrial Haber–Bosch process typically uses a heterogeneous iron catalyst to reduce N_2_ and H_2_ into ammonia at elevated temperatures and pressures.^2^ N_2_ is proposed to bind at surface sites on the iron catalyst followed by rate-limiting N–N bond cleavage.^3^ In nature, nitrogenase enzymes operate at ambient temperatures and pressures by reducing N_2_ with protons and electrons to form ammonia.^4^ In nitrogenases, the active sites for nitrogen fixation are large multimetallic clusters (FeMoco, FeVco, or FeFeco in different nitrogenases) containing at least seven iron atoms.^5^ In both catalytic systems, detailed kinetic studies have stimulated mechanistic ideas, but the atomic-level details of the bond-cleaving and bond-forming processes on the iron catalysts are still developing.^6^,^7^ More recent efforts have turned to electrocatalysts for N_2_ reduction using protons and electrons, which currently require substantial overpotentials and typically use high temperatures.^8^ In these cases, mechanistic information has come primarily from computations.^9^

Well-characterized homogeneous complexes derived from iron and N_2_ can give fundamental insight into potentially feasible mechanisms, because one can structurally characterize iron sites with partially reduced N_2_, and monitor elementary transformations along the way to ammonia.^10^ To this end, a growing number of molecular iron-based catalysts for N_2_ reduction are emerging.^11^,^12^ Due to the Fe nitrido species in surface catalysts,^3^ chemists have been particularly excited to study molecular iron nitride complexes in order to illuminate their ability to form ammonia, but nitride-containing molecular iron compounds are rare. Brown and Peters reported a diiron μ-nitride complex that produces NH_3_ in 80–95% yield upon treatment with 3 equiv. of HCl.^13^ With a related supporting ligand, Betley and Peters described a metastable terminal iron(iv) nitride that produces NH_3_ (41–45% yield) upon treatment with 3 equiv. of cobaltocene (Cp_2_Co) and 3 equiv. of [LutH][BPh_4_] (Lut = 2,6-lutidine).^14^ Smith and co-workers described iron(iv) and iron(v) systems of terminally-bound nitride ligands, which produced NH_3_ in yields that depended on the starting oxidation state of the Fe complex, as well as the choice of proton and electron source. For example, the iron(iv) nitride produced NH_3_ in 74% yield when treated with excess TEMPOH (TEMPOH = 1-hydroxy-2,2,6,6-tetramethylpiperidine) and was accompanied by the formation of [PhB(MesIm)_3_]Fe–TEMPO complex and TEMPO as the main by-products.^15^ Mechanistic investigations revealed that hydrogen atom transfer from TEMPOH to the nitride is likely to be the first step in the reaction. Alternatively, the iron(v) nitride produces NH_3_ in 89% yield when treated with 3 equiv. of Cp_2_Co and 15 equiv. H_2_O.^16^ The authors suggested that one-electron reduction of the iron(v) nitride is unlikely to be the first step in the reaction since the iron(iv) nitride does not react with H_2_O. These examples from the Smith group are notable for their ability to use mild reaction conditions and relatively weak proton (or hydrogen atom) sources to produce NH_3_ in high yields. No synthetic iron nitride complexes are known to react with H_2_ to give NH_3_.^17^–^19^

Despite the growing number of iron-nitrides that give NH_3_, partially protonated intermediates (NH, NH_2_) have not yet been observed in these systems. Thus, an unmet need in the literature is a system where the individual proton-electron transfer steps on the way from nitride to NH_3_ can been studied individually. Moreover, the above iron nitride complexes do not come from N_2_: they are prepared using alternative N-atom sources such as azide^13^,^15^,^16^ or Li(dbabh) (dbabh = 2,3:5,6-dibenzo-7-azabicyclo[2.2.1]hepta-2,5-diene).^14^ Clearly, nitrides derived from N_2_ would be highly relevant to the overall N_2_ reduction catalysis.

We have reported β-diketiminate-supported Fe systems that cleave N_2_ to form the well-characterized tetrairon bis(nitride) complex **1** or close analogues.^20^ Compound **1** in turn reacts with excess HCl to give NH_4_^+^ in 82 ± 4% yield.20*a* Murray and co-workers recently reported a related triiron system that reduces N_2_ to form a triiron (NH_*x*_)_3_ cluster, in which the source of exogenous H atoms is unknown.^21^ Treatment of the triiron cluster with HCl yields NH_3_ (30 ± 2%) and independent synthesis of the analogous triiron tris(amide) cluster also produces NH_3_ in 30% yield. Together, these studies show that cooperation of three or more β-diketiminate-supported iron(i) sites can reduce N_2_ to nitrides,10e,20b which in turn can lead to ammonia. This is relevant to the Haber–Bosch process, in which surface nitride intermediates are well-established,^3^ and to potential electrocatalytic mechanisms for N_2_ reduction.

Here, we report the mechanism through which nitride is converted to ammonia within the β-diketiminate-supported Fe system, which is particularly relevant because the reactive nitrides are derived from N_2_. A range of proton sources is tested, which shows the scope of ammonia formation in this system. A major focus is the structural characterization of a series of iron nitride, imide, and amide intermediates that come from N–H bond formation.

## Results

### Optimizing ammonia yields from the tetrairon bis(nitride) complex **1** with strong acids

In the initial report, treatment of **1** with a large excess of HCl produced NH_4_^+^ in 82 ± 4% yield.20*a*^,^‡
‡NH_4_^+^ is used to describe ammonia formation throughout, except when NH_3_ is the product. We now report the results from protonating with a range of acids under optimized conditions. The yields are highly dependent on the acid chosen (Table 1). Treatment of a THF solution of **1** with H_2_SO_4_ (12 equiv.) at –96 °C provides the highest yield of NH_4_^+^ (ave. 93%) among the strong acids. We repeated the protonation with H_2_SO_4_ starting from a doubly ^15^N-labeled sample of **1**, and verified that all of the NH_4_^+^ was ^15^N labeled (Fig. S-28 and S-29†), as observed with HCl previously.20*a* Protonation of nitrides in the tetrairon complex **1** with strong acids like these also results in loss of the β-diketiminate ligands. The free β-diketimine is also observed in the ^1^H NMR spectra of the products from the reactions of **1** with benzoic acid, [LutH]Cl, and [pyH]Cl (py = pyridine).

**Table 1:** Influence of acid choice on ammonia formation

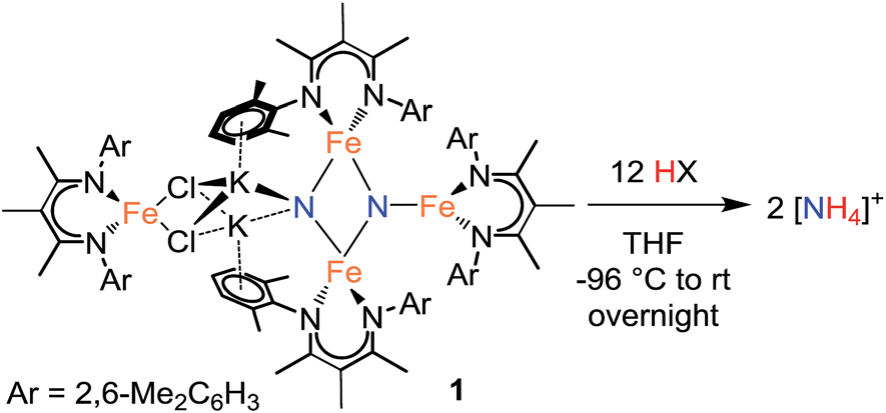			
Entry	Acid	p*K*_a_ in THF	Yield^*h*^
1	H_2_SO_4_		92%, 94%
2	[NBu_4_]HSO_4_^*a*^		60%
3	HCl_(aq)_	1.8^*c*^	91%
4	H_3_PO_4_		85%, 88%
5	HNO_3_		47%, 86%
6	[pyH]Cl^*a*^	8.2^*d*^	7%
7	[LutH]Cl^*a*^	9.5^*d*^	39%
8	[LutH]BAr^F^_4_	9.5^*d*^	43%
9	HOTs·H_2_O^*b*^	13.5^*e*^	76%, 81%
10	CF_3_CO_2_H	14.7^*f*^	75%, 79%
11	C_6_H_5_CO_2_H	19.5^*f*^	9%, 16%
12	^*t*^Bu_3_C_6_H_2_OH	27.8^*g*^	63%^*i*^ ^,^^*j*^
13	H_2_O	31.2^*c*^	96%^*j*^

^*a*^Acid was only partially soluble under reaction conditions.

^*b*^HOTs = CH_3_C_6_H_4_SO_3_H.

^*c*^p*K*_a_ in DMSO, ref. 22.

^*d*^ Ref. 23.

^*e*^Calculated value for MeSO_3_H, ref. 24.

^*f*^Calculated value, ref. 24.

^*g*^Calculated value for C_6_H_5_OH, ref. 24.

^*h*^Yields determined by the indophenol method in ref. 25, where 100% corresponds to 2 equiv. of [NH_4_]^+^ per molecule of **1**. No detectable amounts of N_2_H_4_ were formed.

^*i*^6 equiv. of ^*t*^Bu_3_C_6_H_2_OH used.

^*j*^Yield of NH_3_.

Though very strong acids generally give high yields of NH_4_^+^, we observe no simple relationship between p*K*_a_ and NH_4_^+^ yield.§
§Only one example (benzoic acid) shows an increase in ammonia yield upon subsequent treatment with excess H_2_SO_4_. See Table S-1.†
 In order to understand the importance of solubility (note that many of the reactions are multiphasic), we compared the highly soluble [LutH]BAr^F^_4_ (BAr^F^_4_ = B[C_6_H_3_{CF_3_}_2_]_4_) and the sparingly soluble [LutH]Cl, but the yields were similar (Table 1, entries 7 and 8). Table 1 shows that some conjugate bases that coordinate strongly to iron lead to a drastic decrease in yield (see entries for benzoic acid and pyridinium). This influence is shown most clearly by the difference between the yield from pyridinium chloride and 2,6-lutidinium chloride (Table 1, entries 6 and 7), which have similar p*K*_a_ values but different ability to coordinate.

We considered the possibility that the low yields are due to the formation of N_2_ from the nitrides, because pyridine has been shown previously to induce rapid N_2_ loss from **1**.^26^ Treatment of **1** with [pyH]Cl, [LutH]Cl, or HOTs (Table 1, entries 6, 7 and 9) followed by the addition of excess H_2_SO_4_ does not lead to a significant increase in the yield of NH_4_^+^ (Table S-1†), suggesting that the N atoms of the nitrides are no longer present in an activated form after addition of these weak acids.

We also examined the reaction of **1** with different amounts of H_2_SO_4_ (Table 2). Addition of less than 6 equiv. results in a decrease in ammonia yield. The need for 12 equiv. of acidic protons (6 equiv. H_2_SO_4_) is consistent with the observation that the β-diketiminate ligands are protonated in the reaction, which account for 4H^+^, and the other 8H^+^ protonate the nitride ligands to form 2 equiv. of NH_4_^+^.

**Table 2:** Dependence of ammonia yield on amount of acid

Equiv. H_2_SO_4_	[NH_4_]^+^ yield^*a*^
12	92%, 94%
11	87%
9	88%
6	95%
5	72%
4	74%
3	74%

^*a*^Yields determined by the indophenol method in ref. 25. No detectable amounts of hydrazine were formed.

**Fig. 1:**
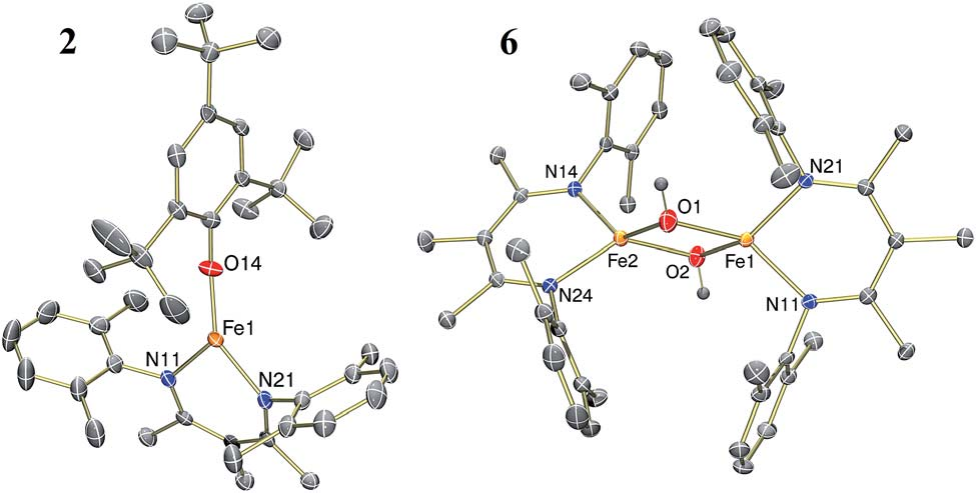
Thermal-ellipsoid plots of the molecular structures of LFe(OC_6_H_2_^*t*^Bu_3_) (**2**, left) and [LFe(μ-OH)]_2_ (**6**, right) using 50% thermal ellipsoids.

The results in Table 1 also show that the yield is often high when using weak acids. For example, [LutH]Cl and [pyH]Cl produce significantly less NH_4_^+^ than *p*-toluenesulfonic acid and trifluoroacetic acid under the reaction conditions (Table 1, entries 6, 7, 9 and 10). Strikingly, the substrate producing the highest yield of ammonia (96%) was H_2_O (Table 1, entry 13), which also has the highest p*K*_a_ of the proton sources in Table 1. The weak acid 2,4,6-tri-*tert*-butylphenol (^*t*^Bu_3_C_6_H_2_OH) is distinctive because it does not cause loss of the β-diketiminate supporting ligand. Treatment of **1** with 6 equiv. ^*t*^Bu_3_C_6_H_2_OH produces NH_3_ in 63% yield and is accompanied by formation of the iron(ii) aryloxide complex LFe(OC_6_H_2_^*t*^Bu_3_) (**2**) in 75% yield (Fig. 1). Independent synthesis and characterization of **2** is described in the ESI.†

**Scheme 1:**
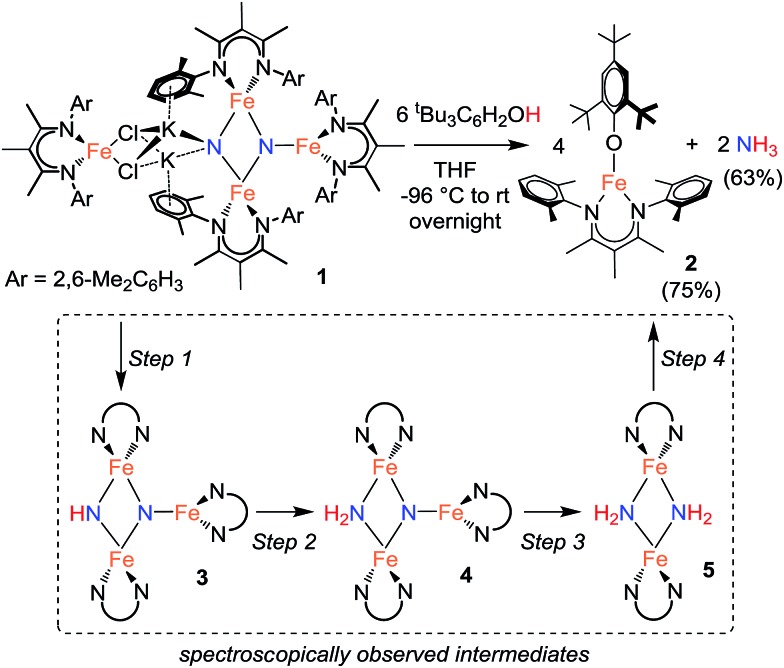
Ammonia formation from bis(nitride) **1**.

**Fig. 2:**
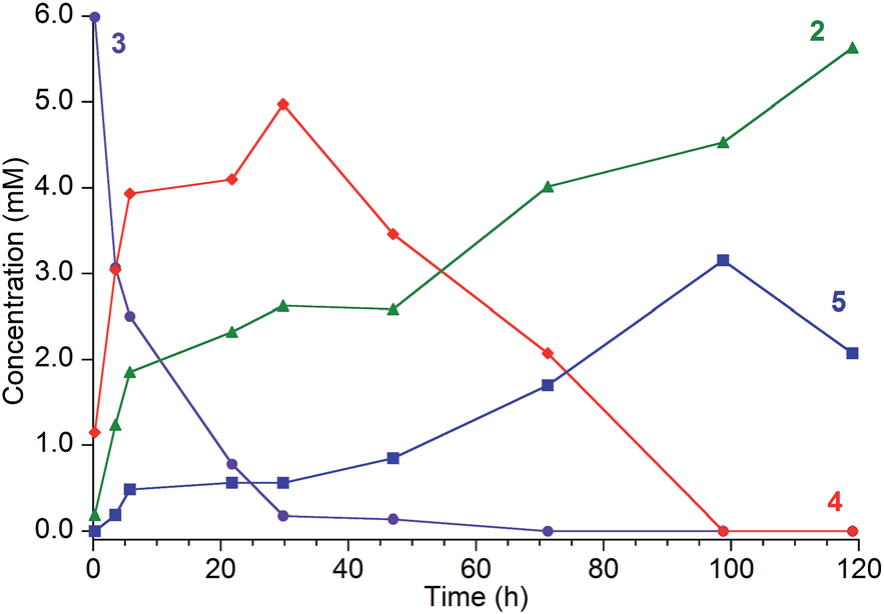
Reaction of [LFe]_2_(μ_2_-NH)(μ_3_-N)[FeL] (**3**, [black circle], [8.3 mM]) with ^*t*^Bu_3_C_6_H_2_OH [80.8 mM] in C_6_D_6_, which proceeds through intermediates [LFe]_2_(μ_2_-NH_2_)(μ_3_-N)[FeL] (**4**, ♦) and [LFe(μ-NH_2_)]_2_ (**5**, ■) to the final product LFe(OC_6_H_2_^*t*^Bu_3_) (**2**, ▲). Reaction monitored by ^1^H NMR with concentrations determined from integration of resonances relative to an internal Cp_2_Co standard. It was not possible to integrate the peaks of **2** accurately at later times because they are broadened by ammonia (see ESI†). The reactions are faster in THF or 2,5-dimethyltetrahydrofuran (see text).

^1^H NMR spectroscopy shows that Fe intermediates (**3**, **4**, and **5**) are formed during the course of the reaction between **1** and ^*t*^Bu_3_C_6_H_2_OH in C_6_D_6_ (Scheme 1), and each is described in more detail below. The first protonation to generate **3** is complete in seconds, but the subsequent steps are more amenable to analysis using ^1^H NMR spectroscopy. Reaction of **3** with excess ^*t*^Bu_3_C_6_H_2_OH in benzene-*d*_6_ shows conversion of **3** to **4**, followed by **5**, and finally **2** (Fig. 2).¶
¶The ^1^H NMR spectra suffered from peak broadening at longer time points due to the formation of ammonia, which binds reversibly to the Fe aryloxide **2** (see ESI for further details†). As a result, the concentration of **2** was not quantified beyond 119 h. Compound **1** also reacts with H_2_O under similar reaction conditions to produce the same intermediates **3**, **4**, and **5**, with the final product being the iron(ii) hydroxide dimer [LFe(μ-OH)]_2_ (**6**), as shown in Fig. 1. Although H_2_O provides higher ammonia yields compared to ^*t*^Bu_3_C_6_H_2_OH (*cf.*Table 1), the reaction with H_2_O is too rapid to monitor using NMR spectroscopy. An in-depth analysis of the individual steps that lead to ammonia formation from bis(nitride) **1** is described below, including the purification and characterization of intermediates **3**, **4**, and **5**.

### Step 1: proton transfer

Using similar reaction conditions to those described above, toluene solutions of **1** were treated with 1 equiv. of various proton sources at –78 °C (Table 3). ^1^H NMR spectra of the resulting reaction mixtures consistently contained resonances corresponding to the same predominant product (**3**), in addition to other products that varied depending on the proton source. Although the yield of **3** depended on the proton source, terminal alkynes provided the highest selectivity for this new product. In particular, reaction of **1** with 1 equiv. of the terminal alkyne PhC≡CH provided only two spectroscopically observable products, and was thus the most amenable to product isolation. The resulting reaction mixture was dried under reduced pressure and the residue was extracted with hexanes, filtered, and cooled at –40 °C to provide a mixture of single crystals with two crystal morphologies: small orange plates and large red blocks. The small orange plates were identified as a singly protonated triiron nitride/imide complex [LFe]_2_(μ_2_-NH)(μ_3_-N)[FeL] (**3**), while the large red blocks were identified as a bridging alkynyl dimer [LFe(μ-C≡CPh)]_2_ (**7a**) (independent synthesis and characterization are described in the ESI†).

**Table 3:** Acid reactions with triiron nitride/imide 2 (step 1)

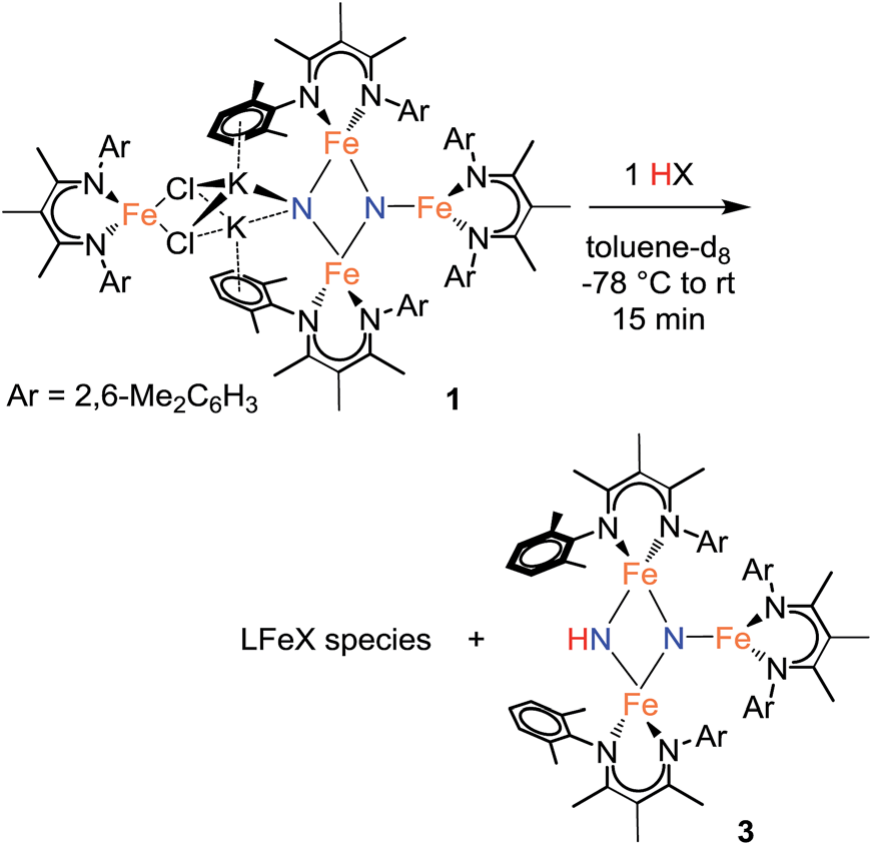				
Entry	Acid	p*K*_a_ in THF	Yield of **3**^*e*^	LFeX product^*e*^
1	C_6_H_5_C≡CH		59%	**7a** (34%)
2	(CF_3_)_2_C_6_H_2_C≡CH		99%	**7b** (ND)^*i*^
3	CH_3_(CH_2_)_5_C≡CH		0^*f*^	—
4	TEMPOH		89%^*g*^	—
5	[LutH]Cl	9.5^*a*^	63%^*g*^	[LFe(μ-Cl)]_2_ (63%)
6	[LutH]BAr^F^_4_	9.5^*a*^	16%^*g*^ ^,^^*h*^	[LFe(μ-Cl)]_2_ (12%)
7	C_6_H_5_CO_2_H	19.5^*b*^	22%^*f*^	—
8	Indene	20.1^*c*^	51%^*h*^	**8** (78%)
9	^*t*^Bu_3_C_6_H_2_OH	27.8^*d*^	78%^*h*^	**2** (71%)
10	H_2_O	31.2^*c*^	41%^*g*^ ^,^^*h*^	**6** (5%)
11	MeOH	41.2^*b*^	32%^*g*^	—

^*a*^ Ref. 23.

^*b*^Calculated value, ref. 24.

^*c*^p*K*_a_ in DMSO, ref. 22.

^*d*^Calculated value for C_6_H_5_OH, ref. 24.

^*e*^Yields determined by ^1^H NMR spectroscopy.

^*f*^LFe(η^6^-toluene) product also formed.

^*g*^Unidentified by-products are also observed.

^*h*^Triiron nitride/amide **4** is also formed as a minor product.

^*i*^Yield not determined due to low solubility.

**Fig. 3:**
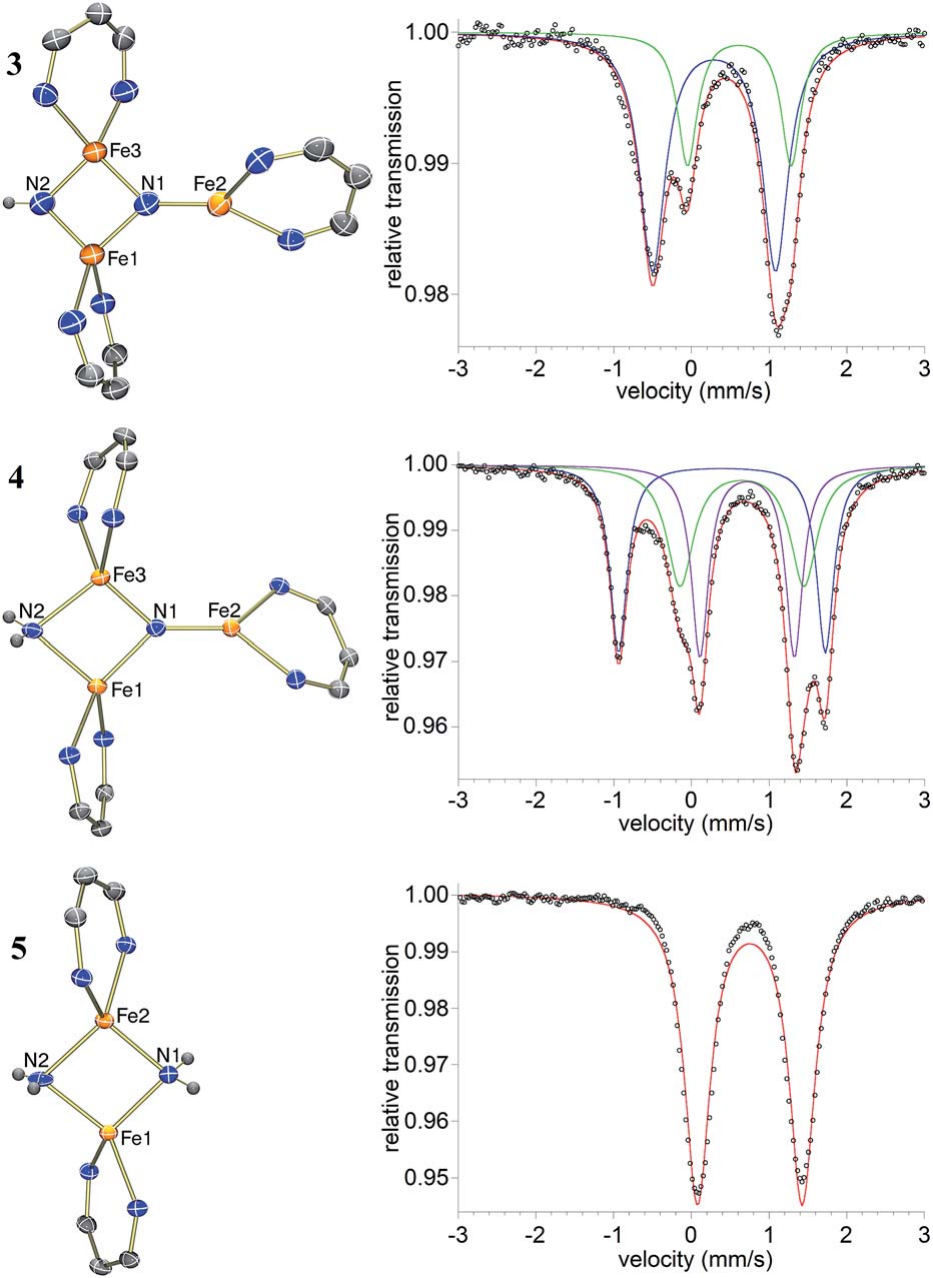
Thermal-ellipsoid plots of the molecular structures and Mössbauer spectra of solid [LFe]_2_(μ_2_-NH)(μ_3_-N)[FeL] (**3**, top), [LFe]_2_(μ_2_-NH_2_)(μ_3_-N)[FeL] (**4**, middle), and [LFe(μ-NH_2_)]_2_ (**5**, bottom) using 50% thermal ellipsoids. The methyl and 2,6-dimethylphenyl groups are omitted for clarity. In the Mössbauer spectra, the fits are indicated with colored lines and the black circles are the data.

The triiron nitride/imide complex **3** (Fig. 3) is structurally very similar to the trinuclear core of the bis(nitride) precursor **1**. There are two bridging iron atoms with Fe–N_nitride_ bond distances in the range of 1.866(5)–1.898(5) Å (compared to 1.809(2)–1.918(2) Å in **1**), as well as the three-coordinate Fe center, which has a Fe–N_nitride_ bond length of 1.836(5) Å (compared to 1.832(2) Å in **1**). Additionally, the three Fe and two N atoms of the Fe–N core are coplanar. The structural and electronic similarities between **1** and **3** are also evident by Mössbauer spectroscopy. The zero-field Mössbauer spectrum of solid **3** at 173 K shows two quadrupole doublets in a 2 : 1 ratio.‖
‖When cooled to 80 K, the Mössbauer spectrum splits to three signals, with two different iron(iii) environments. See ESI (Fig. S-2†).The larger doublet (accounting for 2/3 of the total Fe) has *δ* = 0.29 mm s^–1^ and |Δ*E*_Q_| = 1.58 mm s^–1^, while the smaller doublet (1/3 of the total Fe) has *δ* = 0.61 mm s^–1^ and |Δ*E*_Q_| = 1.34 mm s^–1^. Therefore, the Mössbauer parameters observed for **3** are consistent with an assignment of the bridging Fe centers being high-spin iron(iii), while the three-coordinate Fe center is high-spin iron(ii), analogous to the previously reported assignment of the Mössbauer spectrum for **1**.20*a* The room-temperature solution magnetic moment of 4.0(1) *μ*_B_ suggests antiferromagnetic exchange coupling between the iron centers in **3**, again analogous to **1**,20*a* although we have not yet pursued detailed magnetic studies. The ^1^H NMR spectrum of triiron nitride/imide **3** contains 12 resonances with integrations indicating a *C*_2v_ symmetric structure, as seen in the solid state. Additionally, the IR spectrum of **3** contains a weak band (3344 cm^–1^) in the N–H stretching region, providing additional support for the assignment of **3** as having a protonated N atom with a triiron nitride/imide structure.

**Scheme 2:**
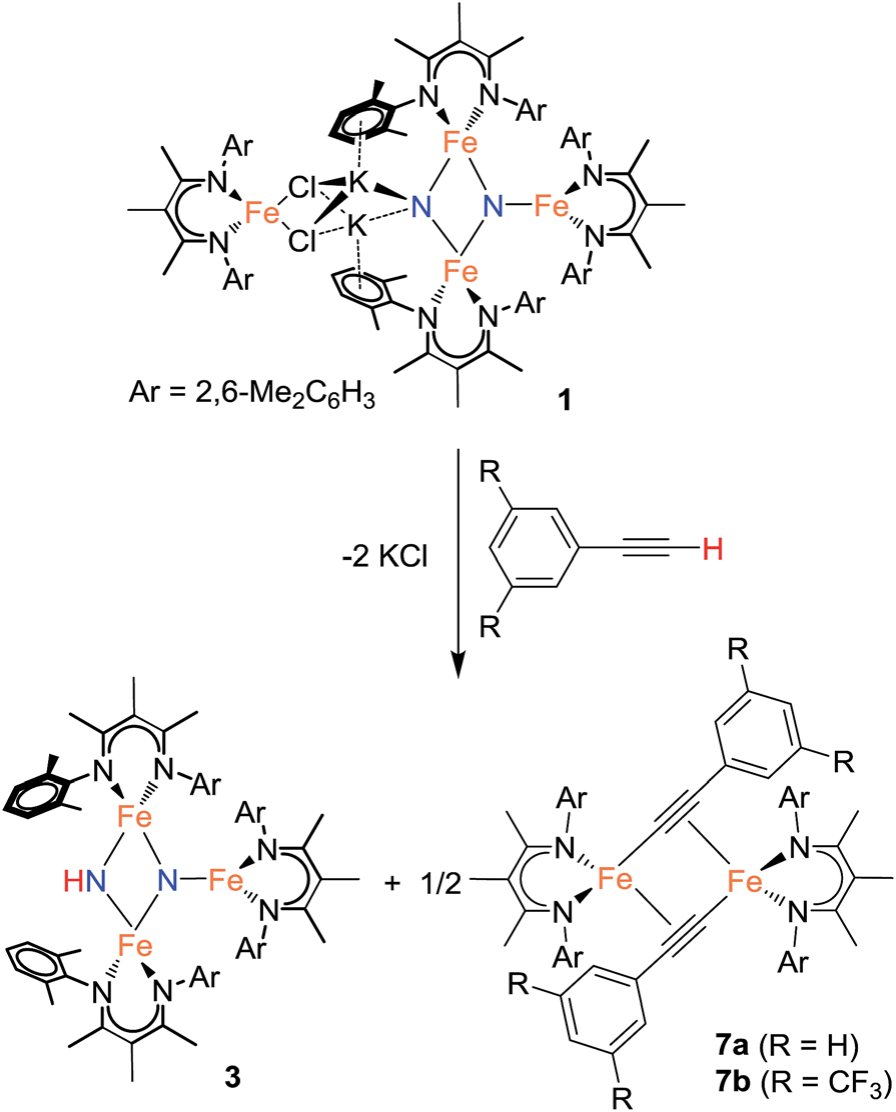
Formation of the imide/nitride **3** (step 1).

Reaction of **1** with the terminal alkyne (CF_3_)_2_C_6_H_2_C≡CH provided a much higher yield of the triiron nitride/imide **3** (99% spectroscopically, 66% isolated), as well as the complex of the conjugate base, namely [LFe{μ-C≡CC_6_H_2_(CF_3_)_2_}]_2_ (**7b**). The formation of **3** implies a redox-neutral transformation wherein the starting bis(nitride) complex **1** contains two iron(iii) and two iron(ii) ions and the resulting product **3** contains two iron(iii) and one iron(ii). The fourth iron remains as iron(ii) and gives 0.5 equiv. of the dimeric iron(ii) alkynyl product **7**, with concomitant loss of 2 equiv. of KCl (Scheme 2).

In analogous reactions, treatment of **1** with [LutH]BAr^F^_4_, PhCO_2_H, or MeOH (Table 3, entries 6, 7, and 11) produces **3** in low yields. The use of TEMPOH, [LutH]Cl, indene, and ^*t*^Bu_3_C_6_H_2_OH each produce **3** in greater than 50% yield (Table 3, entries 4, 5, 8 and 9), with the latter three substrates producing the expected by-products [LFe(μ-Cl)]_2_, LFe(η^5^-C_9_H_7_) (**8**), and LFe(OC_6_H_2_^*t*^Bu_3_) (**2**), respectively (independent synthesis and characterization of each are described in ESI†).

### Step 2: proton-coupled electron transfer to the triiron nitride/imide yields a triiron nitride/amide

With isolated samples of the singly protonated triiron nitride/imide **3**, we studied the next step along the pathway to ammonia formation. Compound **3** was less reactive toward weak acids than the bis(nitride) precursor **1**, and it showed no reaction with indene over a period of 48 h. However, **3** reacted with ^*t*^Bu_3_C_6_H_2_OH to form the triiron nitride/amide **4**, which has a 12-resonance pattern in its ^1^H NMR spectrum similar to that of **3**. The solid-state molecular structure of **4** (Fig. 3) reveals a triiron structure analogous to **3**. The main structural difference between **4** and **3** is the elongation of the Fe–N bond lengths to the μ_2_-bridging N group, with Fe–N bond lengths of 2.016(2) Å and 2.062(2) Å (compared to 1.866(5) and 1.874(5) in **3**). Additionally, two H atoms were located in the Fourier difference map, enabling assignment of **4** as a triiron nitride/amide [LFe]_2_(μ_2_-NH_2_)(μ_3_-N)[FeL] complex. The IR spectrum of **4** also contains bands at 3375 and 3299 cm^–1^, which confirm the presence of two hydrogen atoms and are attributed to symmetric and antisymmetric N–H stretching modes. The zero-field Mössbauer spectrum of solid **4** at 80 K shows three quadrupole doublets of equal area (Fig. 3), indicating that the three iron centers in **4** are inequivalent on the Mössbauer timescale (∼10^–8^ s). One doublet has *δ* = 0.65 mm s^–1^ and |Δ*E*_Q_| = 1.59 mm s^–1^, and is assigned to the three coordinate iron(ii) center by its similarity to the three coordinate iron(ii) centers in both **1** (*δ* = 0.68 mm s^–1^, |Δ*E*_Q_| = 1.54 mm s^–1^) and **3** (*δ* = 0.61 mm s^–1^, |Δ*E*_Q_| = 1.34 mm s^–1^). The two remaining doublets in the Mössbauer spectrum are therefore assigned to the two bridging Fe centers. Interestingly, one of the doublets is most consistent with a high-spin iron(iii) center with *δ* = 0.39 mm s^–1^ and |Δ*E*_Q_| = 2.66 mm s^–1^, while the other doublet has a much higher isomer shift of 0.72 mm s^–1^ (|Δ*E*_Q_| = 1.22 mm s^–1^), which is typical for a high-spin iron(ii) center. Thus, **4** is assigned as a spin-localized mixed valence cluster where one of the bridging iron centers is in the +2 oxidation state and the other is in the +3 oxidation state. This assignment of oxidation states as Fe^3+^Fe^2+^Fe^2+^ is also consistent with the single formal negative charge on an NH_2_ bridge, indicating that a formal hydrogen atom transfer occurred in the transformation of the triiron nitride/imide **3** into the triiron nitride/amide **4** (eqn (1)). Attempts to detect the presumed 2,4,6-tri-*tert*-butylphenoxyl radical (^*t*^Bu_3_C_6_H_2_O˙) product were complicated by the reaction of this radical with other species in the mixture (see ESI†).
1

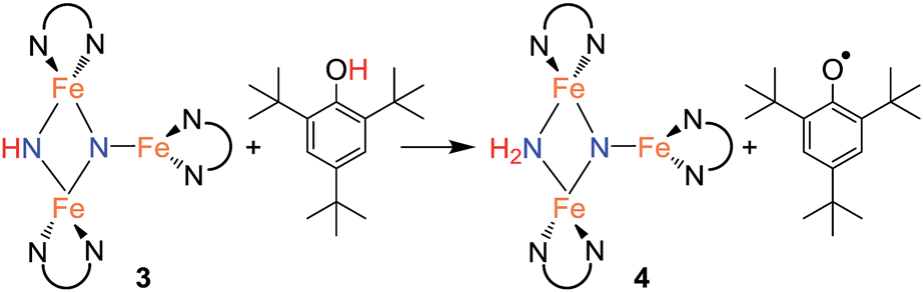




One way to distinguish between concerted and stepwise transfer of protons and electrons is by varying the solvent.^27^ The solvent choices were limited by solubility and the reactions of many common solvents with the iron compounds. We treated **3** with 10 equiv. of ^*t*^Bu_3_C_6_H_2_OH in tetrahydrofuran (*ε* = 7.5), 2,5-dimethyltetrahydrofuran (*ε* ∼ 6.5), and benzene (*ε* = 2.3).^28^ Though the presence of byproducts prevented a detailed analysis of the kinetics, the time courses of the reactions show that the half-life for conversion of **3** to **4** was roughly 15 min (THF), 100 min (2,5-Me_2_THF), or 210 min (C_6_D_6_). The differences in the rate can be attributed to differences in polarity, or to hydrogen bonding of THF with an N–H bond in the transition state. The muted differences do not definitively show whether the reaction with the substituted phenol with **3** follows a concerted or stepwise proton/electron transfer pathway.

Since cyclic voltammetry of **3** in THF shows a reversible electrochemical reduction wave at –2.28 V *versus* Cp_2_Fe^0/+^ (Fig. S-50†), we anticipated that we could evaluate the mechanism by generating a one electron reduced species through chemical reduction. First, we treated **3** with Cp*2Co (*E*_1/2_ = –1.95 V *versus* Cp_2_Fe^0/+^).^29^ Despite its less negative reduction potential, Cp*2Co reacts with **3** to give complete conversion to a mixture of **4** and the tetramethylfulvene complex (η^5^-C_5_Me_4_CH_2_)CoCp*, which is related to Cp*2Co by one proton and one electron (eqn (2)). There were no observable intermediates during the reaction. The rate of the reaction is significantly faster in THF *versus* toluene (in THF the reaction is complete in <5 min with 1 equiv. Cp*2Co *versus* 1 h in toluene). Though the ability to use a reducing agent that has a less-negative redox potential could support a concerted pathway, a stepwise pathway could still be accessible through a small equilibrium concentration of a reduced species as an intermediate (see Discussion below).

**Scheme 3:**
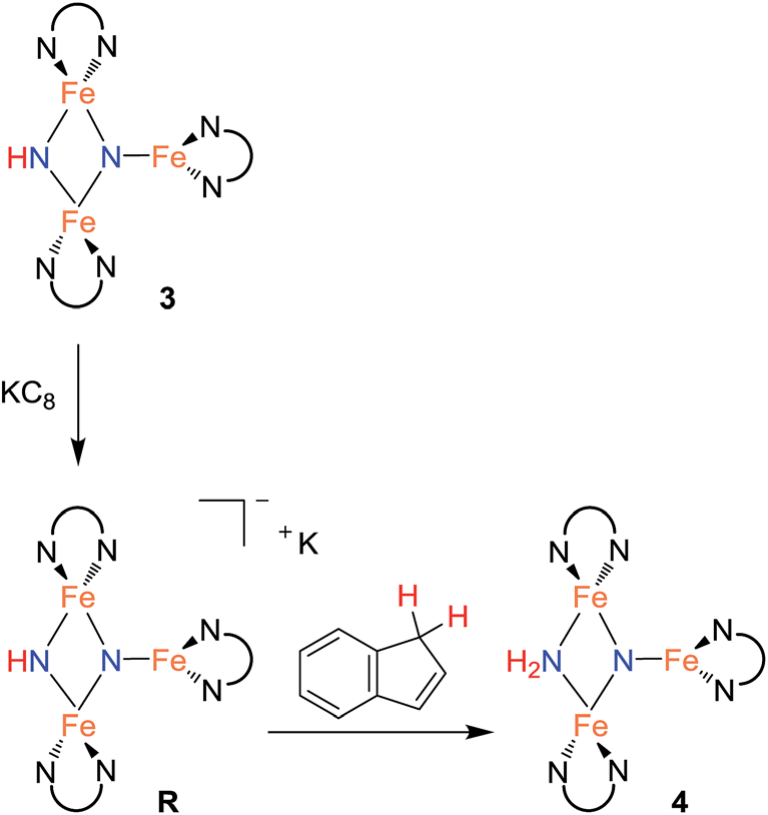
Stepwise ET/PT using separate electron and proton sources (step 2).

In an effort to observe this intermediate, compound **3** was reduced by the stronger, aprotic reductant KC_8_ at –78 °C to form a new compound **R** with a 12-resonance pattern in its ^1^H NMR spectrum. Though we were unable to isolate **R**, its zero-field Mössbauer spectrum gives insight into its nature. It shows three quadrupole doublets of equal area. One doublet has *δ* = 0.24 mm s^–1^ and |Δ*E*_Q_| = 1.94 mm s^–1^, consistent with a high-spin iron(iii) center, while the other two doublets have significantly higher isomer shifts of 0.63 mm s^–1^ (|Δ*E*_Q_| = 1.65 mm s^–1^) and 0.73 mm s^–1^ (|Δ*E*_Q_| = 1.47 mm s^–1^), consistent with high-spin iron(ii). The Mössbauer data indicate that **R** is reduced by one electron compared to the starting material **3**. Treatment of *in situ* generated **R** with 1 equiv. of the weak acid indene at room temperature results in complete conversion to **4** within 15 min (Scheme 3). **R** also reacts rapidly with [Cp*2Co]PF_6_ to form **4** and (η^5^-C_5_Me_4_CH_2_)CoCp*, indicating that **R** is a feasible intermediate in the reaction of **3** with Cp*2Co. The combination of reactivity and spectroscopic characterization of **R** is most consistent with an anionic triiron nitride/imide structure K[{LFe}_2_(μ_2_-NH)(μ_3_-N){FeL}]. More detailed characterization of **R** has been limited by our inability to isolate pure samples.
2

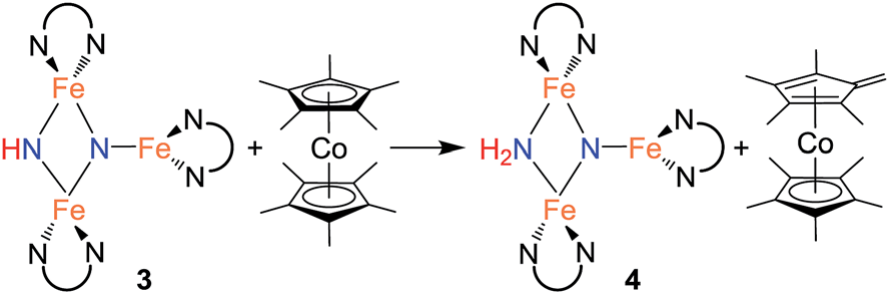




As described above, electrochemical reduction of **3** is reversible and is proposed to form the reduced intermediate **R**. To further test this hypothesis, we studied the electrochemical reduction of **3** in the presence of the weak acid indene. The peak current of the cathodic wave in the cyclic voltammogram is twice as large as the anodic wave, suggesting that the reduction now involves a two-electron process while the oxidation is a one-electron process (Fig. S-51 and S-52†). This observation suggests a mechanism in which the expected one-electron reduction of **3** to form **R** is followed by protonation (by indene) of **R** to form **4**, and finally a second one electron reduction of **4** that is reversible. This ECE mechanism is supported by the cyclic voltammetry of independently synthesized **4** in THF, which shows a reversible electrochemical reduction wave at –2.27 V *versus* Cp_2_Fe^0/+^, at the same potential as the reversible reduction of the precursor **3**. Additionally, the redox process of **4** at –2.27 V remains reversible in the presence of indene (Fig. S-55†). For both **3** and **4** the reduction is attributed to a Fe^3+/2+^ redox couple at one of the four-coordinate Fe centers, and thus it is reasonable that they occur at the same potential. Since in this reaction the electron and proton are delivered separately, it clearly shows the ability to use a stepwise PCET mechanism in this system.^30^

Overall, the ability to observe **R** and the electrochemical results show that a stepwise electron–proton transfer mechanism is possible with certain substrates. With other substrates like Cp*2Co and 2,4,6-tri-*tert*-butylphenol, we have not yet been able to distinguish between concerted and stepwise pathways.

### Step 3: protonation and reduction of the nitride/amide to give a bis(amido)diiron complex

Compound **4** also reacts with ^*t*^Bu_3_C_6_H_2_OH to form **5** and the aryloxide complex **2** (Fig. 3). Compound **5** has a N–H stretching band in its IR spectrum at 3365 cm^–1^. The ^1^H NMR spectrum of **5** contains a 5-resonance pattern suggesting a highly symmetric β-diketiminate ligand environment. X-ray diffraction analysis of a single crystal of **5** confirms the structure to be a diiron bis(amide) complex [LFe(μ-NH_2_)]_2_. The bridging N atoms in **5** have Fe–N bond distances of 2.042(2)–2.111(2) Å, which are very similar to the Fe–NH_2_ bond distances in the precursor **4** (2.016(2) Å and 2.062(2) Å). The zero-field Mössbauer spectrum of solid **5** at 80 K has a single quadrupole doublet with *δ* = 0.75 mm s^–1^ and |Δ*E*_Q_| = 1.35 mm s^–1^ (Fig. 3) indicating equivalent iron(ii) environments each having a high-spin electronic configuration. Compound **5** was independently synthesized by treating [LFe(μ-Cl)]_2_ with 2 equiv. of LiNH_2_ (see ESI†). Although the conversion of triiron nitride/amide **4** to the diiron bis(amide) **5** formally requires two proton and one electron transfer steps, no intermediates are observed in this reaction.

### Step 4: ammonia release from the diiron bis(amide) **5**

The amide ligands in **5** are readily protonated upon treatment with 2 equiv. of H_2_O to generate NH_3_ (98% yield after vacuum transfer) and a diiron bis(hydroxide) complex [LFe(μ-OH)]_2_ (**6**). Compound **6** can be synthesized independently by treating a THF solution of [LFe(μ-H)]_2_ (ref. 20*a*) with 2 equiv. of H_2_O. Compound **6** has a distinctive O–H stretching band in the IR spectrum at 3665 cm^–1^. The ^1^H NMR spectrum of **6** contains five resonances as expected for a *D*_2h_-symmetric structure, and proton resonances of the hydroxide ligands are not observed in the spectrum. The zero-field Mössbauer spectrum of solid **6** at 80 K has a single quadrupole doublet with *δ* = 0.84 mm s^–1^ and |Δ*E*_Q_| = 1.30 mm s^–1^ indicating an iron(ii) environment with a high-spin electronic configuration. The highly symmetric structure of **6** is confirmed by the solid-state molecular structure shown in Fig. 1 above.

Compound **5** also reacts with 2 equiv. of ^*t*^Bu_3_C_6_H_2_OH to selectively protonate the amide ligands to generate NH_3_ (93%) and the aryloxide compound **2** (73%). Conversely, under similar reaction conditions, treatment of **5** with 2 equiv. of [LutH]Cl does not produce the [LFe(μ-Cl)]_2_ complex that would be expected to result from selective protonation of the amide ligands. Instead, the reaction between **5** and [LutH]Cl produces a complex heterogeneous reaction mixture and no [LFe(μ-Cl)]_2_ is formed in the reaction, although [LFe]_2_(μ-Cl)(μ-NH_2_) is identified in the reaction mixture (further description of this mixed-ligand compound is in the ESI, pp. S16–S17 and Fig. S27†). Overall, our results indicate that the weaker acids ^*t*^Bu_3_C_6_H_2_OH and H_2_O are selective for protonation of the bridging amido ligands, thereby leaving the β-diketiminate Fe fragment intact. Attempts to achieve catalytic turnover with this system are ongoing, but are complicated by the need for K^+^ to form **1**.20*b*

### Reactivity of nitride, imide, and amide complexes with H_2_

In an initial communication, we reported that reaction of **1** with H_2_ in toluene gave [LFe(μ-H)]_2_ (43%) and NH_3_ (42%).20*a* This reaction had been performed in aromatic solvents, and then treated with aqueous HCl after 6 h, followed by testing for NH_4_^+^. However, our further experiments have shown that this method is faulty.^31^ The reaction time before acid treatment had been based on the observation of the reaction of **1** with H_2_ in C_6_D_6_, which is complete (as judged by ^1^H NMR spectroscopy) in 6 h. However, the same reaction in toluene-*d*_8_ requires >24 h to be complete. Since the larger-scale reactions used for NH_4_^+^ detection were done in toluene, but the amount of time before acid treatment was based on the 6 h time determined in benzene, the reaction was not complete at the time of acid addition, and the NH_4_^+^ produced was actually from protonation of unreacted starting material (through the reactions described above). When the reaction between **1** and H_2_ (1 atm) is conducted in aromatic solvents (benzene or toluene) at ambient temperature until complete consumption of **1**, ^1^H NMR analysis of the reaction indicates a complex mixture of products. Two major components are identified by ^1^H NMR spectroscopy as [LFe(μ-H)]_2_ (20–25%) and the reduced iron(i) complex LFe(η^6^-arene) (20–25%), and treatment of the reaction mixture with strong acids produces no NH_4_^+^ or hydrazine.^25^

**Scheme 4:**
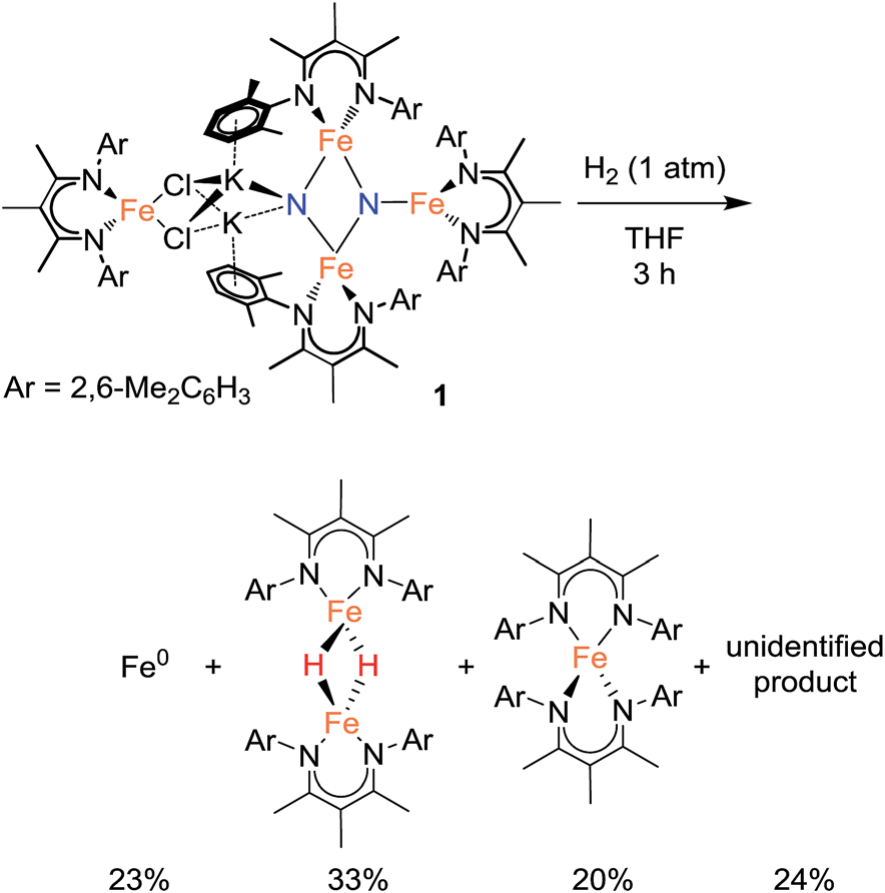
Reactivity of the tetrairon bis(nitride) complex with H_2_: detected products are shown.

Treating **1** with 1 atm H_2_ in THF solvent also produces a mixture of products over 3 h at ambient temperature. Iron metal accounts for 23% of the total Fe content of the reaction products (Scheme 4).^32^ Mössbauer and ^1^H NMR analysis of the THF-soluble products reveal the formation of [LFe(μ-H)]_2_ (33%), L_2_Fe (20%),20*b* and a ^1^H NMR silent species (24%) that is quantified by Mössbauer spectroscopy (Fig. S-12 and S-22†). Attempts to isolate and purify the NMR-silent species have not been successful. Treatment of these species with strong acids produces no detectable amounts of NH_4_^+^ or hydrazine. Regardless of the exact identity of the unknown species, it appears that the nitride ligands from the starting material **1** are no longer present in any of the reaction products. We surmise that nitride coupling leading to N_2_ loss (as shown in our previous work)^26^ dominates in the reaction of **1** with H_2_. This is supported by the observation of LFe(η^6^-C_6_H_6_) as a byproduct from the reaction of **1** with H_2_ in benzene, because LFe(η^6^-C_6_H_6_) is known to be the product from loss of N_2_ from **1**.^26^ The observation that the reaction of **1** with H_2_ is faster in benzene than toluene is consistent with solvent attack being the rate-limiting step, because reaction with the smaller benzene should have a lower barrier.

## Discussion

### Stepwise conversion of nitrides to ammonia

**Scheme 5:**
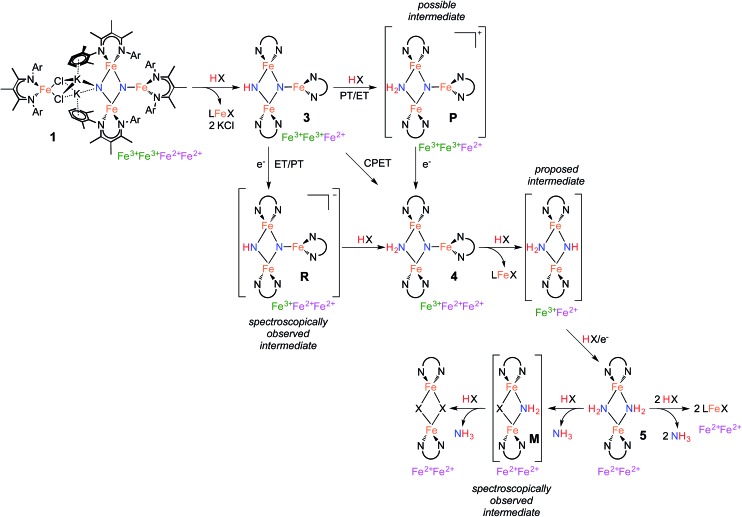
Proposed mechanism of Fe-mediated N_2_ reduction to NH_3_.

The isolation of thermally stable intermediates has allowed us to follow the individual steps in N_2_ functionalization. Scheme 5 shows these transformations, which consist of combinations of proton transfer, electron transfer, and iron loss from the cluster. They culminate in complete conversion of both nitrogen atoms from N_2_ into ammonia. Though we used a number of different acids and reducing agents to accomplish individual steps, note that tri(*t*-butyl)phenol is capable of each individual step, and it also can achieve the complete conversion of the nitrides of **1** into NH_3_ (Table 1 above).

Step 1 in NH_3_ formation selectively protonates one of the two nitride ligands in **1**: the one with two K^+^ ions and no third Fe atom (left N in the structures in Scheme 5). This result shows that the tetrairon structure of **1** can be broken apart at the bridging K^+^ ions leading to two components: a triiron fragment that contains the two N atoms of the activated N_2_, and a monoiron fragment that is no longer involved in the reactivity of the nitride ligands. Interestingly, the triiron nitride/imide **3** is significantly more thermally stable than the precursor **1**. This difference in stability is attributed to the ability of **1** to undergo intramolecular nitride coupling to release N_2_,^26^ which does not occur after proton transfer to form **3**.

Step 2 proceeds by a proton-coupled electron transfer (PCET) process to convert **3** to the triiron nitride/amide **4**. Reactions that transfer both a proton and an electron have a mechanistic ambiguity between concerted proton/electron transfer (CPET) or stepwise proton transfer (PT) and electron transfer (ET).^30^ These mechanisms are difficult to distinguish in many cases, but a key difference is that CPET reactions typically do not build up substantial charge in the transition state, while stepwise mechanisms have an intermediate with different charge. Other common mechanistic tests (*e.g.* Hammett correlations) are often ambiguous because both stepwise and concerted mechanisms can produce similar results.^30^,^33^

The rate of the reaction of H atom donors 2,4,6-tri-*tert*-butylphenol and Cp*2Co with **3** to give **4** has a dependence on solvent polarity, which could suggest a stepwise process for proton and electron transfer. However, the solvent 2,5-dimethyltetrahydrofuran gives a rate similar to that in benzene, despite having a polarity that is similar to that of THF.^28^ Thus, we cannot rule out some specific interaction with solvent as the cause for the rate variation. It should also be borne in mind that the mechanism is not necessarily the same with all substrates. Namely, the conversion of **3** to **4** can be effected by compounds with large homolytic bond energies (*e.g.* H_2_O), which are unlikely to engage in CPET. Further support for stepwise pathways comes from our observation of ET to **3** using KC_8_ or electrochemical reduction. These generate the reduced intermediate **R** with a K^+^ counterion, which then undergoes PT from indene to form the product **4** (Scheme 3). An independent reaction shows that **3** does not react with indene alone, suggesting that when indene is used as the proton source PT occurs only after ET. A similar reaction pathway is possible for the reaction of **3** with Cp*2Co, which would generate the reduced intermediate **R** and Cp*2Co^+^, followed by PT from Cp*2Co^+^ to form **4** and (η^5^-C_5_Me_4_CH_2_)CoCp*. Deprotonation of Cp*2Co^+^ is more facile than the neutral analogue Cp*2Co,^34^ as expected in a stepwise ET/PT pathway. Consistent with the potential intermediacy of **R**, an independent reaction shows that **R** is rapidly protonated upon treatment with [Cp*2Co]PF_6_ to give **4**. The redox potential of Cp*2Co is slightly less negative than **3**, with the Nernst equation implying an equilibrium constant (*K*_ox/red_ = 3.8 × 10^5^) that disfavors the reduction of **3**. Nevertheless, this redox equilibrium is followed by an irreversible PT, which can drive the reaction to product formation. C–H activation of Cp*2Co with an iron–sulfur cluster has been proposed to proceed by a stepwise ET/PT reaction pathway.^35^

Step 3 involves converting the triiron nitride/amide **4** into the diiron bis(amide) **5**. This process formally involves the transfer of two protons and one electron to the complex, with concomitant loss of one of the β-diketiminate iron fragments. Since the conversion of **4** to **5** involves the formation of an amide ligand from a nitride ligand, we propose that this process could proceed by a series of proton and electron transfers analogous to the conversion of bis(nitride) **1** into the triiron nitride/amide **4**, *via* steps 1 and 2 of the mechanism. Despite the required transfer of two protons and one electron to form **5**, no intermediates are observed in this process, indicating that the first step in this multistep transformation is rate limiting. This rate limiting step may be a slow protonation of the sterically congested μ_3_-bridging nitride ligand in **4**. This could form a diiron imide/amide species (Scheme 5) with loss of the monoiron fragment; then, subsequent rapid PCET at the exposed μ_2_-bridging imide would form the bis(amide) product **5**. Alternatively, PCET to **4** could happen first, with loss of the third iron afterwards. We have been unable to design experiments that distinguish between these possibilities.

The final step in NH_3_ formation involves protonation of the amido ligands in **5**, which requires no redox changes. When using a small proton source such as H_2_O, we first observe a mixed amide/hydroxide intermediate **M** (described in the ESI†) that is then protonated a second time to release the second equivalent of NH_3_ (Scheme 5). On the other hand, no intermediates are observed when using bulky proton sources such as ^*t*^Bu_3_C_6_H_2_OH, which result in mononuclear Fe products.

### Comparison to other PCET reactions

There are few examples where proton transfer to N_2_ has been studied in detail.9*a* A number of intermediates have been characterized in the catalytic Mo-based N_2_ reduction system reported by Yandulov and Schrock.^36^ These included the stepwise protonation and reduction of the terminal molybdenum nitride that is an intermediate of N_2_ reduction. With the recently emerging Fe-based catalytic N_2_ reduction systems, stepwise protonation and reduction is important in the mechanism, and mechanistic studies do not implicate nitride intermediates.^37^ The reverse reaction, conversion of NH_3_ to N_2_, was recently described in a mononuclear iridium system where a number of intermediates were characterized.^38^ In that system, the proposed Ir–NH intermediate is thought to undergo rapid disproportionation to Ir nitride and amide species. Conversely, a bimetallic Mo system was shown to react with ammonia to form a stable dimolybdenum bis(μ-imide) complex,^39^ highlighting the increased stability of bridging imido ligands *versus* terminal ones.

Comparisons to other PCET reactions are also relevant.^30^ Though some systems give rapid self-exchange of O–H and N–H bonds on the NMR time scale, the amide N–H in compound **4** does not exchange with that of **3** on the NMR timescale at room temperature, indicating that PCET in this system is relatively slow. Slow self-exchange was reported in a vanadium oxo/hydroxo system (*k* = 6.5 × 10^–3^ M^–1^ s^–1^ at 298 K) and was attributed to large reorganization energy due to the large change in V–O bond lengths in the transition state for hydrogen atom transfer.^40^ Slow self-exchange was attributed to steric crowding in an osmium–aniline system.^41^ In our β-diketiminate-supported Fe system, steric crowding in the transition state could lead to slow self-exchange. Future computational studies will be required to assess the importance of different factors in this system.

Recent research on PCET to oxo complexes has highlighted the balance between basicity and oxidizing ability: a more basic site can be oxidized by a weaker oxidizing agent.^42^ The cyclic voltammetry studies above show that the iron bis(nitride) systems are very weak outer-sphere oxidizing agents, and this implies that they must be highly basic in order to be thermodynamically capable of PCET. This contention is supported by the observation that weak acids such as phenols are capable of bringing about PCET to compound **3**. One way to view this idea in the context of a stepwise PT/ET mechanism is that proton transfer to the Fe–nitride cluster creates a strong enough oxidant to reduce the conjugate base (*e.g.* tri-*tert*(butyl)phenolate). Therefore, as seen in the iron-oxo of cytochrome P450,^42^ the basicity of the reactive species can drive PCET using relatively strong bonds.

### Proton-coupled electron transfer and nitrogenase

Ammonia production in nitrogenase at the iron–molybdenum cofactor (FeMoco) is thought to proceed through the Thorneley–Lowe (TL) scheme, which was established using extensive kinetic studies.^43^ In the TL scheme, each step involves addition of one electron (from the Fe protein) and one proton. Since each electron is delivered to the FeMoco at a similar potential, this suggests that coupled PT and ET are necessary to maintain the required redox potential suitable for subsequent ET to FeMoco.^44^ However, it is not known whether a concerted (CPET) or stepwise (PT/ET or ET/PT) transfer of protons and electrons occurs in nitrogenase.4a,^45^ Although the proposed alternating mechanism of nitrogenase^7^ does not involve the formation of nitride intermediates, it is significant that this work has shown PCET in an iron–N_2_ derived system, and specifically shows that sequential ET to the β-diketiminate Fe system takes place at identical potentials (–2.28 V for compound **3** and –2.27 V for compound **4**) when coupled to PT to balance the charge. This observation supports one fundamental tenet of the Thorneley–Lowe scheme, that protonation of N_2_ intermediates could enable the FeMoco to be reduced at a similar potential during sequential steps. Corroboration of other steps will require synthetic complexes with greater structural resemblance to the biological cofactor.

## Conclusions

A series of iron nitride, imide, and amide complexes have been synthesized and shown to be intermediates during conversion of N_2_ into ammonia at a multi-iron system. Ammonia formation in this system proceeds by a series of six proton transfers and two electron transfers. The β-diketiminate Fe system is distinctive because it gives isolable nitride, imide and amide intermediates that lie along the path to ammonia production from N_2_.

Notably, the Fe nitrides, imides, and amides in this system are very basic and require only very weak acids (p*K*_a_ < 31) to form ammonia, although the p*K*_a_ requirement may be partially offset in many of our reactions by the thermodynamic bias associated with the conjugate base binding to Fe. On the other hand, the electron transfer steps require very strong reducing agents. However, the hydrogen atom transfer from tri(*t*-butyl)phenol shows that PCET can avoid the need for a strong reducing agent. The principles and structures elucidated in this study are expected to be useful for evaluating potential mechanisms for N_2_-reducing electrocatalysts and the nitrogenase enzyme.

## Supplementary Material

Supplementary information

Crystal structure data
