# Supplementary material for: Stepwise N–H bond formation from N2-derived iron nitride, imide and amide intermediates to ammonia
Source: Chem Sci. 2016 Jun 14;7(9):5736–46. doi: 10.1039/c6sc00423g (PMC5207225; doi:10.1039/c6sc00423g)
Supplement: Supplementary file 1 [file SC-007-C6SC00423G-s001.pdf]

Electronic Supplementary Material (ESI) for Chemical Science.
This journal is © The Royal Society of Chemistry 2016

Electronic Supporting Information for:

Stepwise N-H Bond Formation from N_2-Derived Iron Nitride, Imide and Amide Intermediates to Ammonia

*K. Cory MacLeod, Sean F. McWilliams, Brandon Q. Mercado, and Patrick L. Holland**

Table of Contents

Synthesis and characterization **S-3**

- General considerations. S-3
- Independent Synthesis of $LFe(OC_6H_2{}^tBu_3)$ (**2**). S-5
- Synthesis of $[LFe]_2(\mu_2\text{-}NH)(\mu_3\text{-}N)[FeL]$ (**3**). S-5
- Synthesis of $[LFe]_2(\mu_2\text{-}NH_2)(\mu_3\text{-}N)[FeL]$ (**4**). S-6
- Independent synthesis of $[LFe(\mu\text{-}NH_2)]_2$ (**5**). S-7
- Independent synthesis of $[LFe(\mu\text{-}OH)]_2$ (**6**). S-7
- Independent Synthesis of $[LFe(\mu\text{-}C\equiv CPh)]_2$ (**7a**). S-8
- Characterization of the $[LFe\{\mu\text{-}C\equiv CC_6H_2(CF_3)_2\}]_2$ co-product (**7b**). S-9
- Independent Synthesis of $LFe(\eta_5\text{-}C_9H_7)$ (**8**). S-9
- In situ formation of intermediate $K[\{LFe\}_2(\mu_2\text{-}NH)(\mu_3\text{-}N)\{FeL\}]$ (**R**). S-10
- General procedure for reactions in Tables 1 and 2. S-10
- General procedure for reactions in Table 3. S-11
- General procedure for reactions in Table S-1. S-12
- General procedure for reactions in Table S-2. S-12
- Reaction of bis(nitride) (**1**) with H_2. S-13
- Discussion of independent synthesis and characterization of LFeX by-products. S-13
- Discussion of amide, hydride, and hydroxide ligand exchange. S-16
- Definitive characterization of N_2-derived iron complexes with nitride, imide, amide and hydroxide ligands. S-19

**Mössbauer spectra**	**S-21**
**NMR spectra**	**S-29**
**IR spectra**	**S-51**
**UV-visible spectra**	**S-59**
**Cyclic voltammetry**	**S-63**
**X-ray crystallographic information**	**S-66**
**References**	**S-71**

General Considerations. Unless otherwise noted, all manipulations were performed under an N_2 atmosphere using Schlenk techniques or in an M. Braun glovebox maintained at or below 1 ppm of O_2 and H_2O. Glassware was dried at 150 °C overnight. Celite, alumina, and 4Å molecular sieves were dried at 200 °C under vacuum overnight. Pentane, hexanes, diethyl ether, and toluene were purified by passage through activated alumina and Q5 columns from Glass Contour Co. THF was distilled under argon from a potassium benzophenone ketyl solution. All solvents were stored over activated 4Å molecular sieves. Benzene-d_6 was dried and stored over activated alumina and then filtered before use. Toluene-d_8 and THF-d_8 were dried by vacuum transfer from potassium benzophenone ketyl solution and were stored over 4Å molecular sieves. PhC≡CMgBr (1.0 M in THF) was purchased from Aldrich and used as received. $LiNH_2$ (95%) was purchased from Acros Organics and used as received. 1-Ethynyl-3,5-bis(trifluoromethyl)benzene (97%), was purchased from Aldrich and degassed by three freeze-vacuum-thaw cycles and stored over 4Å molecular sieves. Indene was dried by vacuum transfer from calcium hydride and was stored over 4Å molecular sieves at -40 °C. $^tBu_3C_6H_2OH$ was dried by dissolving in hexanes and storing it over 4Å molecular sieves for 6 h before filtering through Celite and cooling to -40 °C to give a precipitate that was isolated.

NaC_9H_7 was prepared by mixing indene with 1 equiv of $NaN(SiMe_3)_2$ in toluene at -40 °C and stirring at ambient temperature for 1 h. The resulting reaction mixture was filtered to remove a pale yellow solid that was rinsed with toluene and pentane before being dried under vacuum to yield pure NaC_9H_7 as a pale yellow solid that was stored at -40 °C prior to use. The following compounds were prepared according to published procedures: $KOC_6H_2{}^tBu_3$,[1] •$OC_6H_2{}^tBu_3$,[2] TEMPOH,[3] [LFe(μ-Cl)]$_2$,[4] [LFe(μ-H)]$_2$,[5] [$LFeCl_2$](K)$_2$[LFe]$_2$(μ_2-N)(μ_3-N)[FeL] (**1**), and [$LFeCl_2$](K)$_2$[LFe]$_2$(μ_2-^{15}N)(μ_3-^{15}N)[FeL] (**1-$^{15}N_2$**).[5]

^1H and ^{19}F NMR data were recorded on Agilent DD2 400 MHz or 500 MHz spectrometers. All resonances in the ^1H NMR spectra are referenced to residual protio solvents: benzene (δ 7.16 ppm), toluene (δ 2.09 ppm), or THF (δ 3.58 or 1.73 ppm). Resonances were singlets unless otherwise noted. IR data were recorded on a Bruker ALPHA spectrometer equipped with a Platinum-ATR attachment. UV-vis spectra were recorded on a Cary 60 spectrophotometer using Schlenk-adapted quartz cuvettes with a 1 mm optical path length. Solution magnetic susceptibilities were determined by the Evans method.[6] Mössbauer data were recorded on a SeeCo spectrometer with alternating constant acceleration; isomer shifts are relative to iron metal at 298 K. The sample temperature was maintained constant in a Janis Research Company Inc. cryostat. The zero-field spectra were simulated by using Lorentzian doublets with γ representing the line width fitting parameter (γ_L and γ_R are described when left and right line widths are inequivalent). Elemental analyses were obtained from the CENTC Elemental Analysis Facility at the University of Rochester. Microanalysis samples were weighed with a PerkinElmer Model AD-6 Autobalance and their compositions were determined with a PerkinElmer 2400 Series II Analyzer, and handled in a VAC Atmospheres glovebox under argon.

Cyclic voltammetry data were recorded using a PINE WaveNow potentiostat inside an argon-filled glovebox. A Pt ceramic patterned electrode was used as the working electrode. A Pt wire electrode was used as the counter electrode. A silver electrode was used as a quasireference. The electrolyte was a 0.1 M solution of tetrabutylammonium hexafluorophosphate in dry THF. Potentials were referenced to the $Cp_2Fe^{0/+}$ couple using an internal ferrocene standard.

Headspace gas analyses were performed using a Thermo Scientific Trace 1300 gas chromatograph. Samples (200 μL) were injected into an SSL injection port. The samples ran through a mol sieve 5 Å PLOT capillary GC column (30 m length, 0.53 mm inner diameter, 30 μm

average thickness) purchased from Sigma-Aldrich at 0.95 mL/min flow of N_2 carrier gas and a constant oven temperature of 35 °C. Samples were detected using a TCD detector set to negative polarity.

Independent Synthesis of LFe($OC_6H_2{}^tBu_3$) (2). A solution of $KOC_6H_2{}^tBu_3$ (115.5 mg, 0.384 mmol) in THF (4 mL) was added dropwise to a stirred solution of [LFe(μ-Cl)]$_2$ (152.6 mg, 0.186 mmol) in THF (6 mL). The reaction mixture rapidly changed color from yellow to orange. After 5.5 h, the mixture was dried under vacuum. Toluene (5 mL) was added and the mixture was again dried under vacuum to remove any residual THF. The orange residue was extracted with pentane (12 mL), filtered through Celite, and concentrated to 5 mL. The resulting solution was cooled to -40 °C to yield orange crystals (44.8 mg). The mother liquor was concentrated to 3 mL and returned to the freezer for additional product crystallization. The total isolated yield was 169 mg (72%). ^{1}H NMR (C_6D_6, 25 °C): δ 206 (3H), 108 (2H), 24 (9H), 17 (6H), -9.2 (4H), -14 (18H), -54 (12H), -77 (2H) ppm. μ_{eff} (C_6D_6, 25 °C) 5.5(1) μ_B. IR (cm^{-1}): 3067 (w), 2947 (m), 2901 (m), 2865 (m), 1518 (m), 1459 (m), 1425 (s), 1382 (w), 1355 (m), 1322 (s), 1280 (s), 1245 (m), 1220 (w), 1190 (m), 1164 (w), 1125 (w), 1095 (w), 1029 (w), 988 (m), 925 (w), 892 (w), 858 (s), 800 (w), 777 (w), 758 (s), 702 (w), 642 (w), 618 (w), 555 (m), 501 (w), 491 (w), 475 (w), 438 (w), 418 (w), 405 (w). UV-vis (hexanes; λ_{max}, nm (ε, mM^{-1}cm^{-1})): 346 (16.8), 397 (6.5), 520 (sh, 0.3). Anal. Calcd for $C_{40}H_{56}N_2OFe$: C, 75.46; H, 8.86; N, 4.40. Found: C, 75.57; H, 9.21; N, 4.26.

Synthesis of [LFe]$_2$(μ_2-NH)(μ_3-N)[FeL] (3). A solution of **1** · 2 hexane (251 mg, 0.136 mmol) in toluene (12 mL) was cooled to -78 °C. $(CF_3)_2C_6H_2CCH$ (24.5 μL, 0.138 mmol) was added dropwise while stirring. The resulting reaction mixture was then stirred at ambient temperature for 1 h before being dried under vacuum. The residue was extracted with toluene (8 mL), filtered over Celite, and rinsed with toluene (2 × 1 mL) to remove black insoluble precipitate (further

characterized below). The resulting dark brown-orange solution was concentrated to 5 mL and again filtered through Celite. The solution was then layered with pentane (8 mL) and cooled to -40 °C for 2 d. The mother liquor was then concentrated to 4 mL, filtered over Celite to remove a small amount of precipitate, layered with pentane (8 mL) and returned to the freezer. After 4 d, a single crop of black crystalline **3** was isolated (103 mg, 66%). ^{1}H NMR (C_6D_6, 25 °C): δ 114 (3H), 12 (4H), 11 (4H), 9.1 (12H), 2.1 (residual toluene), 1.5 (6H), 1.2 (residual pentane), 0.9 (residual pentane), 0.3 (12H), -3.8 (4H), -5.8 (4H), -9.1 (6H), -19 (12H), -32 (2H), -59 (12H), -100 (1H) ppm. μ_{eff} (C_6D_6, 25 °C) 4.0(1) μ_B. IR (cm^{-1}): 3344 (w, $\nu_{N\text{-}H}$), 3063 (w), 3012 (w), 2968 (w), 2914 (m), 2853 (w), 1591 (w), 1526 (m), 1461 (m), 1437 (w), 1409 (m), 1362 (w), 1328 (s), 1289 (m), 1254 (w), 1219 (w), 1189 (m), 1161 (w), 1132 (w), 1091 (m), 1032 (w), 988 (w), 918 (w), 884 (w), 856 (m), 800 (m), 760 (s), 716 (w), 702 (m), 647 (m), 619 (w), 561 (w), 531 (w), 495 (w), 460 (w). EPR: silent at 9 K. UV-vis (toluene; λ_{max}, nm (ε, mM^{-1}cm^{-1})): 332 (33.8), 420 (sh, 12.8). Anal. Calcd for $C_{66}H_{82}N_8Fe_3$: C, 68.64; H, 7.16; N, 9.70. Found: C, 68.68; H, 7.50; N, 9.37.

Synthesis of [LFe]$_2$(μ_2-NH$_2$)(μ_3-N)[FeL] (4). A solution of Cp*$_2$Co (22.6 mg, 0.0686 mmol) in toluene (2 mL) was added dropwise to a stirred solution of **3** (75.1 mg, 0.0650 mmol) in toluene (6 mL). After 4 h, the mixture was dried under vacuum. Hexanes (2 mL) was added and the mixture was again dried under vacuum to remove any residual toluene. The brown residue was rinsed with hexanes (4 × 2 mL) to remove soluble by-products, and the remaining brown solid was dried under vacuum to provide analytically pure **4** (60 mg, 80%). ^{1}H NMR (C_6D_6, 25 °C): δ 112 (3H), 30 (6H), 19 (12H), 9.7 (4H), 4.3 (4H), 2.1 (residual toluene), 0.7 (6H), -8.0 (br overlapping, 4H), -12 (br overlapping, 12H), -13 (br overlapping, 12H), -31 (2H), -35 (br overlapping, 12H), -51 (10H, Cp_2Co internal standard), -167 (4H) ppm. μ_{eff} (C_6D_6, 25 °C) 4.7(2) μ_B. IR (cm^{-1}): 3375 (w, $\nu_{N\text{-}H}$), 3299 (w, $\nu_{N\text{-}H}$), 3062 (w), 3011 (w), 2955 (m), 2912 (m), 2850 (m), 2760 (w), 2740 (w), 1590 (w),

1518 (m), 1461 (m), 1436 (m), 1408 (m), 1362 (m), 1329 (s), 1289 (m), 1253 (w), 1218 (w), 1189 (m), 1160 (w), 1131 (w), 1088 (m), 1031 (w), 988 (m), 955 (w), 918 (w), 886 (w), 856 (m), 800 (m), 761 (s), 727 (w), 702 (w), 638 (w), 616 (m), 572 (w), 524 (w), 497 (m), 448 (m). EPR: silent at 9 K. UV-vis (toluene; λ_{max}, nm (ε, $mM^{-1}cm^{-1}$)): 345 (32.0). Anal. Calcd for $C_{66}H_{83}N_8Fe_3$: C, 68.58; H, 7.24; N, 9.69. Found: C, 68.78; H, 7.23; N, 9.24.

Independent synthesis of [LFe(μ-NH_2)]$_2$ (5). A suspension of $LiNH_2$ (9.2 mg, 0.40 mmol) in THF (2 mL) was added dropwise to a stirred solution of [LFe(μ-Cl)]$_2$ (155 mg, 0.189 mmol) in THF (8 mL). After 30 h, the mixture was dried under vacuum. The residue was extracted with toluene (8 mL) and filtered through Celite. The resulting dark yellow solution was cooled to -40 °C overnight to yield a yellow crystalline solid (21 mg). The mother liquor was concentrated to 5 mL and returned to the freezer for additional product crystallization. The total isolated yield was 94 mg (57%). ^1H NMR (C_6D_6, 25 °C): δ 53 (6H), 10 (8H), 6.2 (24H), 2.1 (3H, toluene), -14 (4H), -19 (12H) ppm. μ_{eff} (C_6D_6, 25 °C) 4.5(1) μ_B. IR (cm^{-1}): 3365 (w, ν_{N-H}), 3065 (w), 3018 (w), 2962 (w), 2940 (w), 2913 (m), 2854 (w), 1591 (w), 1521 (m), 1461 (m), 1411 (m), 1363 (m), 1341 (s), 1294 (m), 1252 (w), 1224 (w), 1194 (m), 1161 (w), 1135 (w), 1087 (m), 1029 (w), 992 (m), 915 (w), 890 (w), 857 (m), 800 (m), 759 (s), 728 (m), 715 (w), 694 (w), 648 (m), 638 (m), 618 (w), 574 (w), 533 (w), 494 (m), 464 (w), 409 (m). UV-vis (toluene; λ_{max}, nm (ε, $mM^{-1}cm^{-1}$)): 343 (28.3), 455 (sh, 2.7). Anal. Calcd for $C_{44}H_{58}N_6Fe_2 \cdot C_7H_8$: C, 70.02; H, 7.60; N, 9.61. Found: C, 69.92; H, 7.50; N, 9.26.

Independent synthesis of [LFe(μ-OH)]$_2$ (6). H_2O (0.48 mL of 0.55 M solution in THF, 0.26 mmol) was added to a stirred solution of [LFe(μ-H)]$_2$ (99.4 mg, 0.132 mmol) in THF (6 mL). After 15 min, the mixture was dried under vacuum. Pentane (2 mL) was added and the mixture was again dried under vacuum to remove any residual THF. The residue was extracted with pentane (18 mL) and filtered through Celite. The resulting yellow-green solution was cooled to -40 °C overnight to

yield a green crystalline solid (56 mg). The mother liquor was concentrated to 8 mL and returned to the freezer for a second crop of product (17 mg). The total isolated yield was 73 mg (70%). ^{1}H NMR (C_6D_6, 25 °C): δ 141 (6H), 9.2 (8H), 6.2 (24H), -38 (12H), -53 (4H) ppm. μ_{eff} (C_6D_6, 25 °C) 7.1(2) μ_B. IR (cm^{-1}): 3665 (w, $\nu_{O\text{-}H}$), 3064 (w), 3035 (w), 3016 (w), 2964 (w), 2941 (w), 2911 (w), 2852 (w), 1590 (w), 1529 (m), 1460 (m), 1435 (m), 1412 (m), 1365 (m), 1339 (s), 1300 (w), 1252 (w), 1226 (w), 1193 (m), 1162 (w), 1133 (w), 1092 (w), 1030 (w), 989 (m), 953 (w), 917 (w), 892 (w), 859 (w), 800 (w), 759 (s), 713 (m), 639 (w), 617 (w), 493 (w), 450 (m). UV-vis (hexanes; λ_{max}, nm (ε, mM^{-1}cm^{-1})): 341 (27.9). Anal. Calcd for $C_{44}H_{56}N_4O_2Fe_2$: C, 67.35; H, 7.19; N, 7.14. Found: C, 67.44; H, 7.09; N, 6.97.

Independent Synthesis of [LFe(μ-C≡CPh)]$_2$ (7a). PhC≡CMgBr (0.35 mL of 1.0 M solution in THF, 0.35 mmol) was added dropwise to a stirred suspension of [LFe(μ-Cl)]$_2$ (103.8 mg, 0.126 mmol) in Et_2O (12 mL) at -40 °C. The reaction mixture turned dark red over a period of 5 min. The resulting mixture was stirred at ambient temperature for an additional 1.5 h before addition of 1,4-dioxane (110 μL, 1.29 mmol). The mixture was then stirred for an additional 10 min and then dried under vacuum. The residue was extracted with toluene (10 mL), filtered through Celite, and concentrated to 8 mL. The red solution was then filtered again over Celite before being cooled to -40 °C for 6 days to yield 102 mg (78% yield) dark red crystalline solid. ^{1}H NMR (C_6D_6, 25 °C): δ 197 (6H), 30 (br overlapping, 24H), 28 (br overlapping, 8H), 23 (4H), 2.1 (3H, toluene), -37.6 (12H), -38.4 (4H), -46 (2H), -52 (4H) ppm. μ_{eff} (C_7D_8, 25 °C) 7.2(3) μ_B. IR (cm^{-1}): 3054 (w), 3023 (w), 2953 (m), 2913 (m), 2847 (m), 2779 (w), 2737 (w), 1891 (m, $\nu_{C\equiv C}$), 1604 (m), 1568 (w), 1507 (m), 1462 (m), 1438 (m), 1404 (m), 1383 (w), 1363 (w), 1324 (s), 1290 (m), 1254 (m), 1217 (w), 1190 (m), 1163 (w), 1129 (w), 1091 (w), 1069 (w), 1030 (w), 989 (m), 961 (w), 917 (w), 892 (w), 861 (m), 798 (w), 764 (s), 753 (s), 727 (m), 707 (w), 689 (m), 638 (w), 619 (w), 576 (w), 561 (w),

527 (w), 495 (m), 463 (w), 414 (w). UV-vis (toluene; λ_{max}, nm (ε, $mM^{-1}cm^{-1}$)): 341 (32.4), 395 (sh, 11.0), 548 (5.1). Anal. Calcd for $C_{60}H_{64}N_4Fe_2 \cdot C_7H_8$: C, 77.01; H, 6.94; N, 5.36. Found: C, 76.87; H, 7.07; N, 5.24.

Characterization of the [LFe{μ-C≡$CC_6H_2(CF_3)_2$}]$_2$ co-product (7b). The black insoluble precipitate described above in the synthesis of **3** was extracted with THF (5 mL), filtered over Celite, and the solution was dried under vacuum to provide 46.5 mg of a black solid. The ^{1}H NMR spectrum solution of crude solid in C_6D_6 indicated the presence of [LFe{μ-C≡$CC_6H_2(CF_3)_2$}]$_2$ as the major component, in addition to a small amount of **3**. Resonances attributed to [LFe{μ-C≡$CC_6H_2(CF_3)_2$}]$_2$ are listed (see Figure S-19). ^{1}H NMR (C_6D_6, 25 °C): δ 47 (br overlapping, 24H), 43 (br overlapping, 8H), -57 (12H), -62 (4H), -67 (2H) ppm. ^{19}F NMR (C_6D_6, 25 °C): δ -89 ppm. IR (cm^{-1}): 3068 (w), 2965 (w), 2915 (w), 2855 (w), 2769 (w), 1876 (m, $\nu_{C\equiv C}$), 1612 (w), 1592 (w), 1515 (m), 1461 (m), 1439 (w), 1408 (m), 1368 (m), 1327 (s), 1292 (w), 1275 (s), 1217 (w), 1189 (w), 1172 (s), 1124 (s), 1106 (w), 1094 (m), 1034 (w), 987 (m), 919 (w), 902 (w), 891 (m), 862 (m), 845 (m), 798 (w), 762 (m), 724 (w), 697 (m), 681 (m), 638 (w), 618 (w), 566 (w), 540 (w), 517 (m), 499 (m), 464 (w), 438 (w), 414 (w).

Independent Synthesis of LFe(η_5-C_9H_7) (8). A solution of NaC_9H_7 (34.4 mg, 0.249 mmol) in THF (1 mL) was added dropwise to a stirred solution of [LFe(μ-Cl)]$_2$ (100.5 mg, 0.122 mmol) in THF (5 mL). The reaction mixture rapidly changed color from yellow to orange-red. After 1 h, the mixture was dried under vacuum. The residue was extracted with hexanes (12 mL), filtered through Celite, and concentrated to 8 mL. The resulting solution was cooled to -40 °C overnight to yield orange-red crystals (81.2 mg). The mother liquor was concentrated to 1 mL and returned to the freezer for a second crop of product (18.9 mg). The total isolated yield was 100 mg (83%). ^{1}H NMR (C_6D_6, 25 °C): δ 121 (3H), 49 (2H), 15 (1H), 0.3 (2H), -1.4 (4H), -2.5 (6H), -27 (12H), -37

(2H), -55 (2H) ppm. μ_{eff} (C_6D_6, 25 °C) 3.5(1) μ_B. IR (cm^{-1}): 3063 (w), 3032 (w), 3012 (w), 2961 (w), 2911 (m), 2848 (w), 2723 (w), 1587 (w), 1520 (m), 1442 (m), 1411 (m), 1367 (m), 1333 (s), 1295 (m), 1253 (w), 1243 (w), 1196 (m), 1163 (w), 1133 (w), 1088 (m), 1034 (m), 1000 (m), 955 (w), 940 (w), 908 (w), 889 (m), 863 (m), 796 (m), 761 (s), 751 (s), 711 (m), 652 (m), 620 (w), 570 (w), 555 (w), 543 (w), 498 (m), 452 (m), 430 (w), 421 (w). UV-vis (toluene; λ_{max}, nm (ε, $mM^{-1}cm^{-1}$)): 313 (17.2), 383 (sh, 6.6). Anal. Calcd for $C_{31}H_{34}N_2Fe$: C, 75.91; H, 6.99; N, 5.71. Found: C, 75.99; H, 7.10; N, 5.48.

In situ formation of intermediate K[{LFe}$_2$(μ_2-NH)(μ_3-N){FeL}] (R). A solution of **3** (6.4 mg, 0.0055 mmol) in toluene-d_8 (0.50 mL) was cooled to -78 °C, followed by addition of KC_8 (1.0 mg, 0.0074 mmol) while stirring. The reaction mixture was stirred at ambient temperature for 10 min. The mixture was then filtered through Celite into a J. Young NMR tube and characterized by ^1H NMR spectroscopy. ^1H NMR (C_7D_8, 25 °C): δ 113 (3H), 27 (6H), 16 (12H), 12 (4H), 9.8 (4H), -3.1 (4H), -11 (12H), -13 (br overlapping, 4H), -13 (br overlapping, 6H), -32 (2H), -40 (12H), -51 (10H, Cp_2Co internal standard), -62 (12H) ppm.

General procedure for reactions in Tables 1 and 2. A solution of **1** · 2 hexane (25 mg, 0.014 mmol) in THF (4 mL) in a 25 mL Schlenk flask was cooled in a -96 °C bath, followed by addition of the indicated acid (12 equiv) while stirring under a flow of N_2. The resulting reaction mixture was immediately removed from the cold bath and allowed to warm to ambient temperature and stirred overnight. The resulting reaction mixture was then dried under vacuum. The residue was extracted in a phosphate buffer solution (50 mM, pH 7.0) and filtered through Celite. The indophenol method was then used for NH_3 quantification.[7] Samples were also tested for hydrazine.[8] The following acids were added as neat substances: H_2SO_4 (95%), [NBu_4]HSO_4 (99%), HCl (36.5-38%), H_3PO_4 (85%), HNO_3 (69-70%), [pyH]Cl, [lutH]Cl, HOTs·H_2O (98%), CF_3CO_2H (99%) and

$C_6H_5CO_2H$ (99%). The following acids were pre-dissolved in THF: [lutH]BAr^F_4 (83 mM), $^tBu_3C_6H_2OH$ (44 mM), H_2O (0.55 M).

Procedure for formation of $^{15}NH_4Cl$. A solution of **1-$^{15}N_2$** · 2 hexane (26 mg, 0.014 mmol) in THF (4 mL) in a 20 mL bomb flask was cooled in a -96 °C bath, followed by addition of neat H_2SO_4 (95%) (12 equiv) while stirring under a flow of N_2. The resulting reaction mixture was immediately removed from the cold bath and allowed to warm to ambient temperature and stirred overnight. The resulting reaction mixture was then dried under vacuum. Solid potassium *tert*-butoxide was added to the flask, and 3 mL of THF was vac transferred onto the solid. The reaction mixture was allowed to stir overnight at ambient temperature. The volatiles were vacuum transferred onto a 2 M HCl solution in diethyl ether (0.7 mL) and allowed to stir overnight. The volatiles were removed under vacuum and the remaining solid was dissolved in 0.7 mL of DMSO-d_6. Yield of $^{15}NH_4Cl$ was calculated against an internal standard capillary of 1,3,5-trimethoxybenzene in C_6D_{12} showing 90% yield of $^{15}NH_4Cl$. From **1**: ^1H NMR (400 MHz, DMSO-d_6): δ 7.2 (t, J = 50 Hz). From **1-$^{15}N_2$**: ^1H NMR (400 MHz, DMSO-d_6): δ 7.2 (d, J = 72 Hz). See Figures S-28 and S-29.

General procedure for reactions in Table 3. A solution of **1** · 2 hexane (8.0 mg, 0.0043 mmol) in toluene-d_8 (0.50 mL) was cooled to -78 °C, followed by addition of the indicated acid (1.0 equiv) while stirring. After 10 min, the resulting reaction mixture was then stirred at ambient temperature for an additional 10 min. The mixture was then filtered through Celite into a J. Young NMR tube. Reaction yields were determined by ^1H NMR using a Cp_2Co internal capillary standard (δ -51 ppm). The following acids were added as neat substances: $C_6H_5C{\equiv}CH$, $(CF_3)_2C_6H_2C{\equiv}CH$, $CH_3(CH_2)_5C{\equiv}CH$, TEMPOH, [lutH]Cl, [lutH]BAr^F_4, $C_6H_5CO_2H$, indene, $^tBu_3C_6H_2OH$, and MeOH. H_2O was added as a 0.55 M solution in THF.

General procedure for reactions in Table S-1. The indicated acid (12 equiv) was added to a solution of **1** · 2 hexane (30 mg, 0.016 mmol) in THF (4 mL) while stirring. A rapid color change was observed, typically to pale yellow. After stirring for approximately 1 h, the reaction mixture was cooled in a frozen acetone bath (-96 °C) followed by the addition of H_2SO_4 (12 equiv). The reaction mixture was stirred overnight at ambient temperature and was then dried under vacuum. The residue was extracted in a phosphate buffer solution (50 mM, pH 7.0) and filtered through Celite. The indophenol method was then used for NH_3 quantification.[7] Samples were also tested for hydrazine.[8]

Table S-1. Sequential treatment of **1** with a weak acid followed by H_2SO_4.

Weak acid	$[NH_4]^+$ yield without addition of H_2SO_4 (from Table 1)	$[NH_4]^+$ yield with subsequent addition of H_2SO_4
$HOTs{\cdot}H_2O$	79%	72%
[LutH]Cl	39%	44%
[pyH]Cl	7%	13%
$C_6H_5CO_2H$	12%	50%

General procedure for reactions in Table S-2. Manometry experiments with **1** were performed by adding the indicated acid (12 equiv) to a round-bottom flask containing a solution of **1** · 2 hexane (30 mg, 0.016 mmol) in THF (4 mL) while simultaneously monitoring the pressure with a mercury-filled U-tube composed of a 1/16 inch Tygon tube attached to a ruler. The reactions were stirred at ambient temperature until the pressure stabilized (1-3 h). The reactions were repeated in a separate, sealed round-bottom flask. After stirring for 1 h, 400 µL of CH_4 (internal standard) was added to the reactions and an aliquot of the reaction headspaces were analyzed by GC to quantify the amount of H_2 produced.

Table S-2. Monitoring gas production upon treatment of **1** with weak acids.

Acid	Product distribution				
	Equiv $[NH_4]^+$	Equiv gas produced	Equiv H_2	Remaining equiv gas (assumed to be N_2)	Total N content accounted for
HOTs·H_2O	1.44 (72%)	0.71	0.01	0.70	142%
[LutH]Cl	0.88 (44%)	0.74	0.24	0.50	94%
[pyH]Cl	0.26 (13%)	1.40	1.47	0	13%
$C_6H_5CO_2H$	1.00 (50%)	0.49	0.12	0.37	87%

The results in Table S-2 have large uncertainties associated with them, because quantitating these small amounts of gases is difficult.

Reaction of bis(nitride) (1) with H_2. Compound **1** · 2 hexane (78 mg, 0.042 mmol) was dissolved in THF (4 mL) in a thick-walled glass reaction vessel. The solution was frozen, the headspace was evacuated and then backfilled with H_2 (1 atm). Upon thawing, the solution was stirred at ambient temperature for 3 h, during which Fe metal precipitated from the reaction. The reaction mixture was filtered to remove the Fe metal, which was then dissolved in HCl (3 M) and quantified by spectrophotometric methods (23% of the total Fe content).[9] The remaining reaction solution was then dried under vacuum and the solid residue was analyzed by Mössbauer spectroscopy (see Figure S-12) and ^{1}H NMR spectroscopy (see Figure S-22) to quantify the remaining Fe containing species.

Discussion of independent synthesis and characterization of LFeX by-products. The stoichiometric protonation of bis(nitride) **1** produces the triiron nitride/imide product **3** and a by-product that results from the dangling iron(II) center in **1** being lost from the cluster upon formation of **3**. The identity of the iron(II) by-product varies depending on the choice of proton source. The LFeX by-products were therefore synthesized independently and characterized, for identification

and quantitation in the stoichiometric protonation studies. Compounds **2**, **7a**, and **8** were prepared by reacting $[LFe(\mu\text{-}Cl)]_2$ with $KOC_6H_2{}^tBu_3$, PhC≡CMgBr, or NaC_9H_7, respectively (Scheme S-1).

Scheme S-1. Independent synthesis of compounds **2**, **7a**, and **8** from a common precursor $[LFe(\mu\text{-}Cl)]_2$.

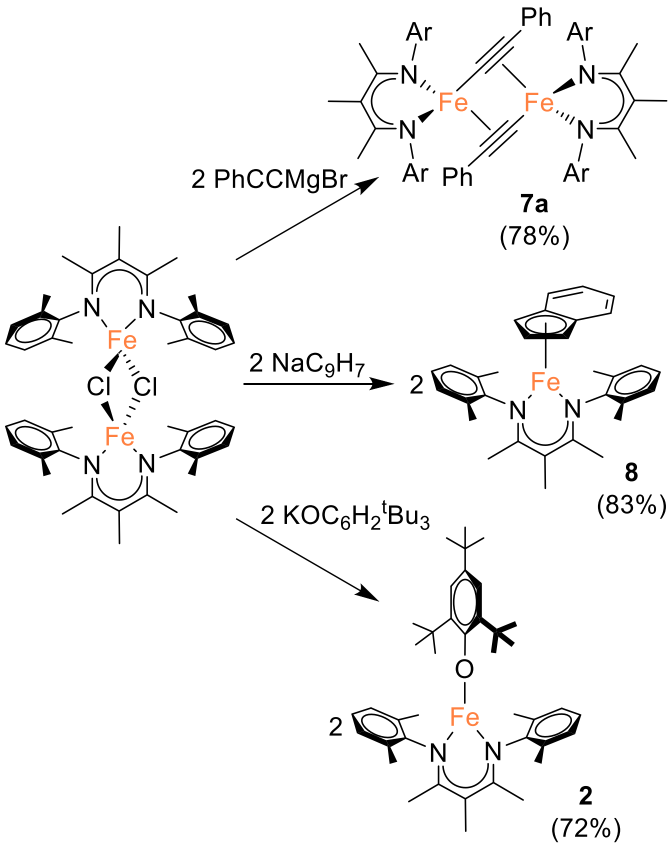

Compound **2** contains a three-coordinate Fe center with approximate trigonal planar geometry where the aryloxide ligand is leaning slightly toward one side of the β-diketiminate ligand. As a result, the O14–Fe1–N11 bond angle of 125.94(9)° is 15° smaller than the O14–Fe1–N21 angle of 141.50(9)°. The low coordination number at Fe is attributed to the steric influence of the aryloxide ligand, which prevents dimerization that is otherwise common with smaller ligands (hydride,

hydroxide, chloride, amide, and alkynyl) with this β-diketiminate Fe system. The solution magnetic moment of **2** in C_6D_6 is 5.5(1) μ_B, and the zero-field Mössbauer spectrum of solid **2** at 173 K has δ = 0.77 mm/s and $|\Delta E_Q|$ = 1.38 mm/s, consistent with a high-spin (S = 2) electronic configuration at the iron(II) ion. Upon addition of THF, pyridine, or NH_3, the color of **2** changes from orange to yellow and the resonances in the ^1H NMR spectrum broaden (Figures S-24 and S-25). This suggests that **2** is capable of binding an additional ligand to form four coordinate species, as observed with other β-diketiminate-iron complexes.[10]

This ability of **2** to form a complex with NH_3 complicated the analysis of reactions between **5** and $^tBu_3C_6H_2OH$, which produce both **2** and NH_3. Figure S-24 below shows the ^1H NMR spectrum of such a mixture, which gives broadened peaks. Initial experiments used vacuum transfer of volatile materials at 80 °C for 15 minutes, but these experiments gave only 79% yield of ammonia from the indophenol test. Control experiments showed that this temperature was not sufficient to drive NH_3 off of samples of **2**. Therefore, later experiments heated the reaction mixture to 100 °C for 20 minutes during the vacuum transfer. After this treatment, we obtained 93% yield of NH_3 (as given in the text of the paper), and the remaining **2** had sharp peaks that are characteristic of a NH_3-free sample.

The solid-state molecular structure of the bridging alkynyl complex **7a** displays an approximately tetrahedral geometry at the two Fe centers, with μ-η^2:κ_{C2}-C≡CPh bridging ligands. The zero-field Mössbauer spectrum of solid **7a** at 173 K has δ = 0.54 mm/s and $|\Delta E_Q|$ = 1.49 mm/s (**7b** has δ = 0.52 mm/s, $|\Delta E_Q|$ = 1.84 mm/s), consistent with a high-spin (S = 2) iron(II) center. The solution magnetic moment of 7.2(3) μ_B for **7a** indicates that the two high-spin iron(II) centers in the dimer have little exchange coupling at room temperature (expected μ_{eff} for two uncoupled high-spin iron(II) = $\sqrt{2} \times 4.9 = 6.9$ μ_B). The IR spectrum of **7a** contains a band in the C≡C stretching region

(1891 cm^{-1}), and the *m*-CF_3 substituted analogue **7b** has a band at slightly lower energy (1876 cm^{-1}). The C≡C stretching vibration in these complexes is approximately 100 cm^{-1} lower in energy than other high-spin iron(II) compounds having terminally-bound alkynyl ligands,[11] consistent with backbonding from the η^2-bound Fe center into the π^* orbital of the alkynyl ligand.

The solid-state molecular structure of **8** reveals η^5-coordination of the indenyl ligand to the Fe center with Fe–C bond lengths ranging from 2.113(6) to 2.432(6) Å. The zero-field Mössbauer spectrum of solid **8** at 80 K has δ = 0.67 mm/s and $|\Delta E_Q|$ = 1.07 mm/s. The low isomer shift and the solution magnetic moment for **8** of 3.5(1) μ_B are most consistent with an intermediate-spin (S = 1) electronic configuration at the iron(II) ion. The ^{1}H NMR spectrum of **8** contains nine resonances with integrations that are consistent with averaged C_{2v} symmetry, which suggests rapid rotation on the NMR timescale of the indenyl ligand and/or the aryl groups of the β-diketiminate ligand.

Discussion of amide, hydride, and hydroxide ligand exchange. The compound $[LFe(\mu\text{-}OH)]_2$ (**6**) can be prepared independently by treating $[LFe(\mu\text{-}H)]_2$ with 2 equiv of H_2O (Scheme S-2). (Interestingly, adding only 1 equiv of H_2O to $[LFe(\mu\text{-}H)]_2$ produces a mixture of starting material and **6** in a 1:1 ratio, with no mixed bridging hydride/hydroxide species formed.) When $[LFe(\mu\text{-}H)]_2$ is treated with NH_3, an analogous reaction occurs to generate $[LFe(\mu\text{-}NH_2)]_2$ (**5**) (Scheme S-2). Each of these compounds has been crystallographically verified (see below).

Treatment of **5** with 2 equiv of H_2O quantitatively generates NH_3 and the hydroxide dimer **6**. When **5** is treated with only 1 equiv of H_2O is used, ^{1}H NMR spectroscopy shows a mixture of **5**, **6**, and a new species (**M**). This new species is assigned as a diiron complex with bridging hydroxide and amide ligands $[LFe]_2(\mu\text{-}OH)(\mu\text{-}NH_2)$ (**M**), because the ligand-based peaks in its ^{1}H NMR spectrum lie between those of **5** and **6**. To support this hypothesis, equimolar amounts of **5** and **6** were mixed, which gave ligand exchange to form significant amounts of the mixed

hydroxide/amide **M** in solution (eq S-1). After 72 h, the reaction gives a statistical 1:2:1 mixture of **5**/**M**/**6**. Analogous ligand exchange reactivity is also observed with mixtures of **5** and [LFe(μ-Cl)]$_2$ to generate the mixed diiron chloride/amide compound. Both mixed ligand compounds contain only 5 resonances in their ^{1}H NMR spectra (Figures S-26 and S-27), consistent with averaged D_{2h} symmetric structures in solution that result from a rapid dynamic process. Attempts to isolate pure mixed-bridge compounds were unsuccessful because of these equilibria, precluding more detailed characterization. Nevertheless, it is interesting that the hydride ligand does not form stable mixed bridging ligand structures in the diiron framework, and that only amide, hydroxide, and chloride ligands are able to form stable mixed bridging ligand structures.

Scheme S-2. Amide, hydride, and hydroxide ligand exchange

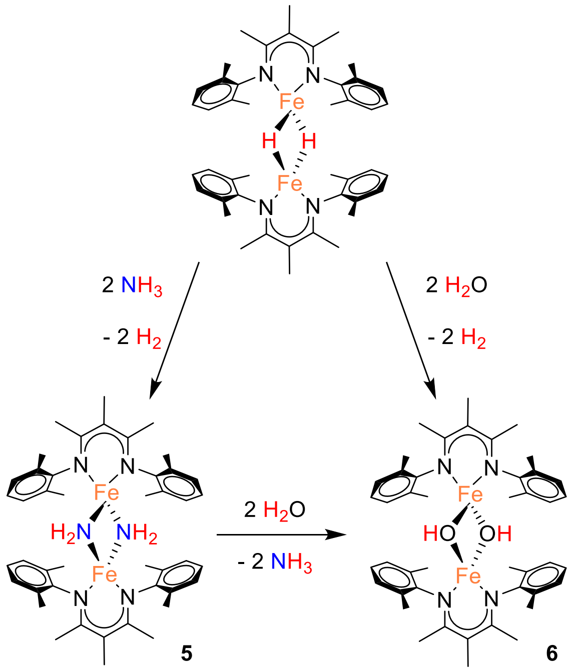

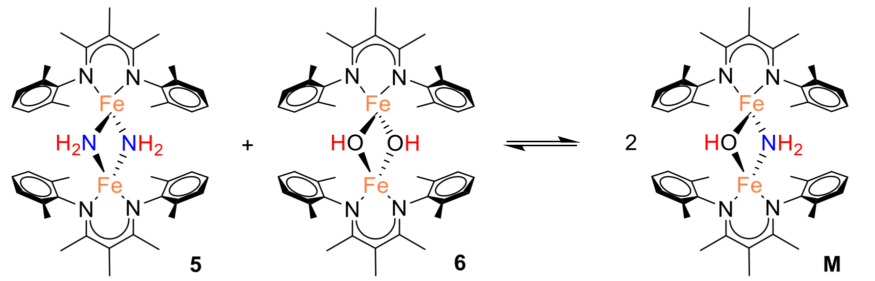

(eq S-1)

Definitive characterization of N_2-derived iron complexes with nitride, imide, amide and hydroxide ligands. One characteristic difference between the nitride, imide, and amide ligands is the Fe–N bond distances. The Fe–N bond distances to the μ_2-bridging nitride in **1** (1.809(2) and 1.812(2) Å) are more than 0.05 Å shorter than the Fe–NH bonds in **3** (1.866(5) and 1.874(5) Å), which in turn are more than 0.1 Å shorter than the Fe–NH_2 bonds in **4** and **5** (2.016(2) – 2.111(2) Å). While X-ray crystallography plays a critical role in the structural characterization of the compounds reported herein, there are limitations associated with the definitive assignment of the OH, N, NH, and NH_2 bridging ligands solely based on this characterization technique. For example, the Fe–O bond distances in **6** are only slightly shorter than the Fe–N bond distances in the bis(amide) **5**. Thus, it is important that IR corroborates the number and type of proton environments postulated to be on the bridging ligands from X-ray diffraction. Compound **6** has a diagnostic O–H stretch in its IR spectrum at 3665 cm^{-1}, and the amide and imide ligands of **3**, **4**, and **5** all have diagnostic N–H stretching bands in the range of 3300–3400 cm^{-1}.

Mössbauer spectroscopy is complementary, because it gives the number of unique Fe environments within the individual compounds as well as the assignment of oxidation states within those Fe environments.[12] In each case, the spectroscopically determined oxidation states balance the charges assigned to the bridging ligands (from X-ray and IR analysis). For instance, the Mössbauer spectrum of the previously reported tetrairon bis(nitride) **1** contains three signals in a 2:1:1 ratio signifying that the compound contains three unique Fe environments. The larger signal has a low isomer shift of δ = 0.29 mm/s that indicates high-spin iron(III) sites and is assigned to the two equivalent bridging Fe, and the higher isomer shifts of δ = 0.68 mm/s and δ = 0.96 mm/s indicate high-spin iron(II), which are assigned to the three-coordinate Fe^{2+} site and the pendant Fe^{2+} site, respectively (based on the lower isomer shift for lower coordination number and higher isomer shift

for more ionic ligands, as well as on similarity to literature precedents).[12] Using these values as a guide, the Mössbauer spectrum of the triiron nitride/imide **3** contains two signals in a 2:1 ratio with δ = 0.29 mm/s and 0.61 mm/s, respectively, which indicates that the two bridging Fe sites are high-spin iron(III) and the three-coordinate site is high-spin iron(II). Conversely, the Mössbauer spectrum of the triiron nitride/amide **4** contains three signals, despite having a very similar structure to the precursor **3**. The reason for **4** having three unique Fe environments becomes evident upon assignment of oxidation states by analysis of the isomer shift values from the Mössbauer spectrum. Assignment of the three-coordinate Fe site with δ = 0.65 mm/s comes from comparison to the analogous Fe sites in **1** and **3**. The remaining two Fe sites that bridge the N atoms in **4** now have different Fe environments as evidenced by their δ values of 0.39 mm/s (indicating high-spin iron(III)) and 0.72 mm/s (indicating high-spin iron(II)). Note that the latter has a very similar isomer shift to the diiron(II) bis(amide) **5** (δ = 0.75 mm/s). The combination of structural and spectroscopic characterization of these compounds therefore allows the unambiguous assignment of nitride, imide, amide, and hydroxide ligands in compounds **3**, **4**, **5**, and **6**.

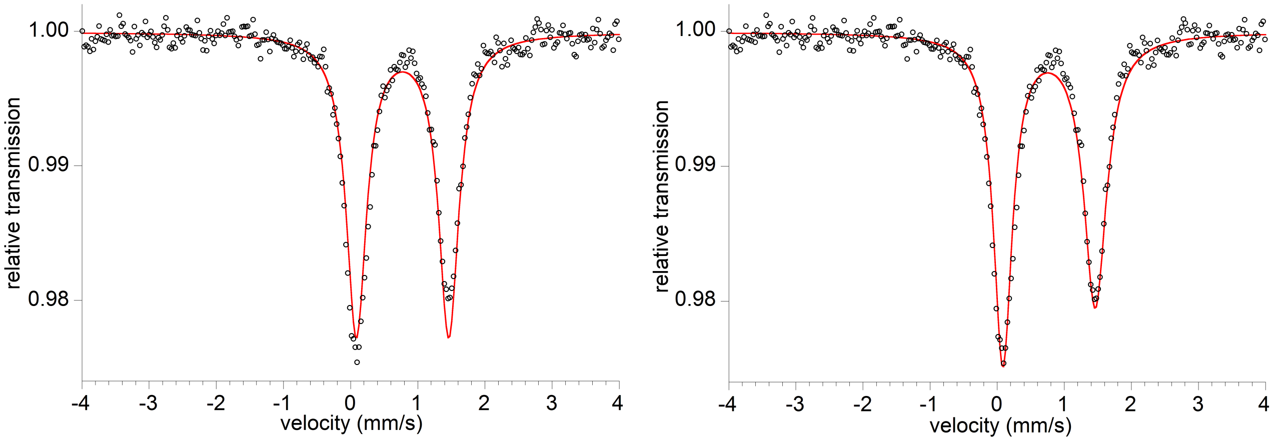

Figure S-1. Zero-field Mössbauer spectrum of $LFe(OC_6H_2{}^tBu_3)$ (**2**) recorded at 173 K. The black circles are the data and the red line represents a simulation of the spectrum with δ = 0.77 mm/s and $|\Delta E_Q|$ = 1.38 mm/s. Simulations with symmetric (left: γ = 0.36) and asymmetric (right: γ_L = 0.34, γ_R = 0.41) line width parameters.

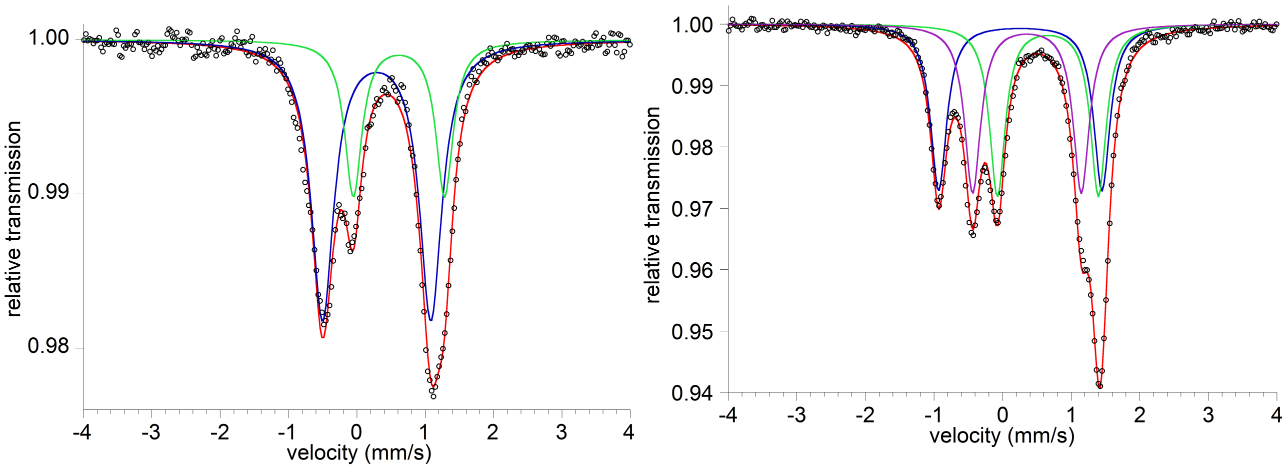

Figure S-2. Zero-field Mössbauer spectra of $[LFe]_2(\mu_2\text{-}NH)(\mu_3\text{-}N)[FeL]$ (**3**) recorded at 173 K (left) and 80 K (right). The black circles are the data and the red line represents the sum of a two component (left) or three component (right) simulation of the spectrum. At 173 K, compound **3** contains two unique iron environments in a 2:1 ratio shown in blue (δ = 0.29 mm/s, $|\Delta E_Q|$ = 1.58 mm/s, γ = 0.40) and green (δ = 0.61 mm/s, $|\Delta E_Q|$ = 1.34 mm/s, γ = 0.31), respectively. At 80 K,

compound **3** contains three unique iron environments in a 1:1:1 ratio shown in blue (δ = 0.26 mm/s, $|\Delta E_Q|$ = 2.38 mm/s, γ = 0.29), purple (δ = 0.35 mm/s, $|\Delta E_Q|$ = 1.58 mm/s, γ = 0.28), and green (δ = 0.66 mm/s, $|\Delta E_Q|$ = 1.47 mm/s, γ = 0.28).

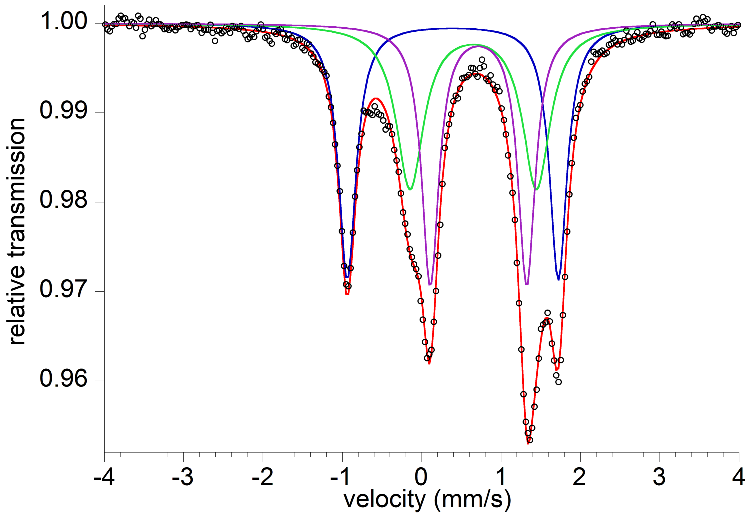

Figure S-3. Zero-field Mössbauer spectrum of $[LFe]_2(\mu_2\text{-}NH_2)(\mu_3\text{-}N)[FeL]$ (**4**) recorded at 80 K. The black circles are the data and the red line represents the sum of a three component simulation of the spectrum. Compound **4** contains three unique iron environments in a 1:1:1 ratio shown in blue (δ = 0.39 mm/s, $|\Delta E_Q|$ = 2.66 mm/s, γ = 0.27), green (δ = 0.65 mm/s, $|\Delta E_Q|$ = 1.59 mm/s, γ = 0.42), and purple (δ = 0.72 mm/s, $|\Delta E_Q|$ = 1.22 mm/s, γ = 0.26).

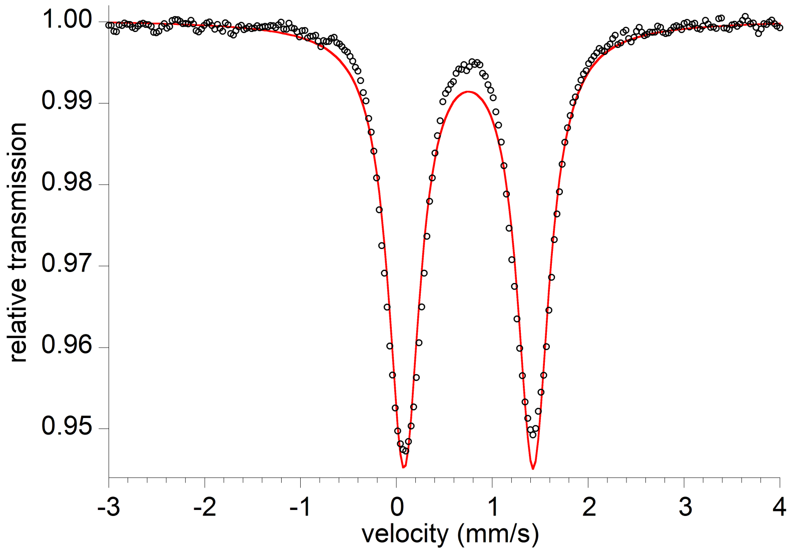

Figure S-4. Zero-field Mössbauer spectrum of [LFe(μ-NH_2)]$_2$ (**5**) recorded at 80 K. The black circles are the data and the red line represents a simulation of the spectrum with δ = 0.75 mm/s, $|\Delta E_Q|$ = 1.35 mm/s, γ = 0.40.

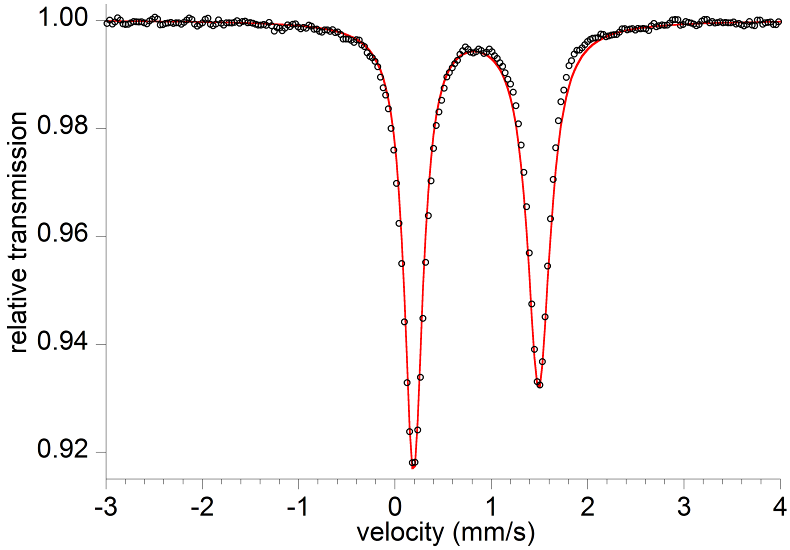

Figure S-5. Zero-field Mössbauer spectrum of [LFe(μ-OH)]$_2$ (**6**) recorded at 80 K. The black circles are the data and the red line represents a simulation of the spectrum with δ = 0.84 mm/s, $|\Delta E_Q|$ = 1.30 mm/s, γ_L = 0.24, γ_R = 0.29.

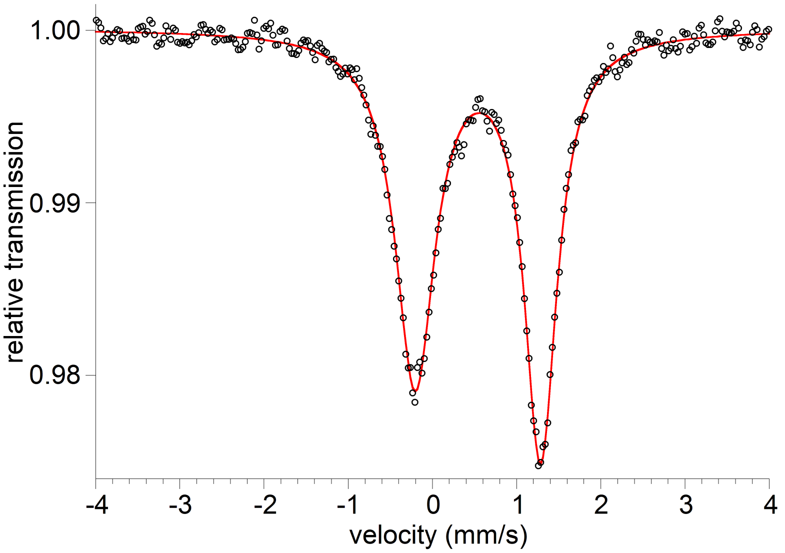

Figure S-6. Zero-field Mössbauer spectrum of [LFe(μ-C≡CPh)]$_2$ (**7a**) recorded at 173 K. The black circles are the data and the red line represents a simulation of the spectrum with δ = 0.54 mm/s, $|\Delta E_Q|$ = 1.49 mm/s, γ_L = 0.57, γ_R = 0.47.

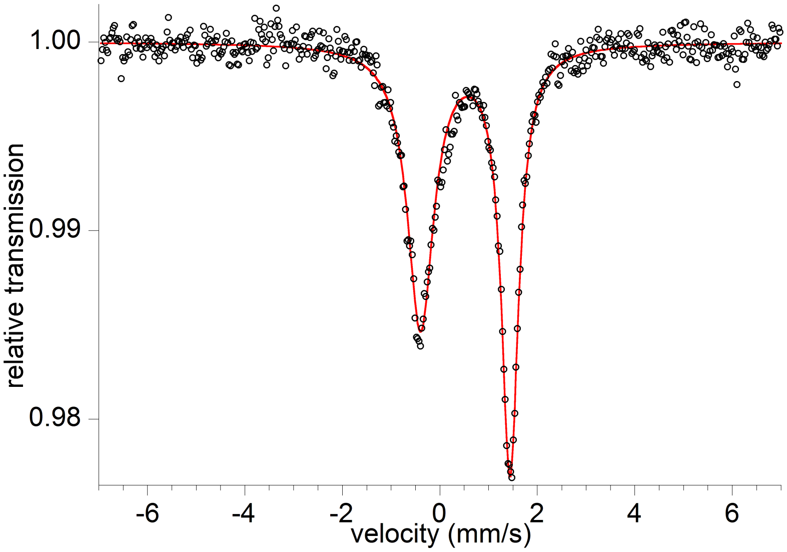

Figure S-7. Zero-field Mössbauer spectrum of the crude solid [LFe{μ-C≡C$C_6H_2(CF_3)_2$}]$_2$ (**7b**) recorded at 173 K. The black circles are the data and the red line represents a simulation of the spectrum with δ = 0.52 mm/s, $|\Delta E_Q|$ = 1.84 mm/s, γ_L = 0.66, γ_R = 0.43.

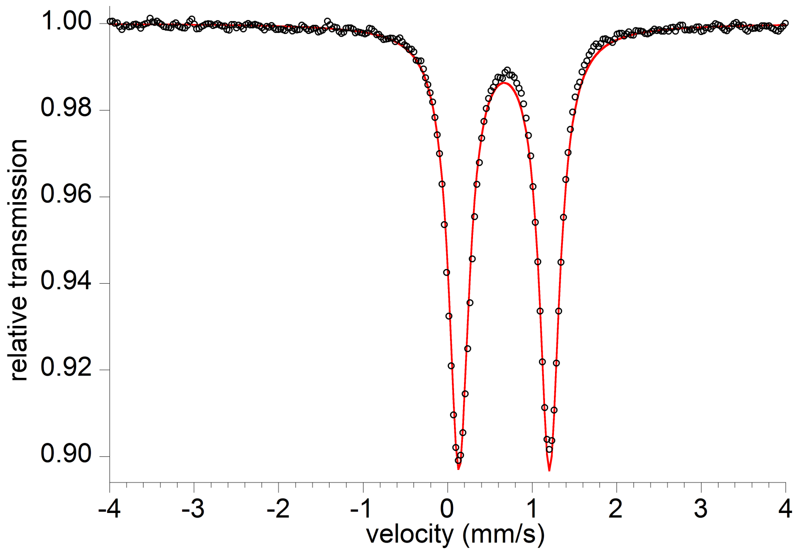

Figure S-8. Zero-field Mössbauer spectrum of LFe(η_5-C_9H_7) (**8**) recorded at 80 K. The black circles are the data and the red line represents a simulation of the spectrum with δ = 0.67 mm/s, $|\Delta E_Q|$ = 1.07 mm/s, γ = 0.29.

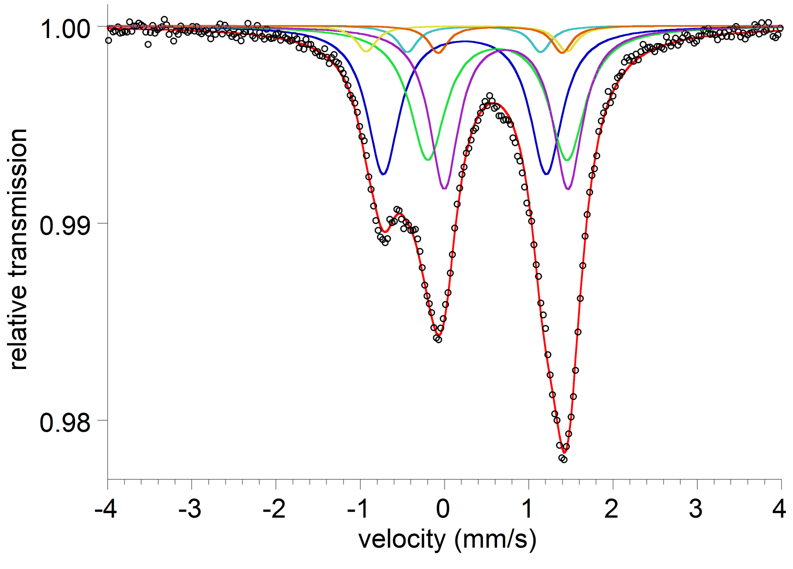

Figure S-9. Zero-field Mössbauer spectrum of in situ generated K[{LFe}$_2$(μ_2-NH)(μ_3-N){FeL}] (**R**) recorded at 80 K. The black circles are the data and the red line represents the sum of a six component simulation of the spectrum. Compound **R** contains three unique iron environments in a 1:1:1 ratio shown in blue (δ = 0.24 mm/s, $|\Delta E_Q|$ = 1.94 mm/s, γ = 0.46), green (δ = 0.63 mm/s, $|\Delta E_Q|$

= 1.65 mm/s, γ = 0.52), and purple (δ = 0.73 mm/s, $|\Delta E_Q|$ = 1.47 mm/s, γ = 0.42). The sample also contains 10% unreacted **2** shown in yellow (δ = 0.26 mm/s, $|\Delta E_Q|$ = 2.38 mm/s, γ = 0.29), cyan (δ = 0.35 mm/s, $|\Delta E_Q|$ = 1.58 mm/s, γ = 0.28), and orange (δ = 0.66 mm/s, $|\Delta E_Q|$ = 1.47 mm/s, γ = 0.28).

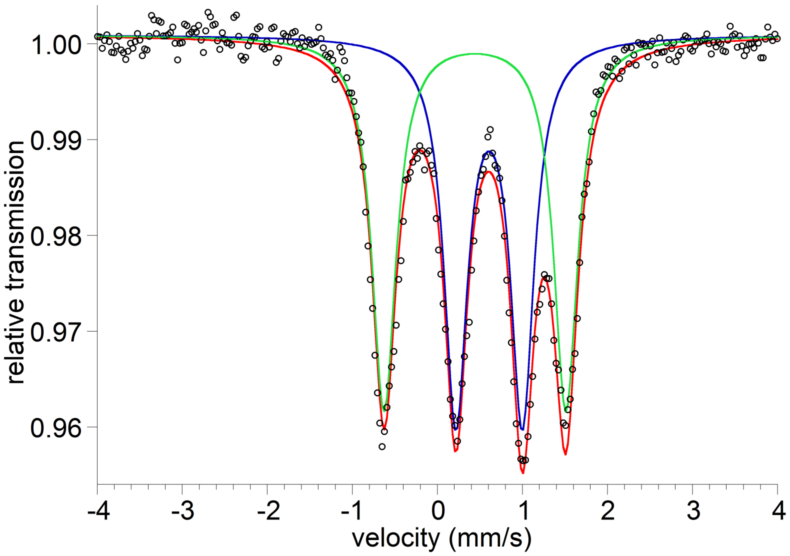

Figure S-10. Zero-field Mössbauer spectrum of $[LFe(\mu\text{-}H)]_2$ recorded at 80 K. The black circles are the data and the red line represents the sum of a two component simulation of the spectrum. $[LFe(\mu\text{-}H)]_2$ contains two unique iron environments in a 1:1 ratio shown in blue (δ = 0.61 mm/s, $|\Delta E_Q|$ = 0.78 mm/s, γ = 0.33) and green (δ = 0.44 mm/s, $|\Delta E_Q|$ = 2.14 mm/s, γ = 0.34).

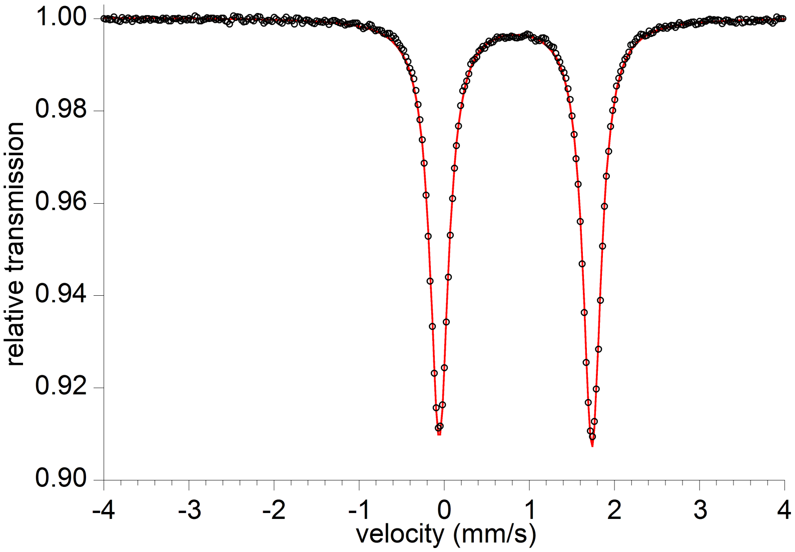

Figure S-11. Zero-field Mössbauer spectrum of L_2Fe recorded at 80 K. The black circles are the data and the red line represents a simulation of the spectrum with $\delta = 0.84$ mm/s, $|\Delta E_Q| = 1.80$ mm/s, $\gamma = 0.26$.

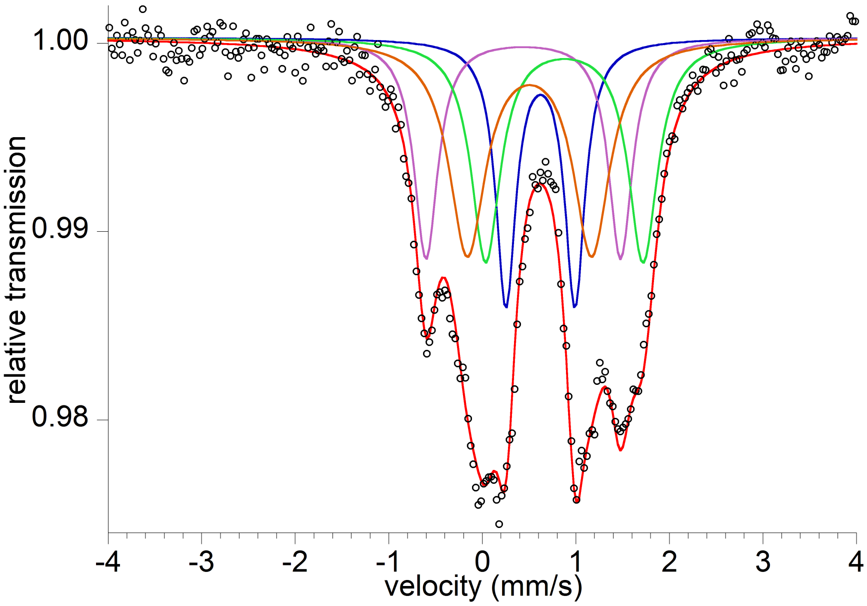

Figure S-12. Zero-field Mössbauer spectrum of the filtered reaction mixture of **1** with H_2 (1 atm) recorded at 80 K. The black circles are the data and the red line represents the sum of a four component simulation of the spectrum. The L_2Fe component (26% of the signal) is shown in green

(δ = 0.88 mm/s, $|\Delta E_Q|$ = 1.68 mm/s, γ = 0.38). The [LFe(μ-H)]$_2$ component (43% of the signal) is shown in blue (δ = 0.62 mm/s, $|\Delta E_Q|$ = 0.74 mm/s, γ = 0.26) and purple (δ = 0.44 mm/s, $|\Delta E_Q|$ = 2.08 mm/s, γ = 0.31). The ^{1}H NMR silent species (31% of the signal) is shown in orange (δ = 0.50 mm/s, $|\Delta E_Q|$ = 1.33 mm/s, γ = 0.47).

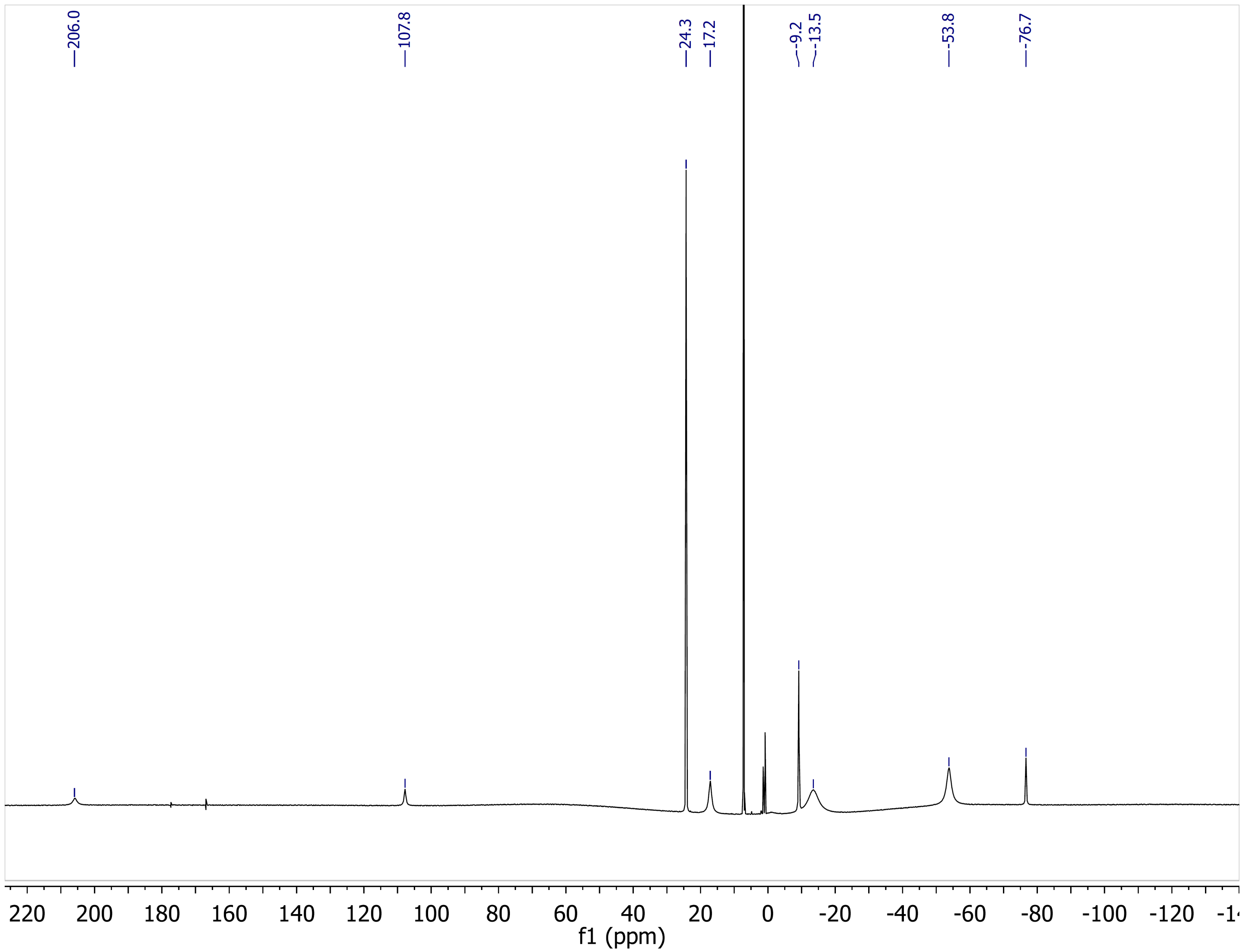

Figure S-13. ^{1}H NMR spectrum of LFe(OC$_6$H$_2$^{t}Bu$_3$) (**2**) in C_6D_6.

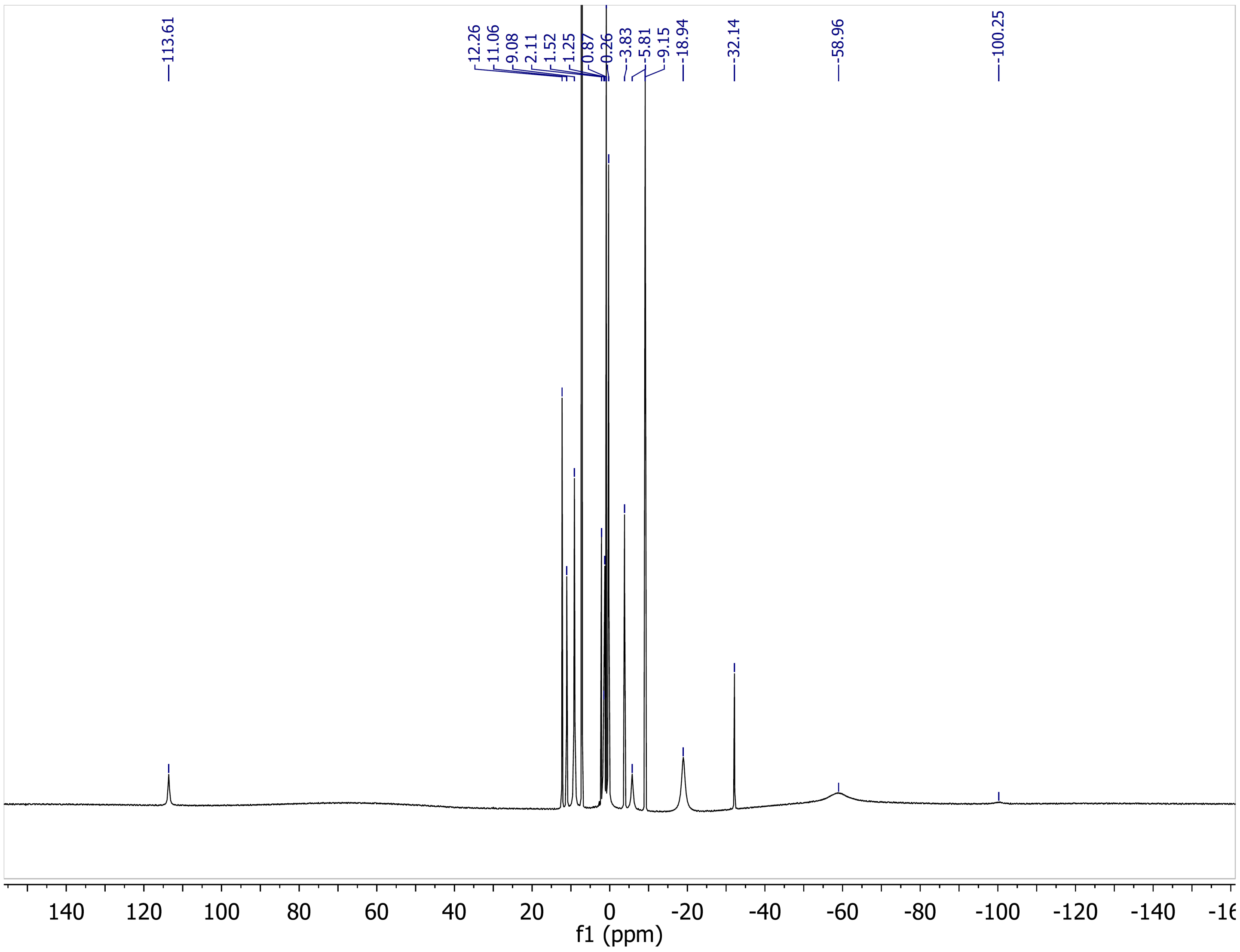

Figure S-14. ^{1}H NMR spectrum of $[LFe]_2(\mu_2\text{-NH})(\mu_3\text{-N})[FeL]$ (**3**) in C_6D_6.

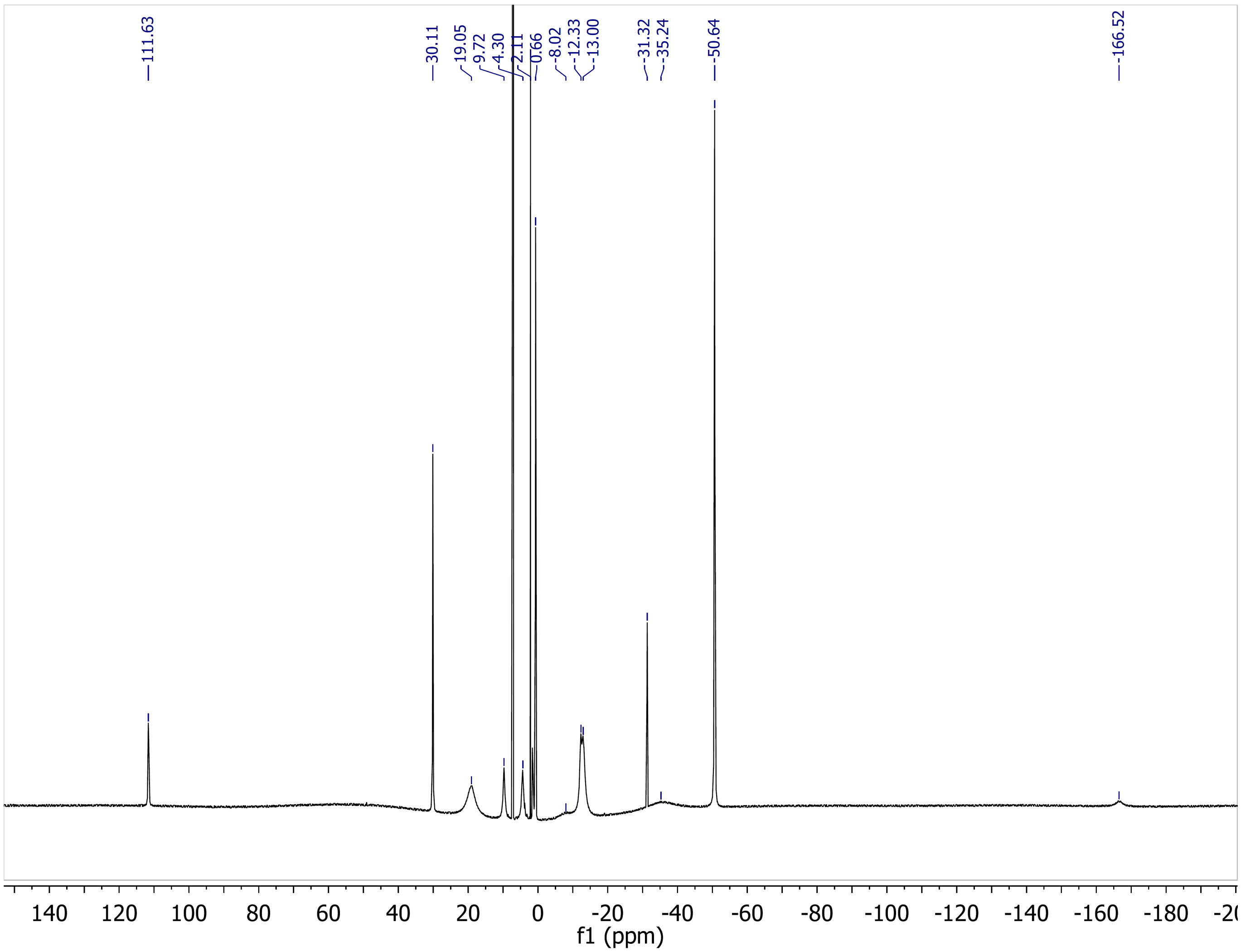

Figure S-15. ^{1}H NMR spectrum of $[LFe]_2(\mu_2\text{-}NH_2)(\mu_3\text{-}N)[FeL]$ (**4**) in C_6D_6 with Cp_2Co internal capillary standard (δ -51 ppm).

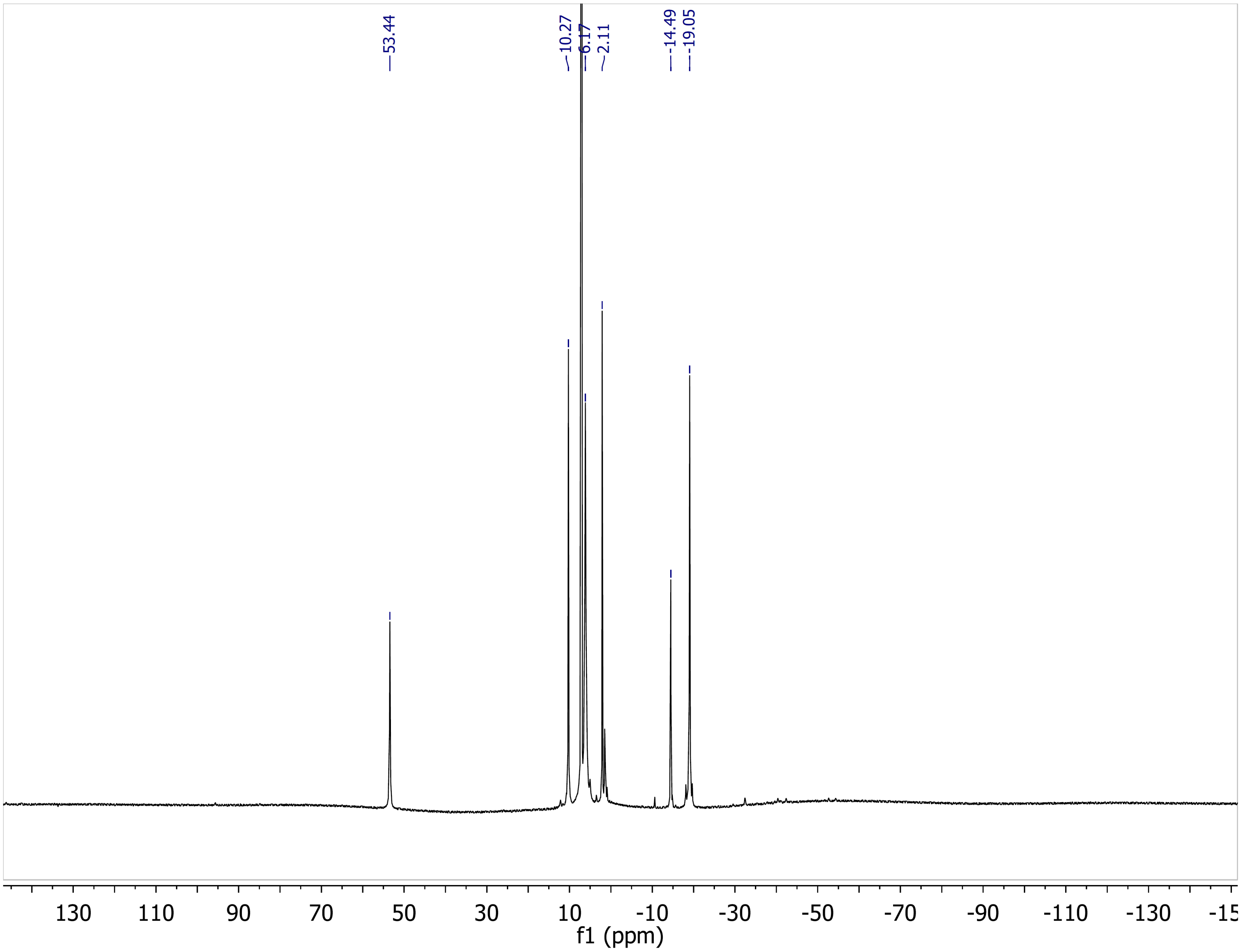

Figure S-16. ^{1}H NMR spectrum of $[LFe(\mu\text{-}NH_2)]_2$ (**5**) in C_6D_6.

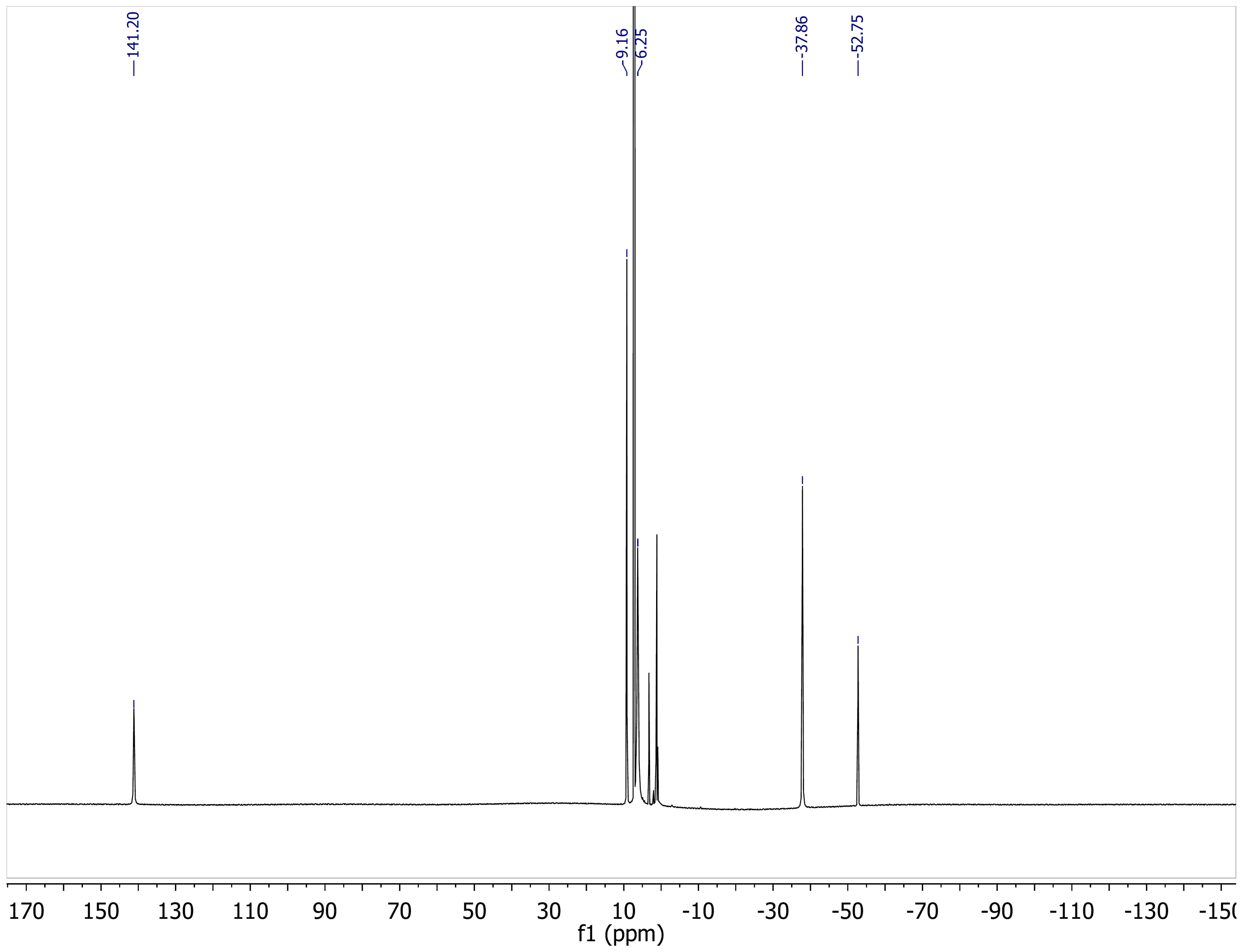

Figure S-17. ^{1}H NMR spectrum of $[LFe(\mu\text{-}OH)]_2$ (**6**) in C_6D_6.

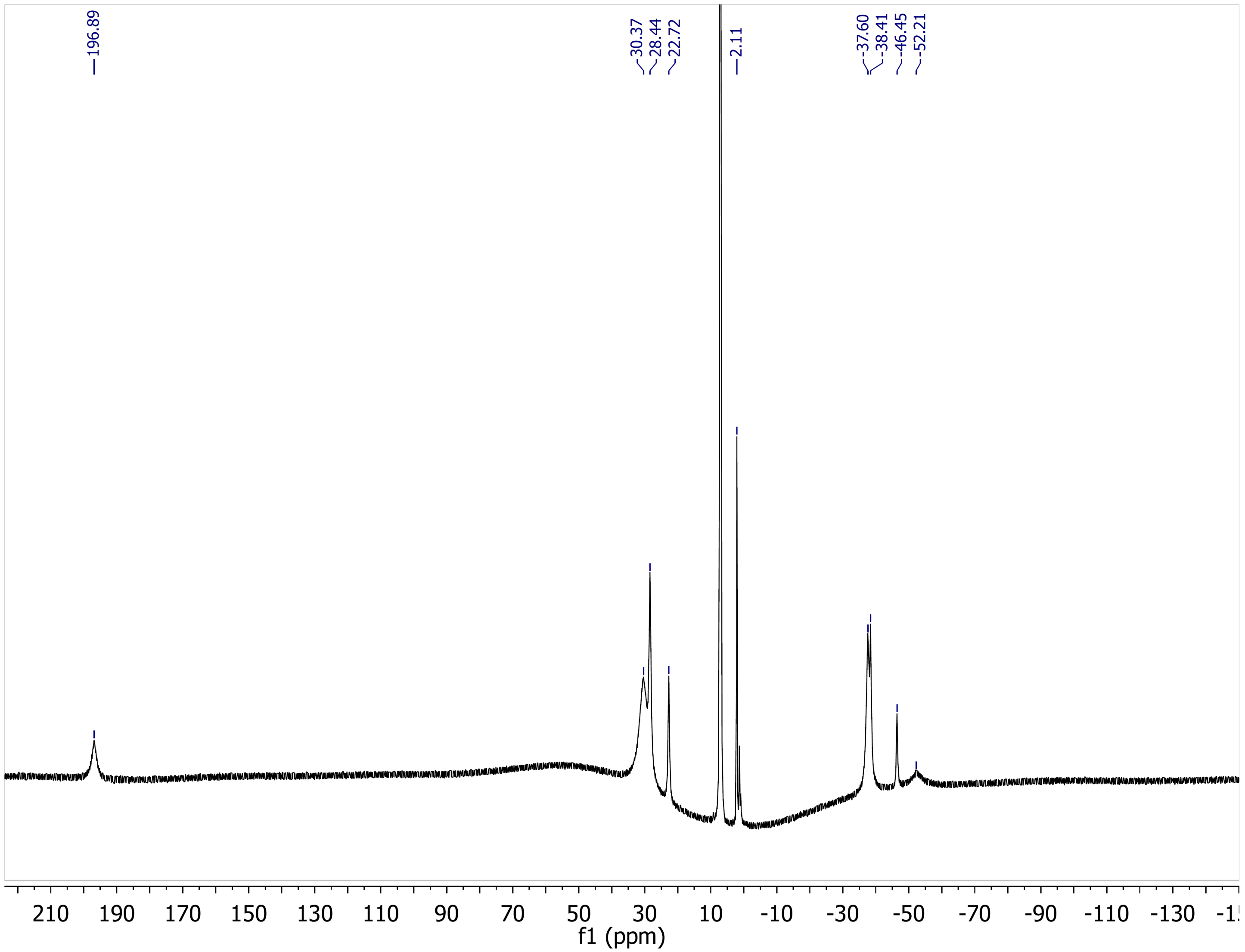

Figure S-18. ^{1}H NMR spectrum of [LFe(μ-C≡CPh]$_2$ (**7a**) in C_6D_6.

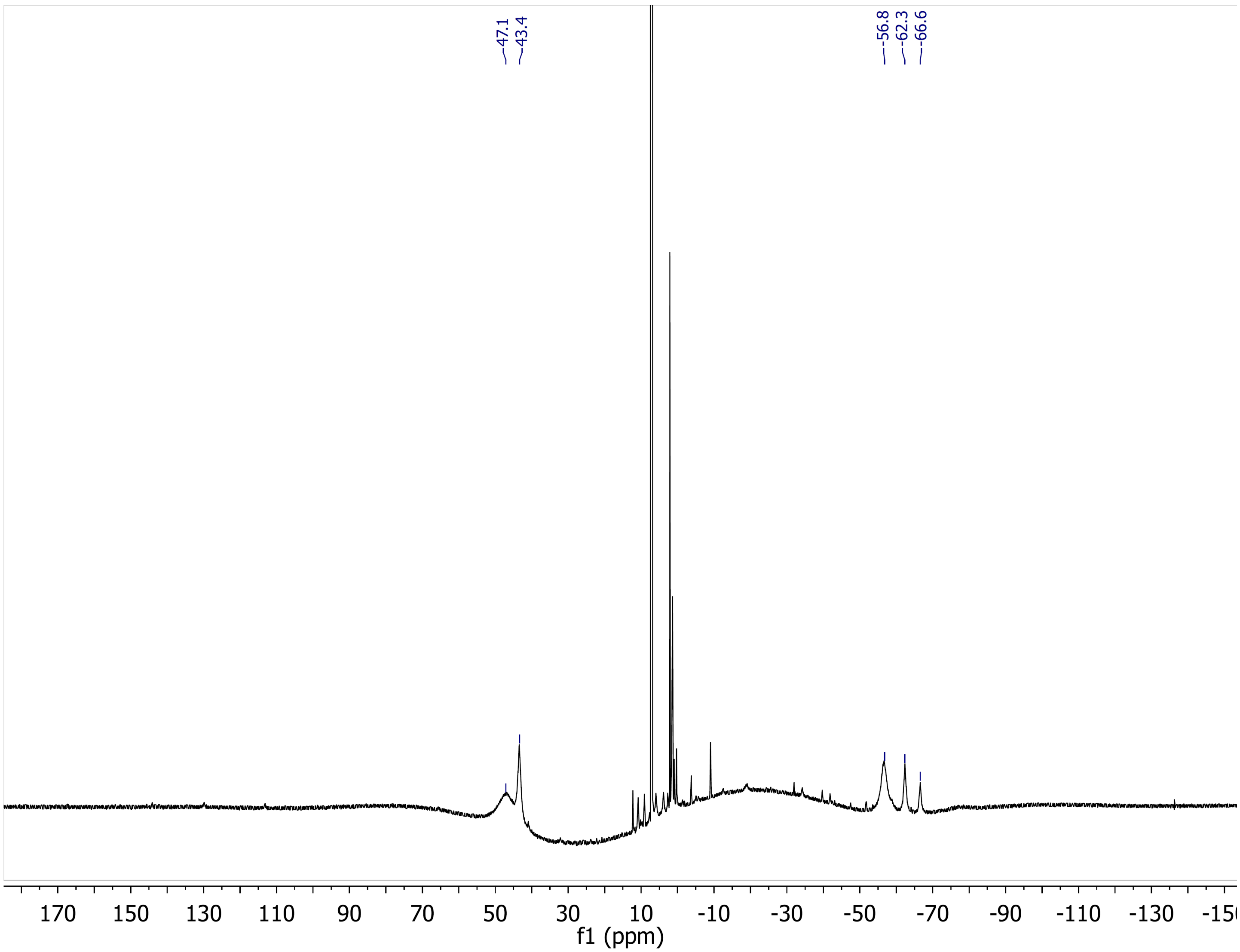

Figure S-19. ^{1}H NMR spectrum of the crude solid [LFe{μ-C≡$CC_6H_2(CF_3)_2$}]$_2$ (**7b**) in C_6D_6.

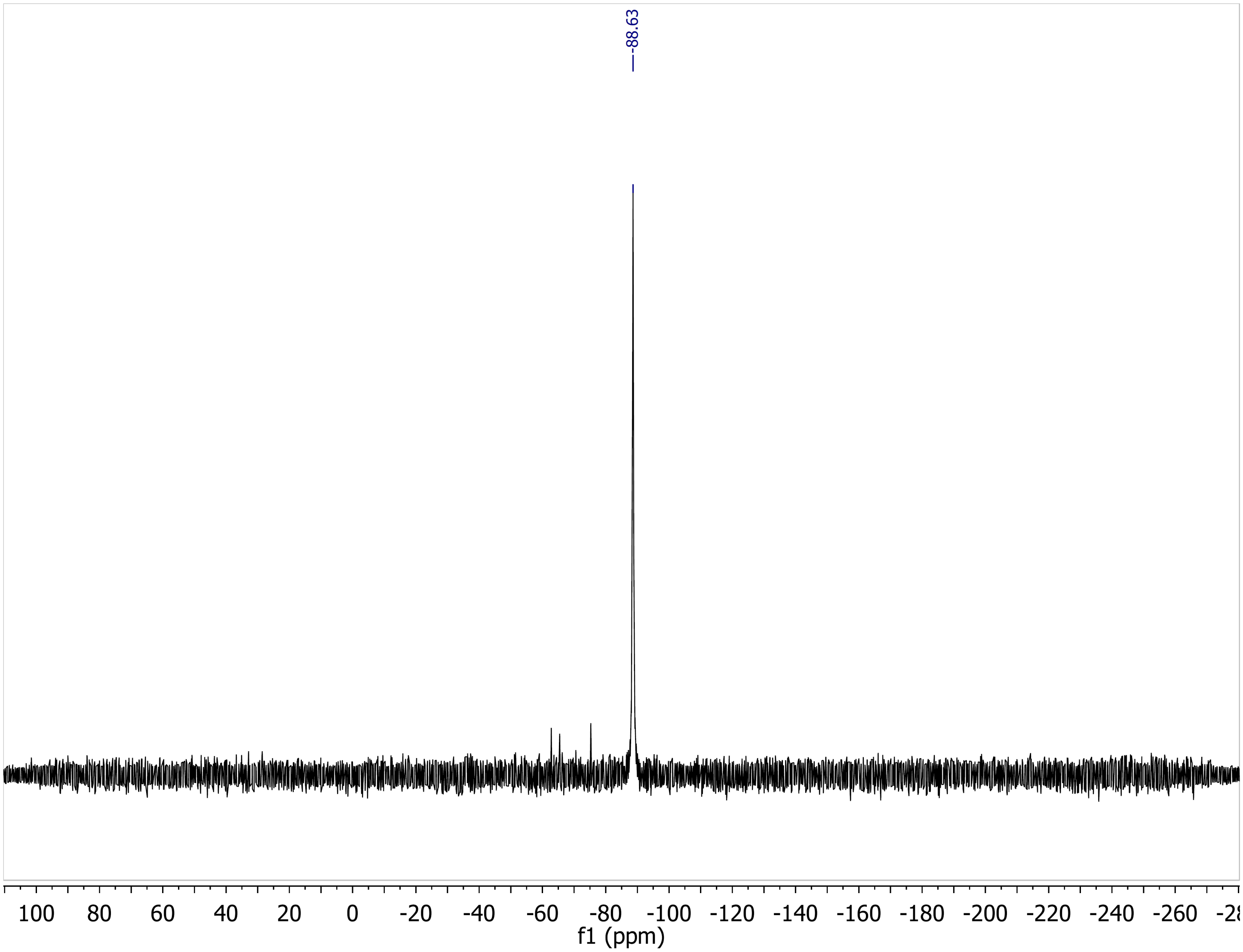

Figure S-20. ^{19}F NMR spectrum of the crude solid $[LFe\{\mu\text{-}C{\equiv}CC_6H_2(CF_3)_2\}]_2$ (**7b**) in C_6D_6.

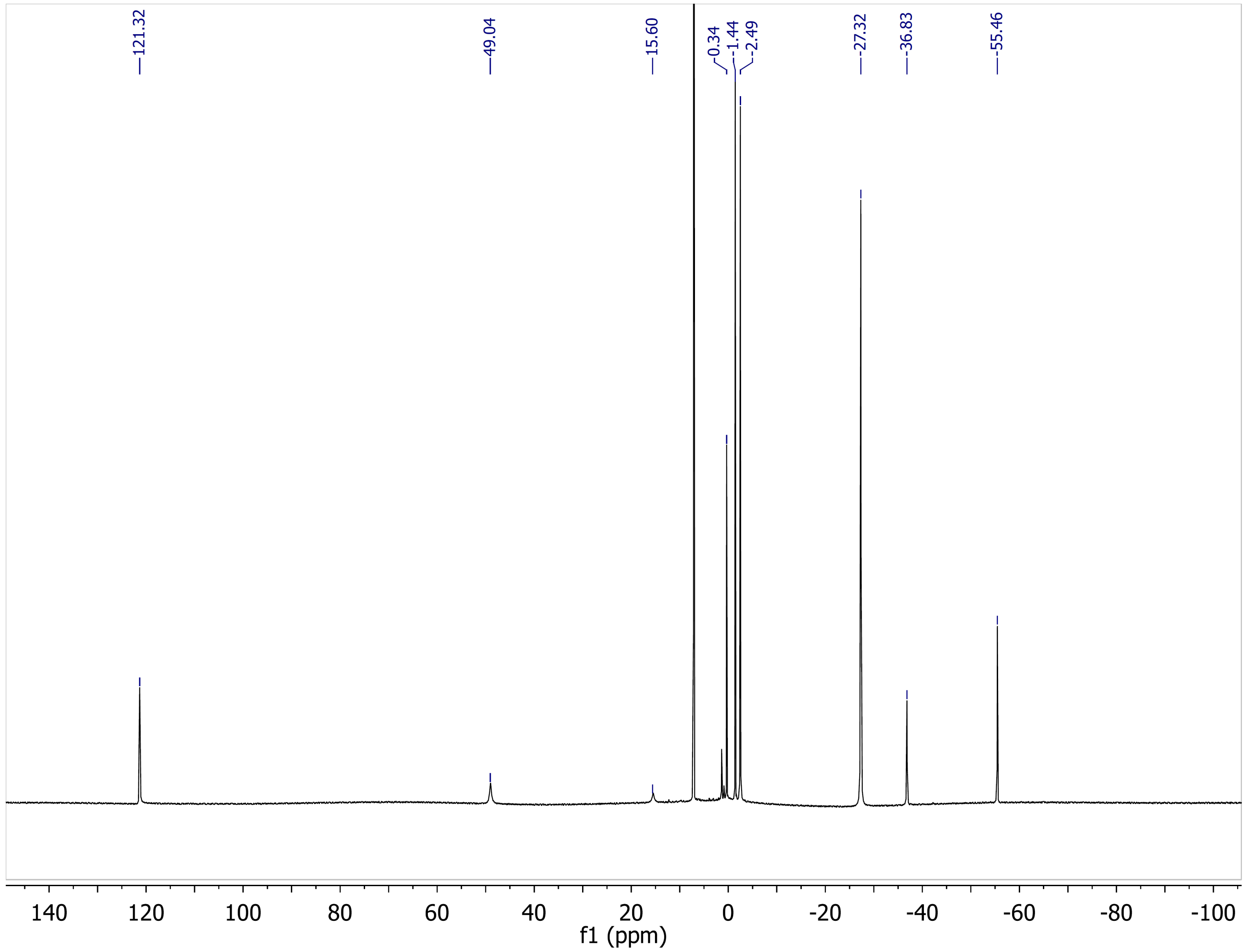

Figure S-21. ^{1}H NMR spectrum of LFe(η5-C_9H_7) (**8**) in C_6D_6.

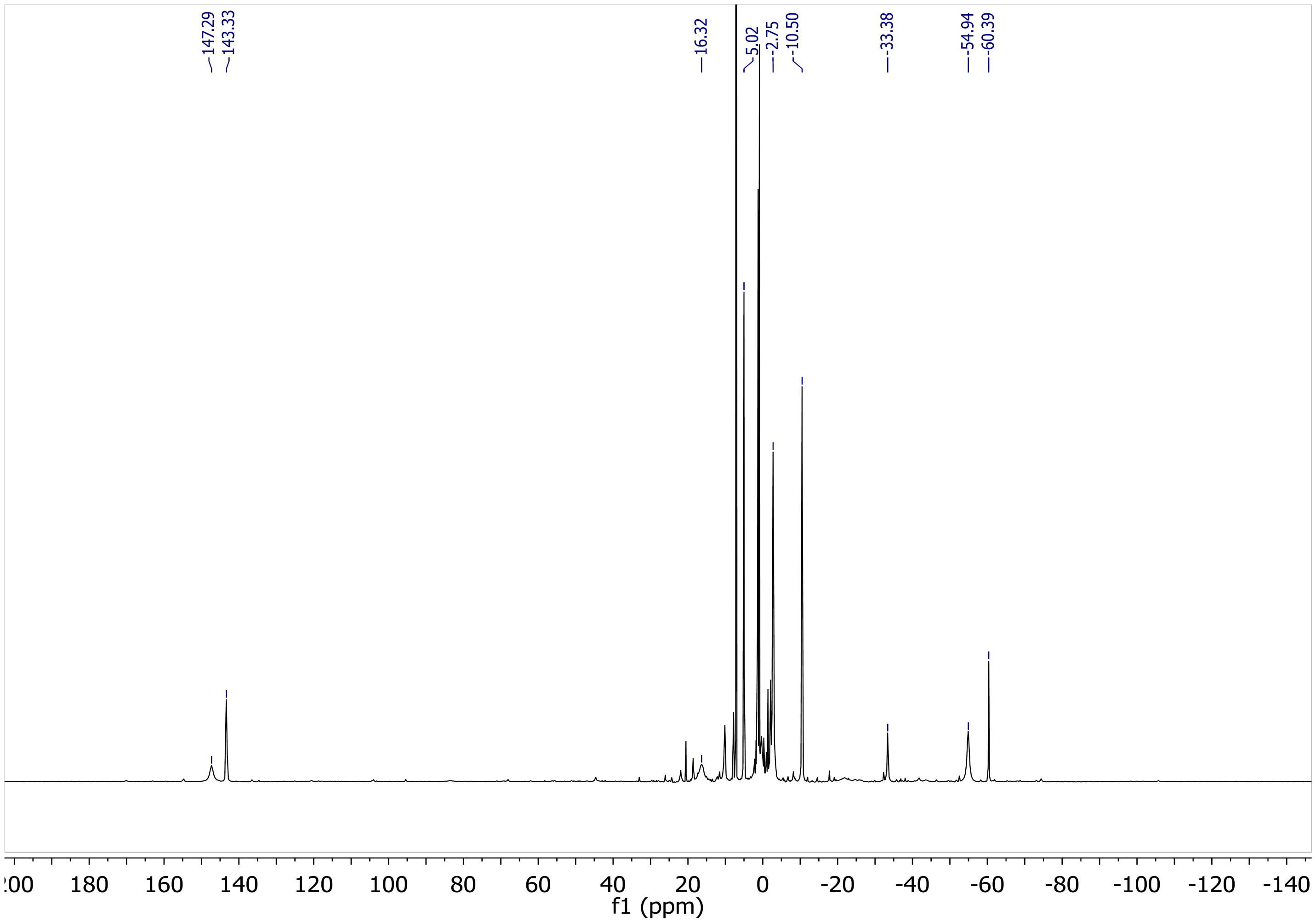

Figure S-22. ^{1}H NMR spectrum of the filtered reaction mixture of **1** with H_2 (1 atm) in C_6D_6.

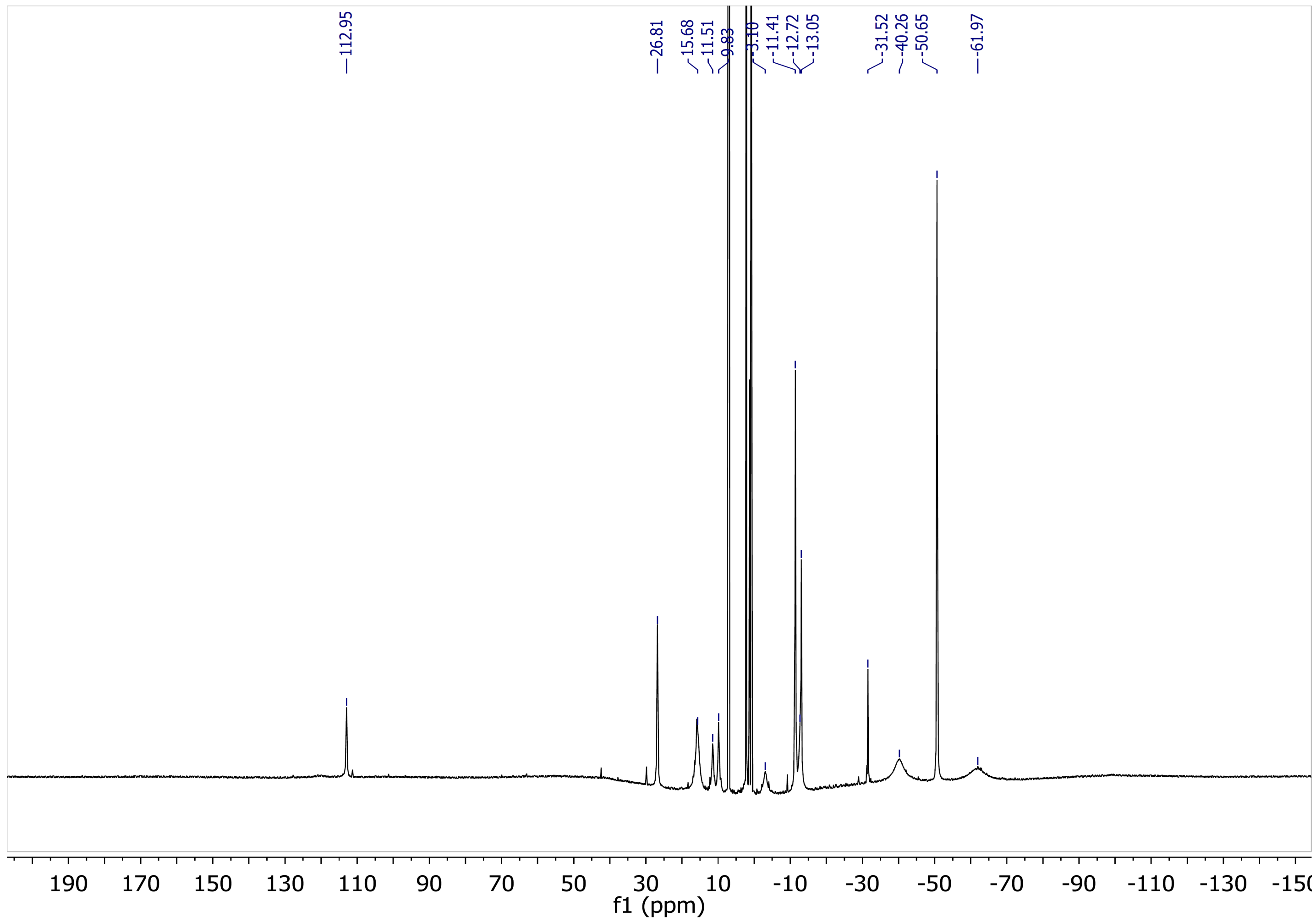

Figure S-23. ^{1}H NMR spectrum of in situ generated intermediate K[{LFe}$_2$(μ_2-NH)(μ_3-N){FeL}] (**R**) in C_7D_8 with Cp_2Co internal capillary standard (δ -51 ppm).

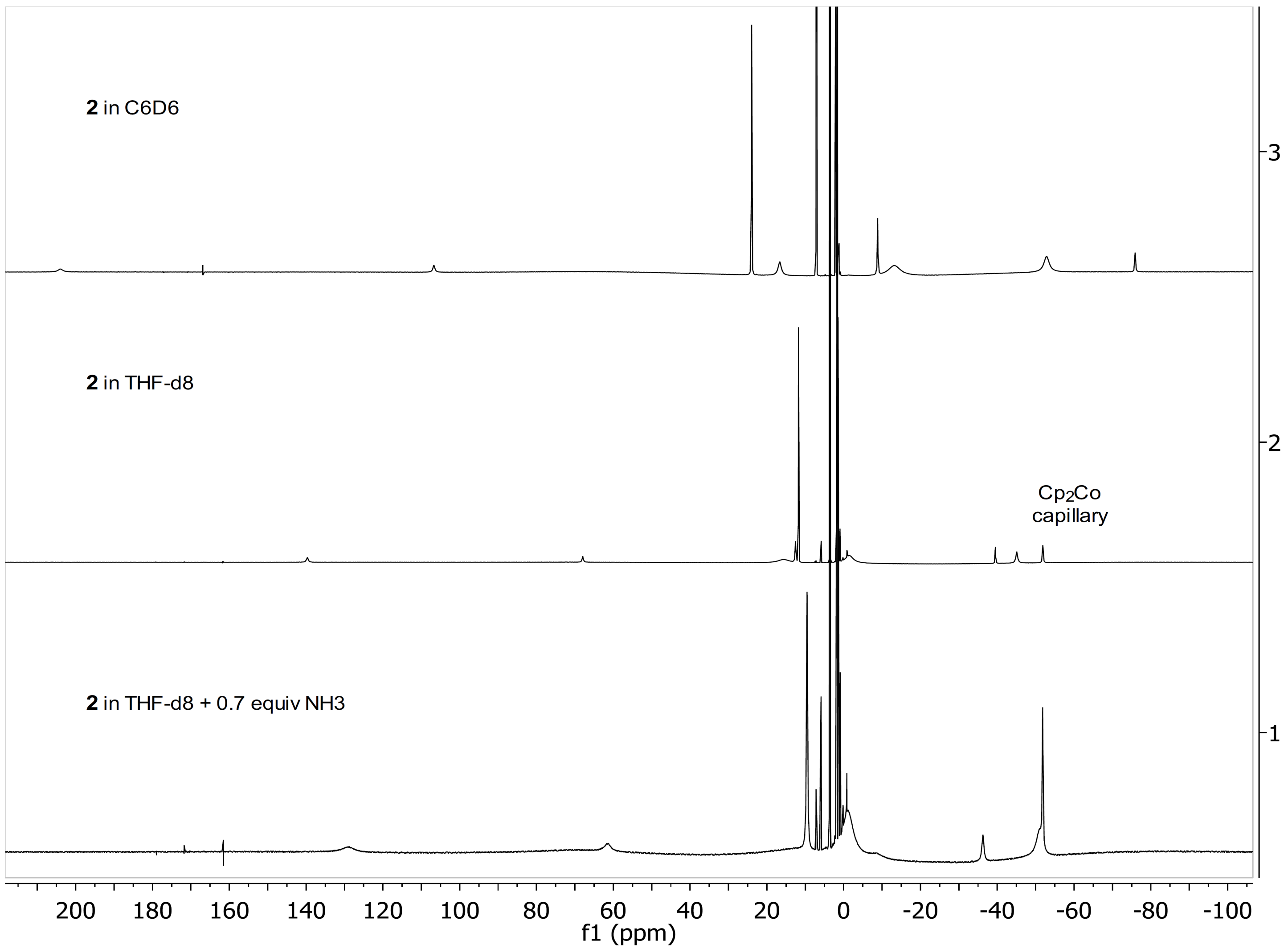

Figure S-24. ^{1}H NMR spectra of LFe(OC$_6$H$_2{}^t$Bu$_3$) (**2**) in C_6D_6 (top), in THF-d_8 (middle), and with 0.7 equiv NH_3 in THF-d_8 (bottom). The middle and bottom spectra contain a Cp_2Co internal capillary standard (δ -51 ppm).

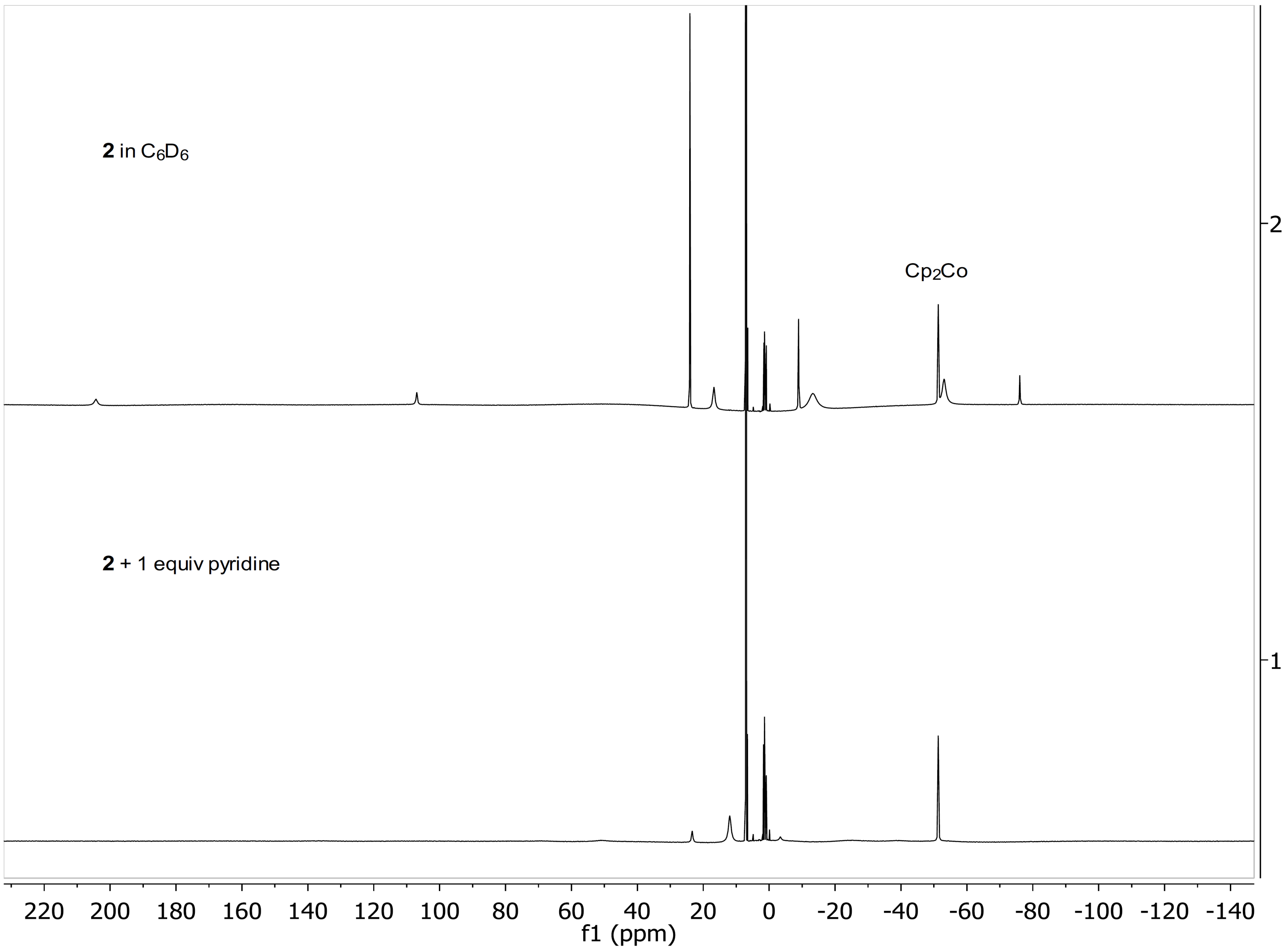

Figure S-25. ^{1}H NMR spectra of $LFe(OC_6H_2{}^tBu_3)$ (**2**) in C_6D_6 (top) and with 1 equiv pyridine (bottom) with Cp_2Co internal capillary standard (δ -51 ppm).

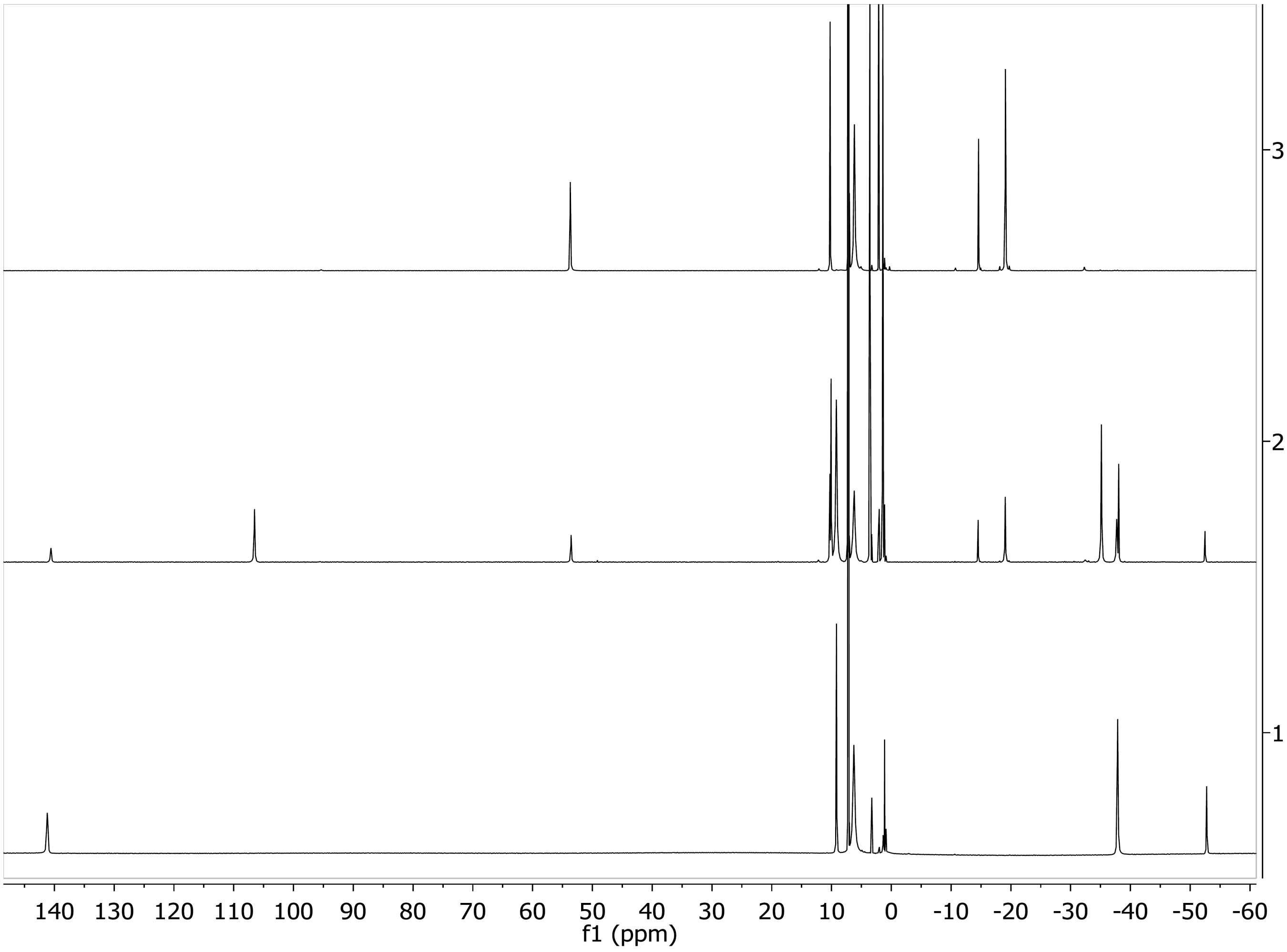

Figure S-26. ^{1}H NMR spectra in C_6D_6 of $[LFe(\mu\text{-}NH_2)]_2$ (**5**) (top), $[LFe(\mu\text{-}OH)]_2$ (**6**) (bottom), and an in situ generated 1:2:1 mixture of **5**/**M**/**6**, where **M** = $[LFe]_2(\mu\text{-}OH)(\mu\text{-}NH_2)$ (middle).

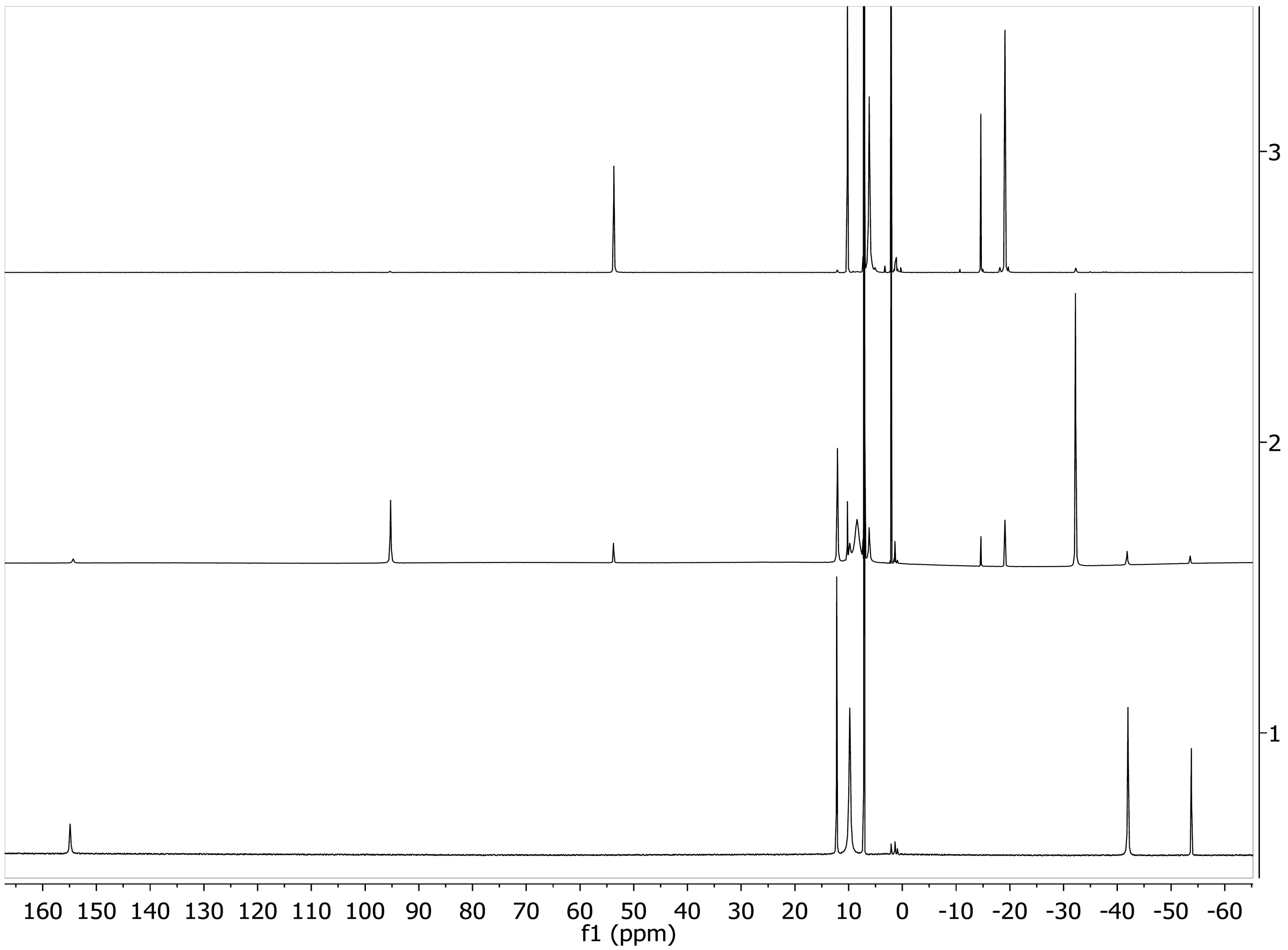

Figure S-27. ^{1}H NMR spectra in C_6D_6 of [LFe(μ-NH_2)]$_2$ (**5**) (top), [LFe(μ-Cl)]$_2$ (bottom), and an in situ generated 1:5:1 mixture of **5**/[LFe]$_2$(μ-Cl)(μ-NH_2)/[LFe(μ-Cl)]$_2$ (middle).

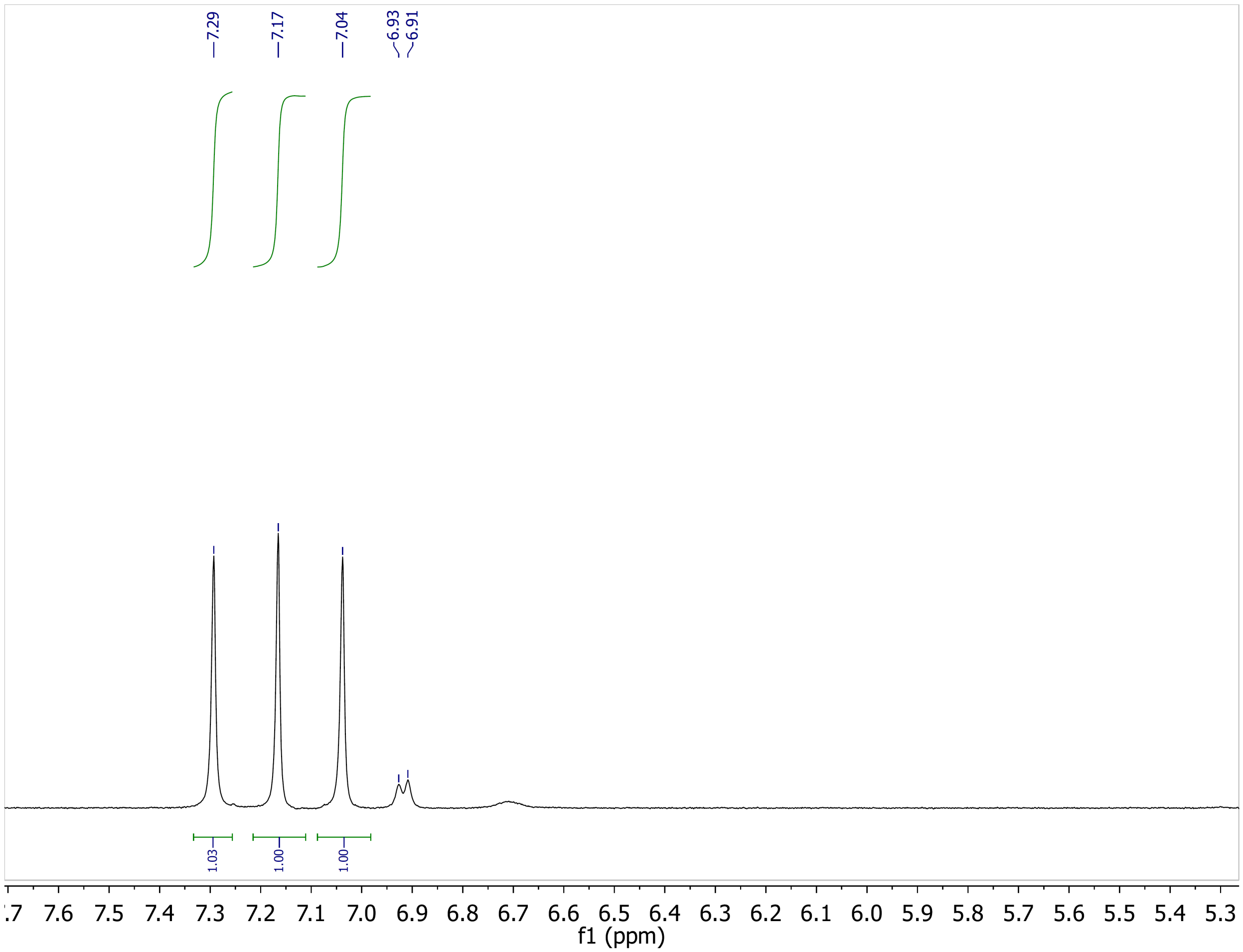

Figure S-28. ^{1}H NMR spectrum in DMSO-d_6 of $^{14}NH_4Cl$ derived from **1** + H_2SO_4, showing the triplet from coupling of ^{1}H and ^{14}N ($I = 1$).

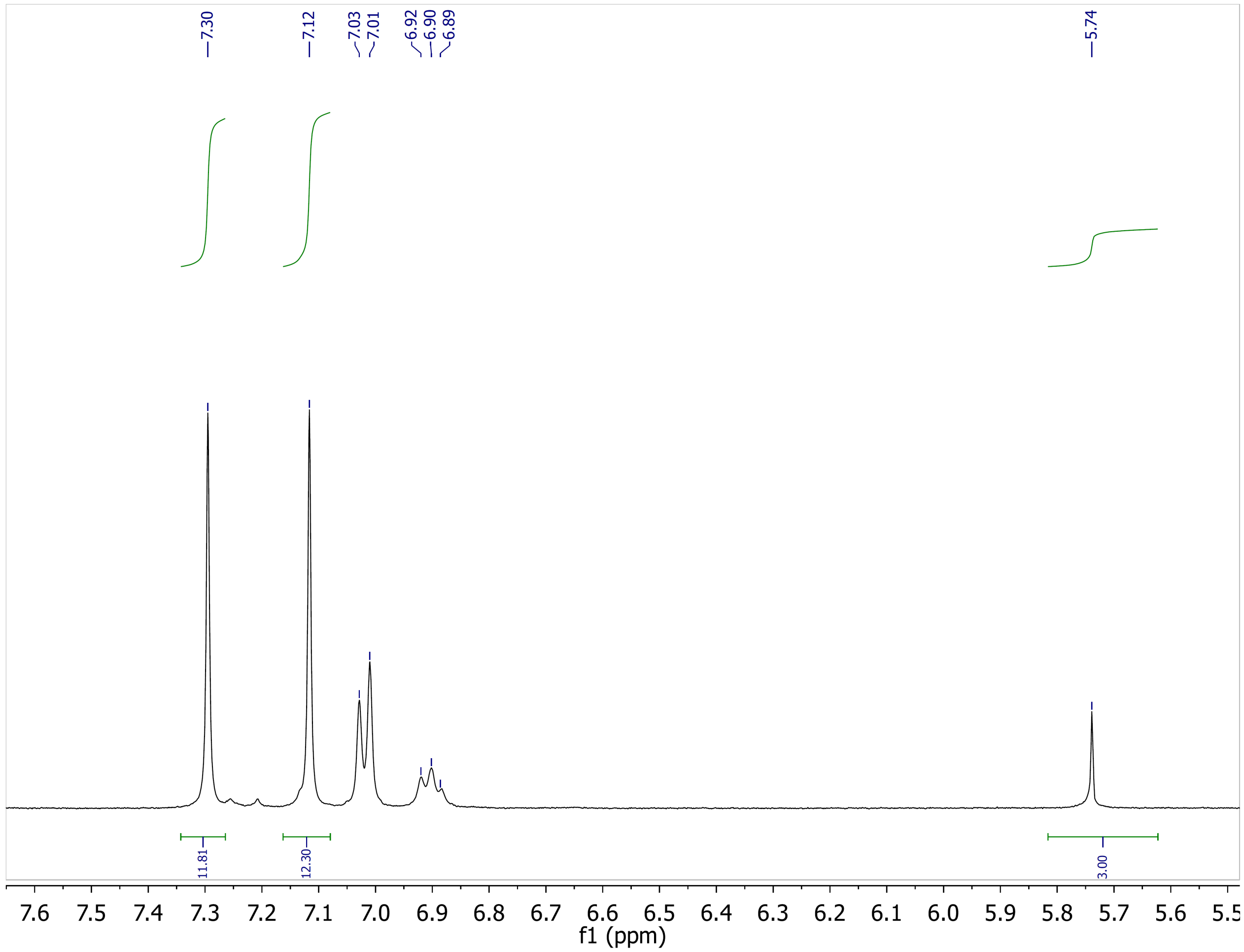

Figure S-29. ^{1}H NMR spectrum in DMSO-d_6 of $^{15}NH_4Cl$ derived from **1-^{15}N$_2$** + H_2SO_4, showing the triplet from coupling of ^{1}H and ^{15}N (I = 1/2). The singlet at 5.74 ppm is from 1,3,5-trimethoxybenzene (6.11 mM in C_6D_{12}) used as an internal standard for quantification.

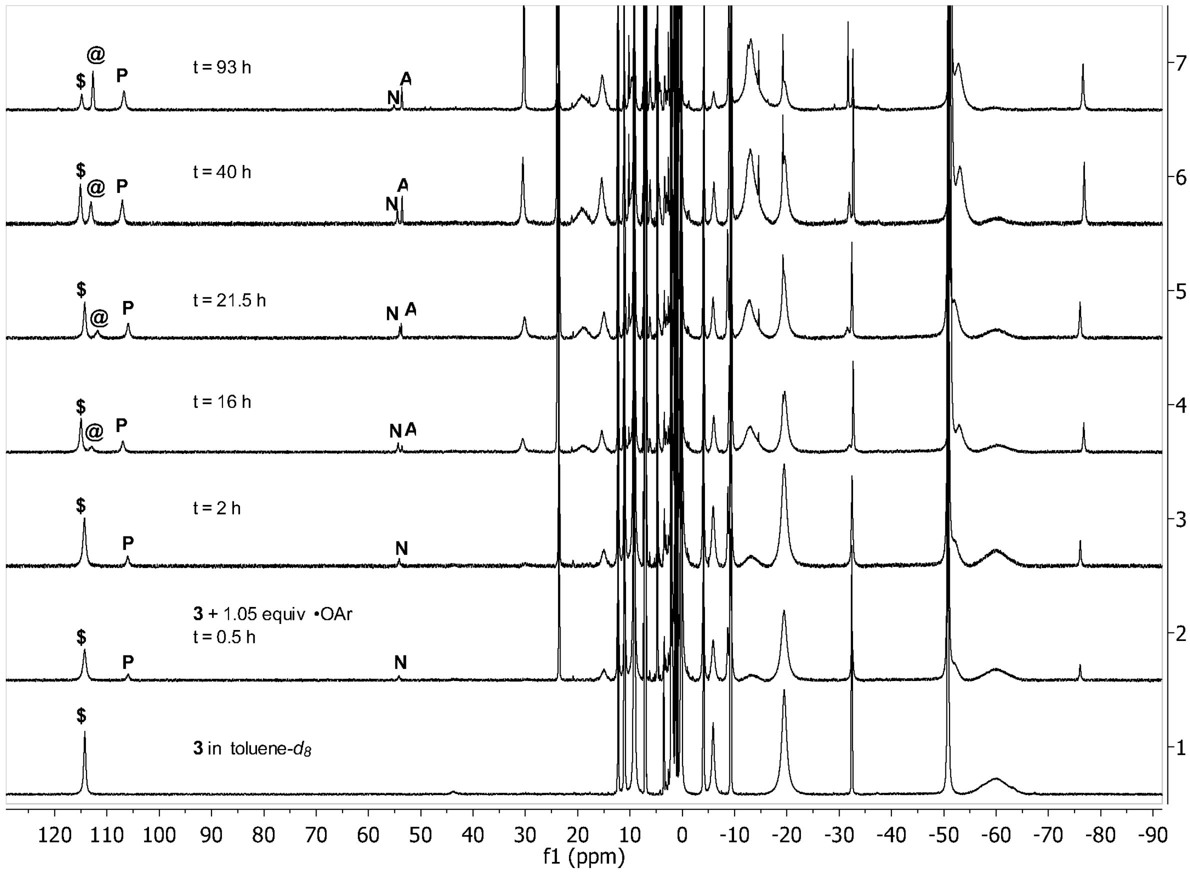

Figure S-30. ^{1}H NMR spectra in toluene-d_8 of the reaction between **3** and 2,4,6-tri-*tert*-butylphenoxyl radical (•OAr; Ar = -$C_6H_3(C(CH_3)_3)_3$) over the course of 93 h with Cp_2Co internal capillary standard (δ -51 ppm). The most downfield peak for each species has been labeled as follows: **P** for **2**, **$** for **3**, **@** for **4**, **A** for **5** and **N** for an unidentified product.

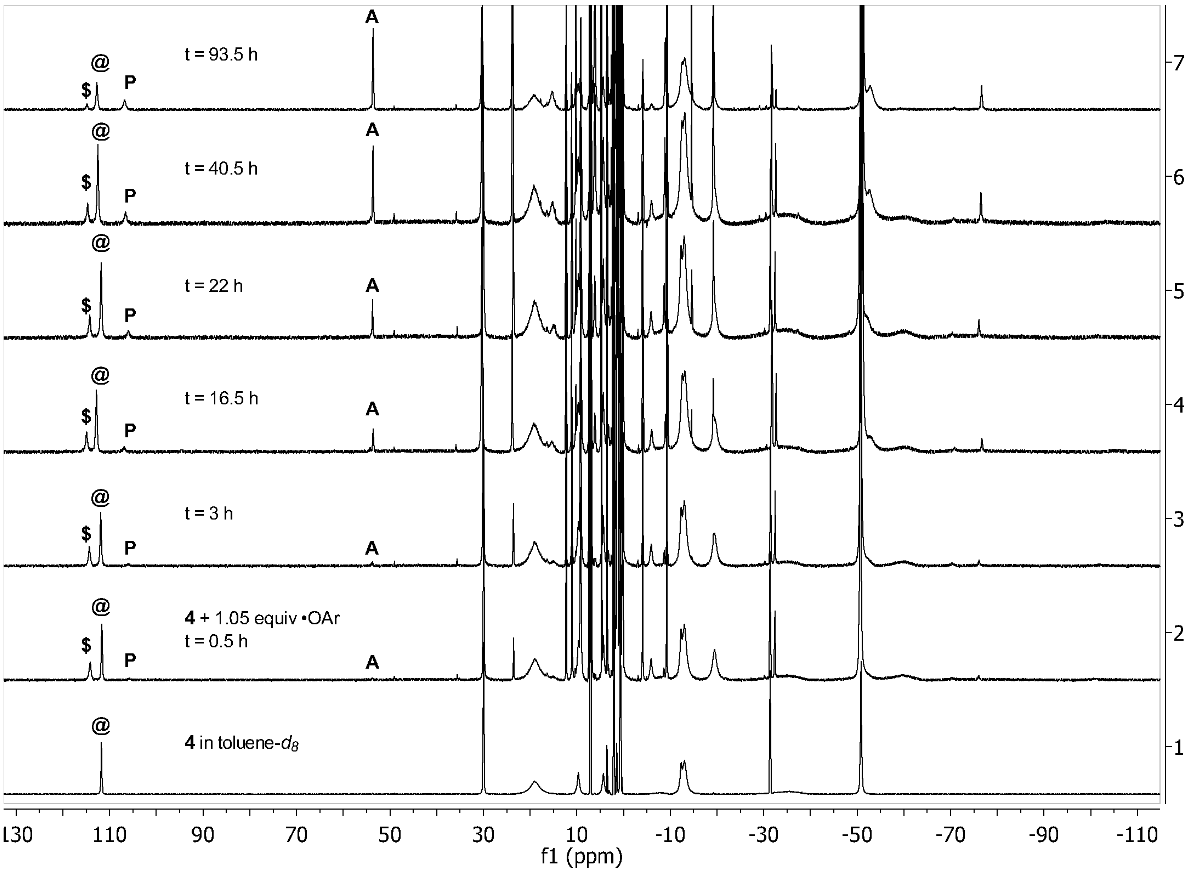

Figure S-31. ^{1}H NMR spectra in toluene-d_8 of the reaction between **4** and 2,4,6-tri-*tert*-butylphenoxyl radical (•OAr; Ar = -$C_6H_3(C(CH_3)_3)_3$) over the course of 93.5 h with Cp_2Co internal capillary standard (δ -51 ppm). The most downfield peak for each species has been labeled as follows: **P** for **2**, **$** for **3**, **@** for **4**, and **A** for **5**.

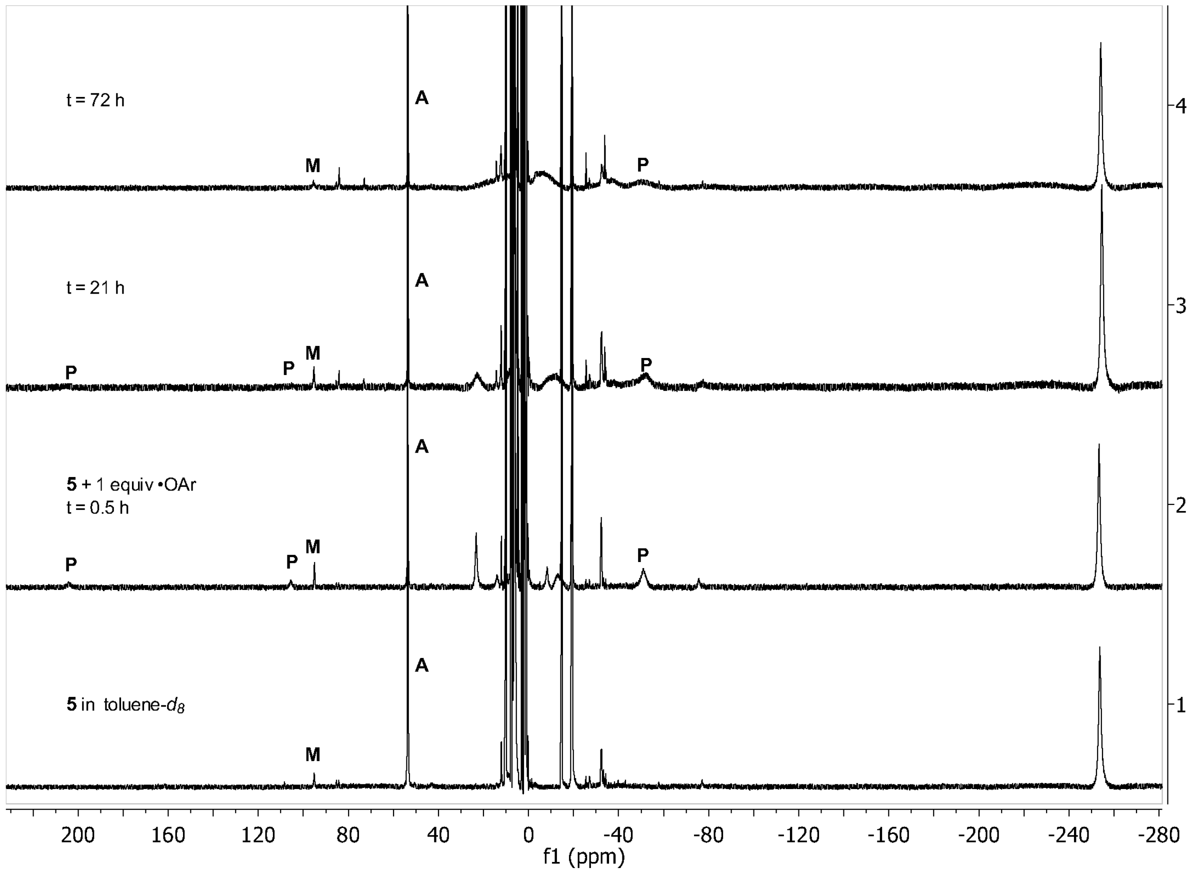

Figure S-32. ^{1}H NMR spectra in toluene-d_8 of the reaction between **5** and 2,4,6-tri-*tert*-butylphenoxyl radical (•OAr; Ar = -$C_6H_3(C(CH_3)_3)_3$) over the course of 72 h with Cp_2Ni internal capillary standard (δ -249 ppm). Diagnostic peaks for each species have been labeled as follows: **P** for **2**, **A** for **5**, and **M** for $[LFe]_2(\mu\text{-Cl})(\mu\text{-NH}_2)$.

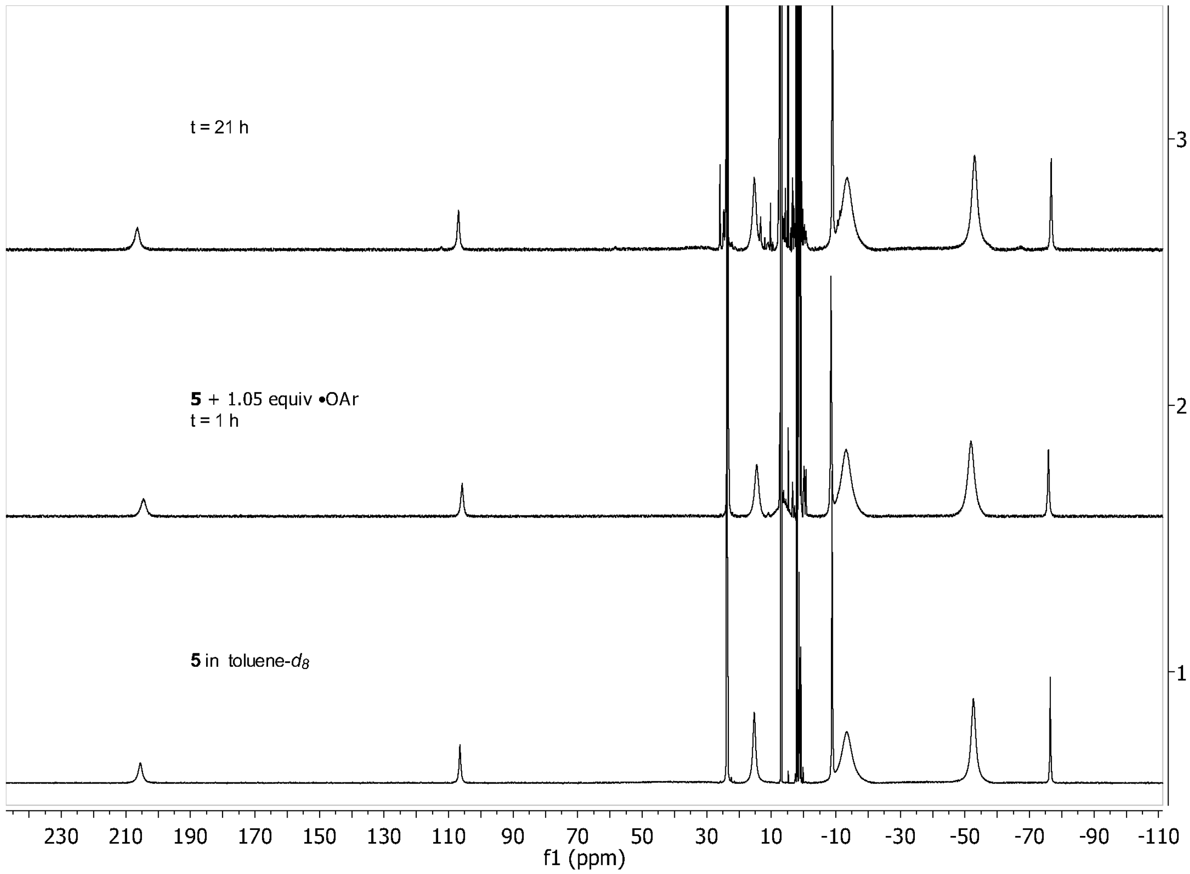

Figure S-33. ^{1}H NMR spectra in toluene-d_8 of the reaction between **2** and 2,4,6-tri-*tert*-butylphenoxyl radical (•OAr; Ar = -$C_6H_3(C(CH_3)_3)_3$) over the course of 21 h.

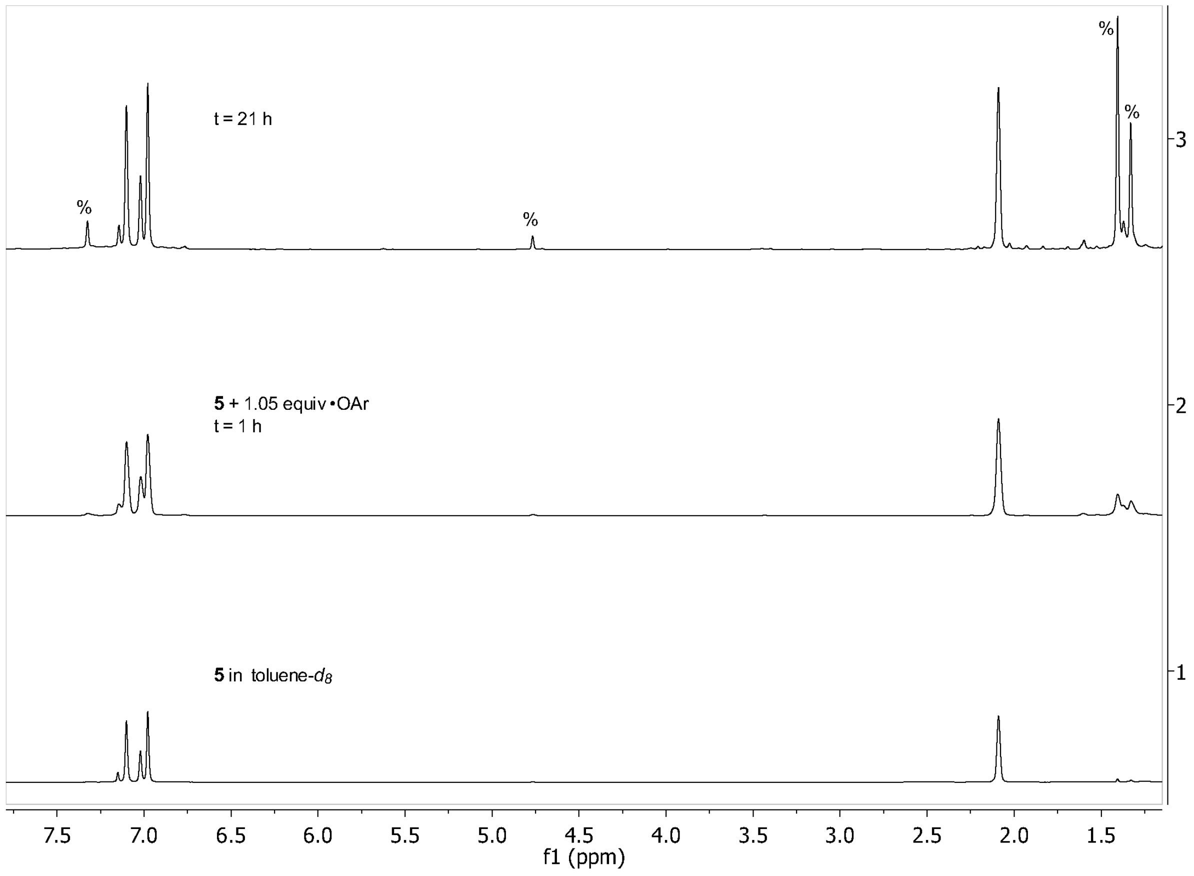

Figure S-34. Diamagnetic region of ^{1}H NMR spectra in toluene-d_8 of the reaction between **2** and 2,4,6-tri-*tert*butylphenoxyl radical (•OAr; Ar = -$C_6H_3(C(CH_3)_3)_3$) over the course of 21 h from S-33. "**%**" mark the resonances attributed to HOAr forming over time in the reaction.

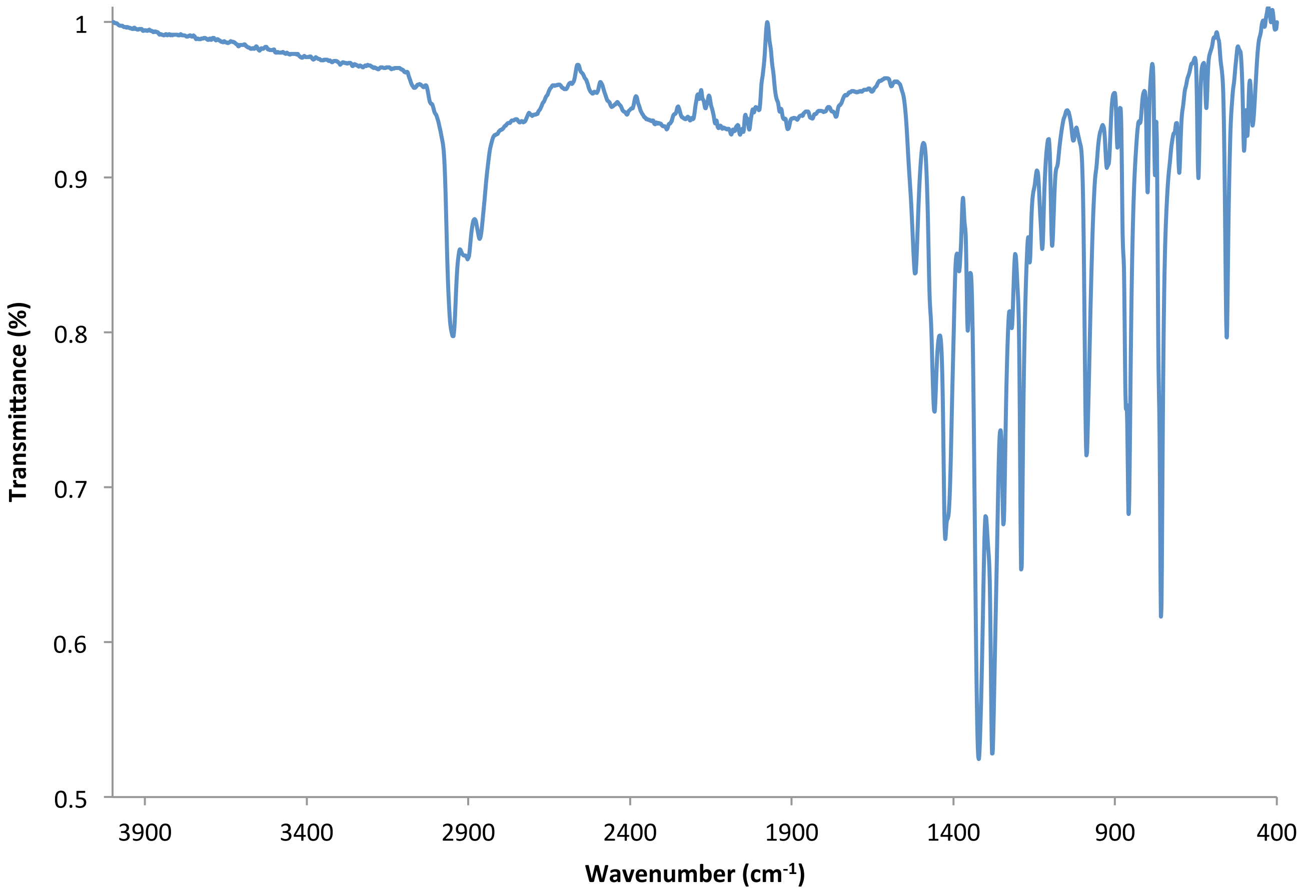

Figure S-35. IR spectrum of LFe($OC_6H_2{}^tBu_3$) (**2**).

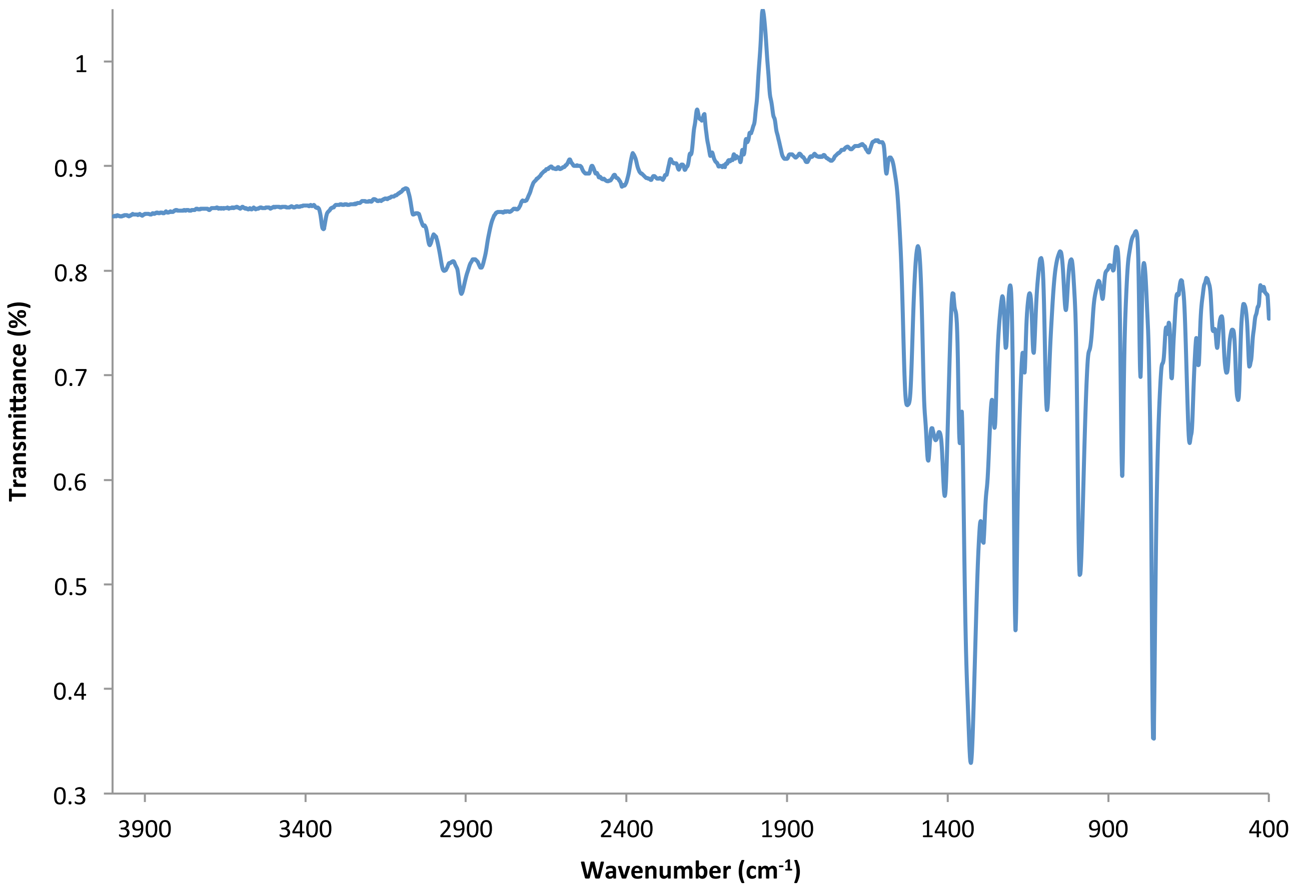

Figure S-36. IR spectrum of $[LFe]_2(\mu_2\text{-NH})(\mu_3\text{-N})[FeL]$ (**3**).

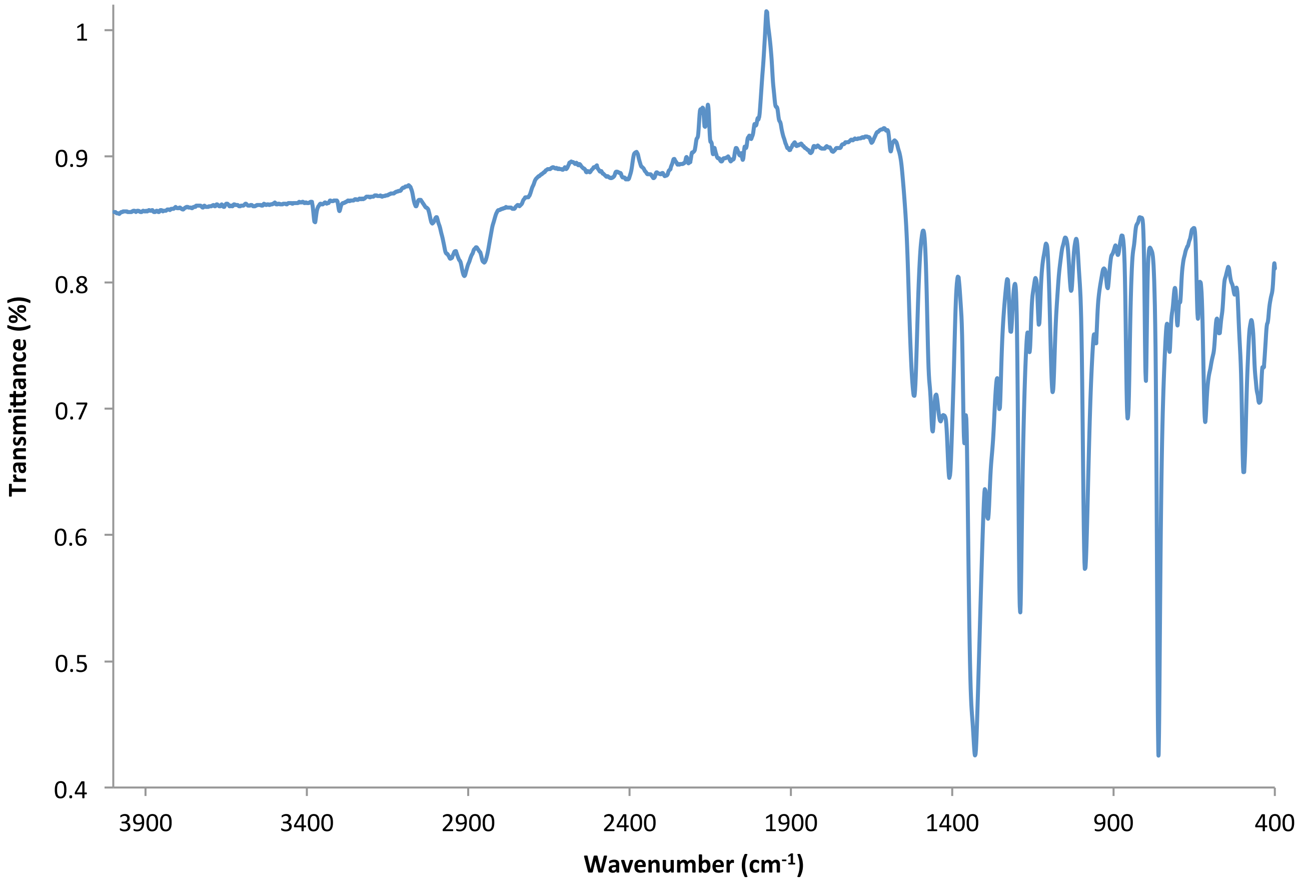

Figure S-37. IR spectrum of [LFe]$_2$(μ_2-NH$_2$)(μ_3-N)[FeL] (**4**).

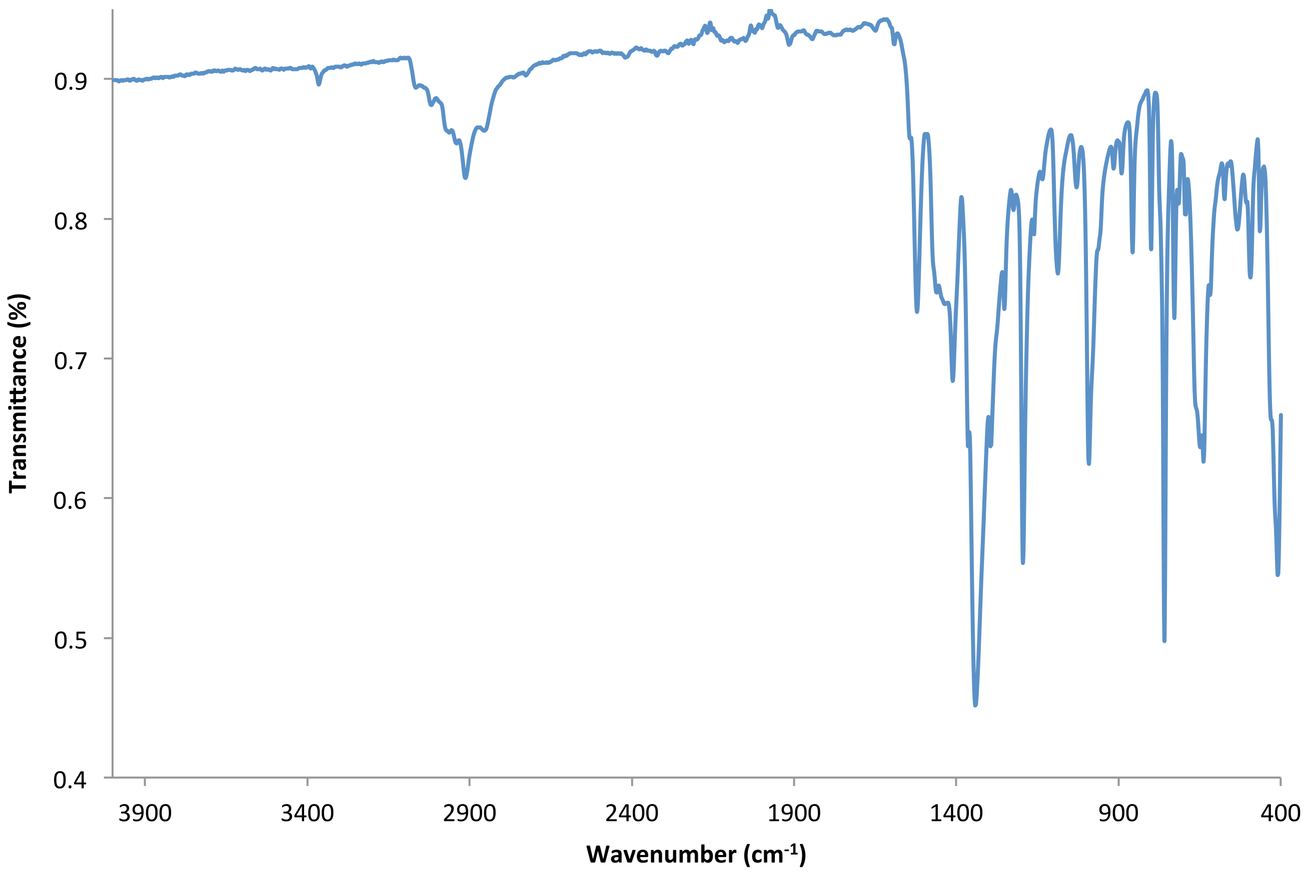

Figure S-38. IR spectrum of $[LFe(\mu\text{-}NH_2)]_2$ (**5**).

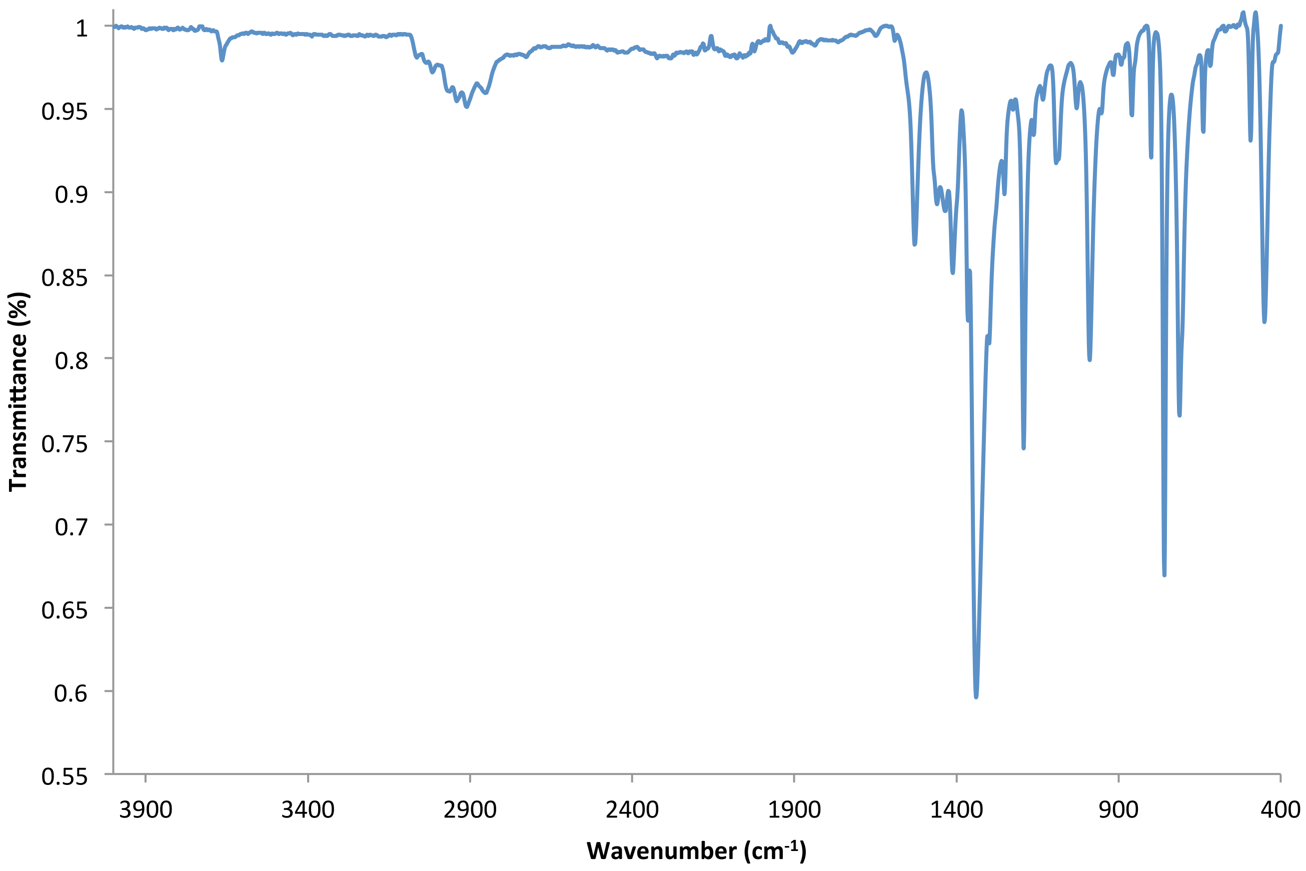

Figure S-39. IR spectrum of $[LFe(\mu\text{-}OH)]_2$ (**6**).

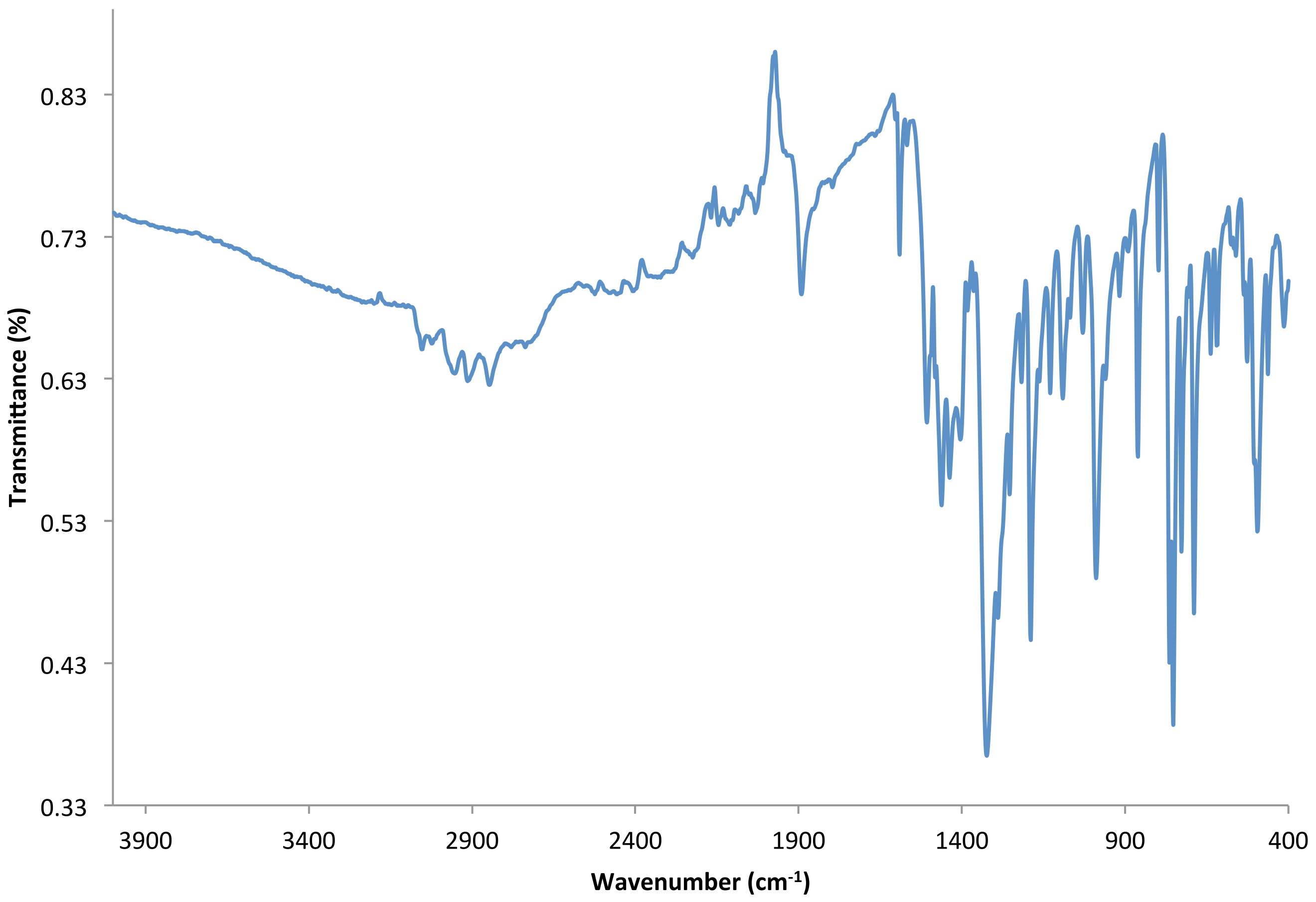

Figure S-40. IR spectrum of [LFe(μ-C≡CPh]$_2$ (**7a**).

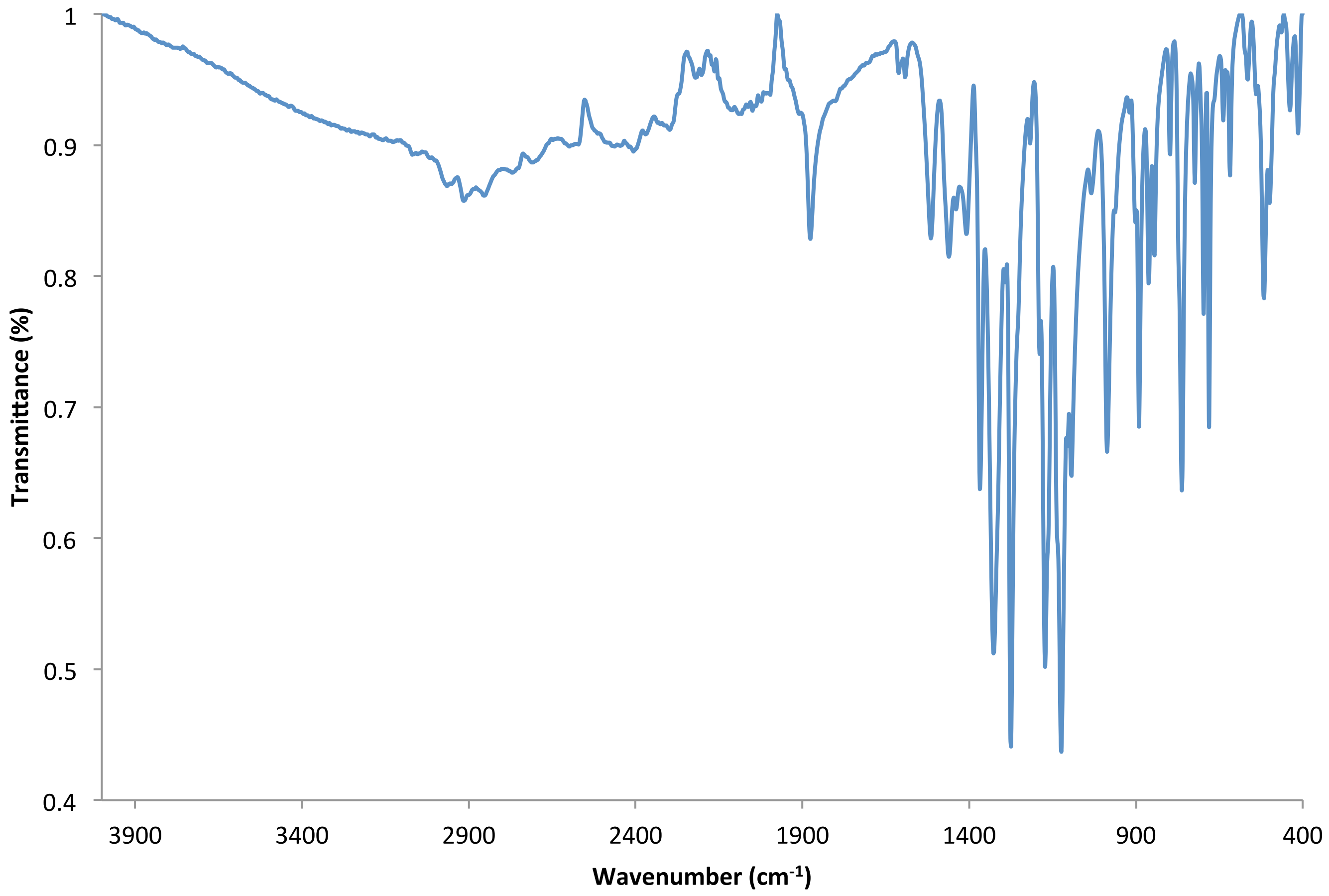

Figure S-41. IR spectrum of the crude solid [LFe{μ-C≡CC$_6$H$_2$(CF$_3$)$_2$}]$_2$ (**7b**).

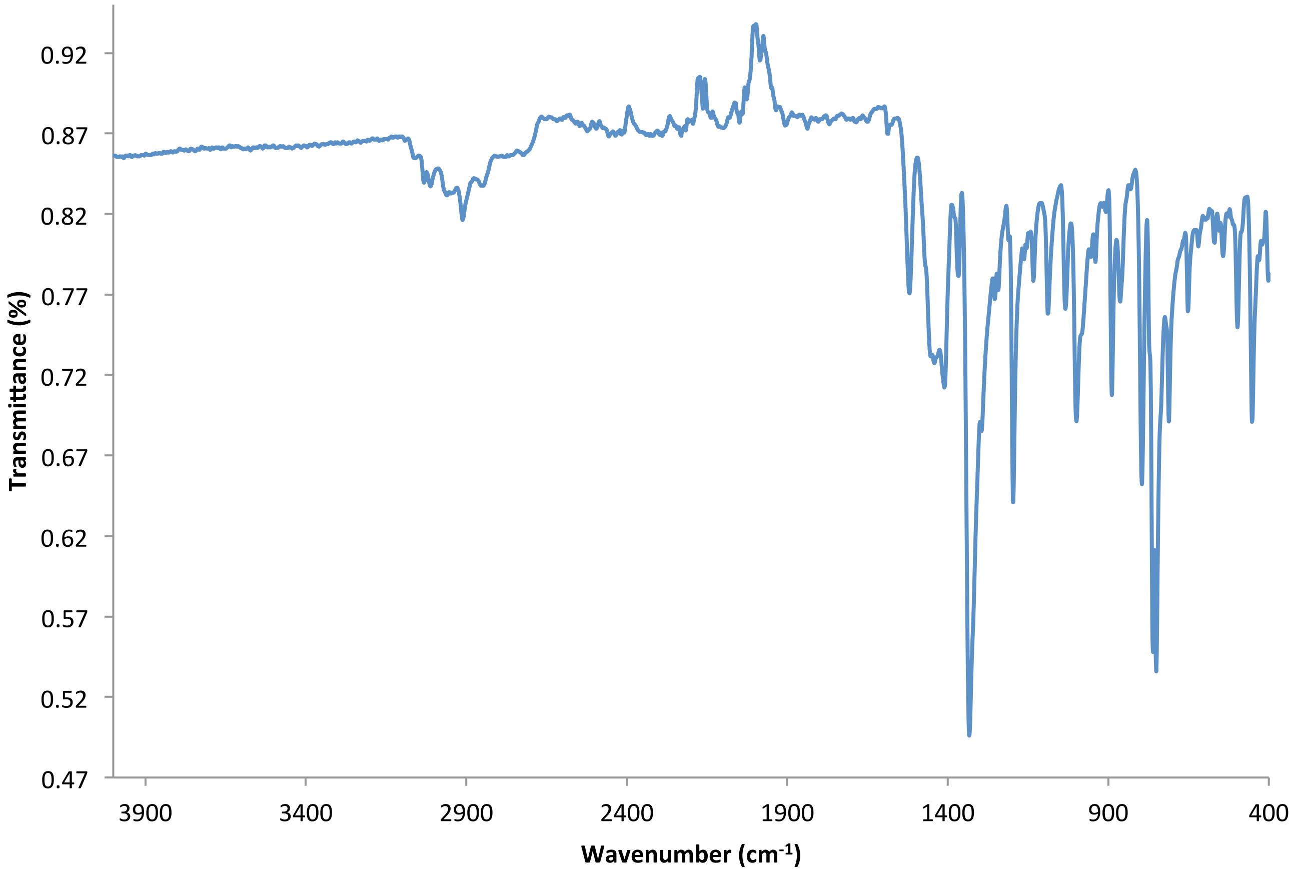

Figure S-42. IR spectrum of LFe(η_5-C_9H_7) (**8**).

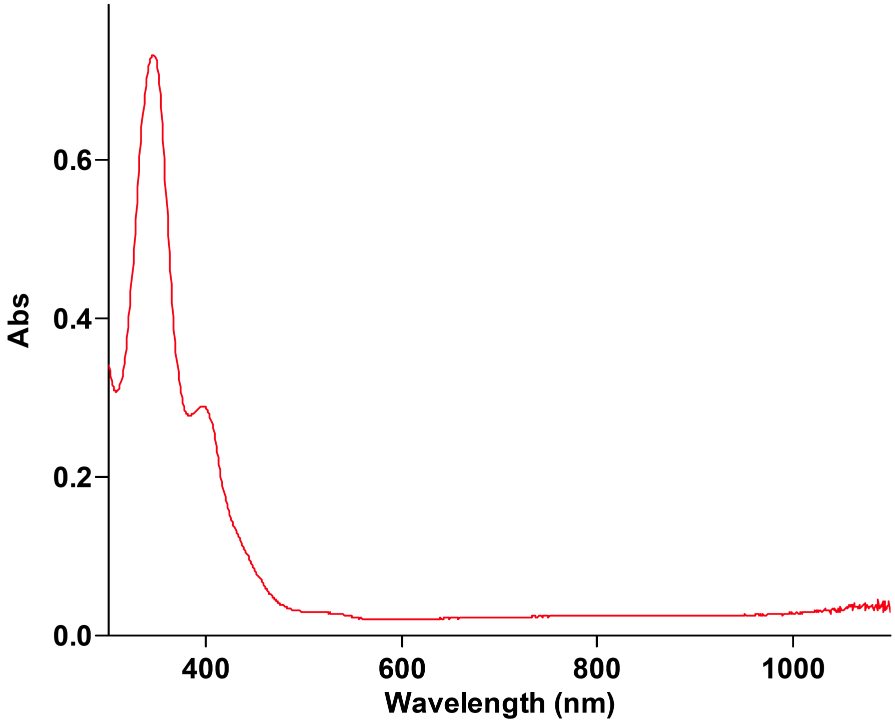

Figure S-43. UV-vis spectrum of $LFe(OC_6H_2{}^tBu_3)$ (**2**) in hexanes, 0.420 mM, path length 0.1 cm.

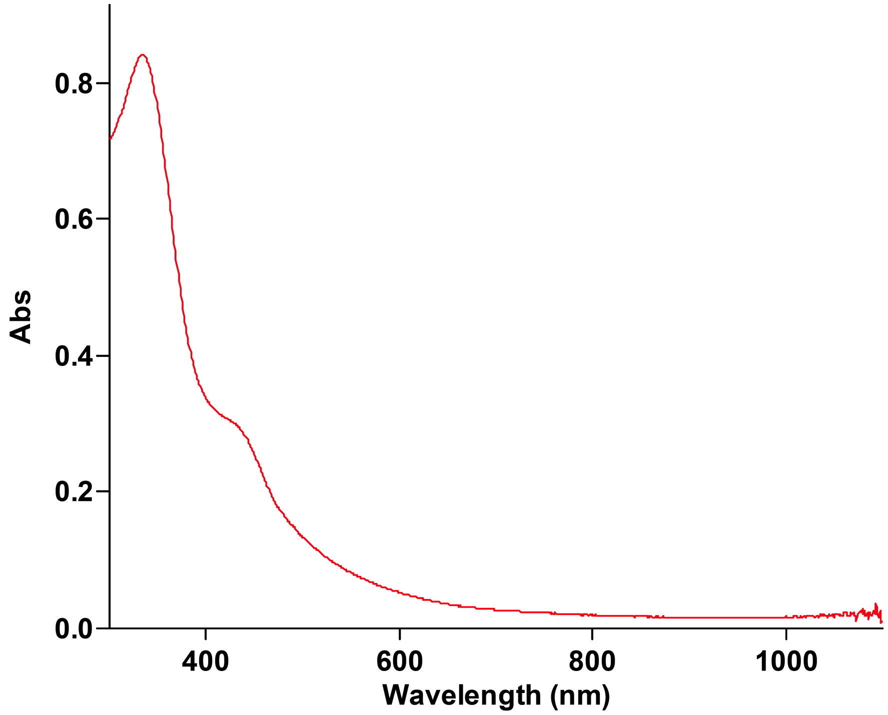

Figure S-44. UV-vis spectrum of $[LFe]_2(\mu_2\text{-}NH)(\mu_3\text{-}N)[FeL]$ (**3**) in toluene, 0.240 mM, path length 0.1 cm.

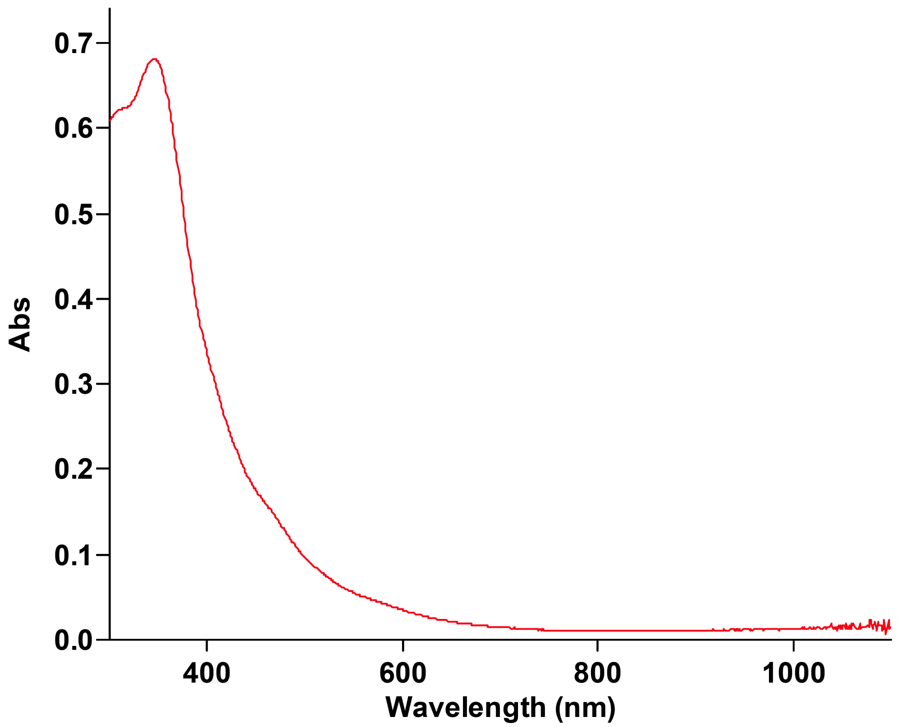

Figure S-45. UV-vis spectrum of $[LFe]_2(\mu_2\text{-}NH_2)(\mu_3\text{-}N)[FeL]$ (**4**) in toluene, 0.206 mM, path length 0.1 cm.

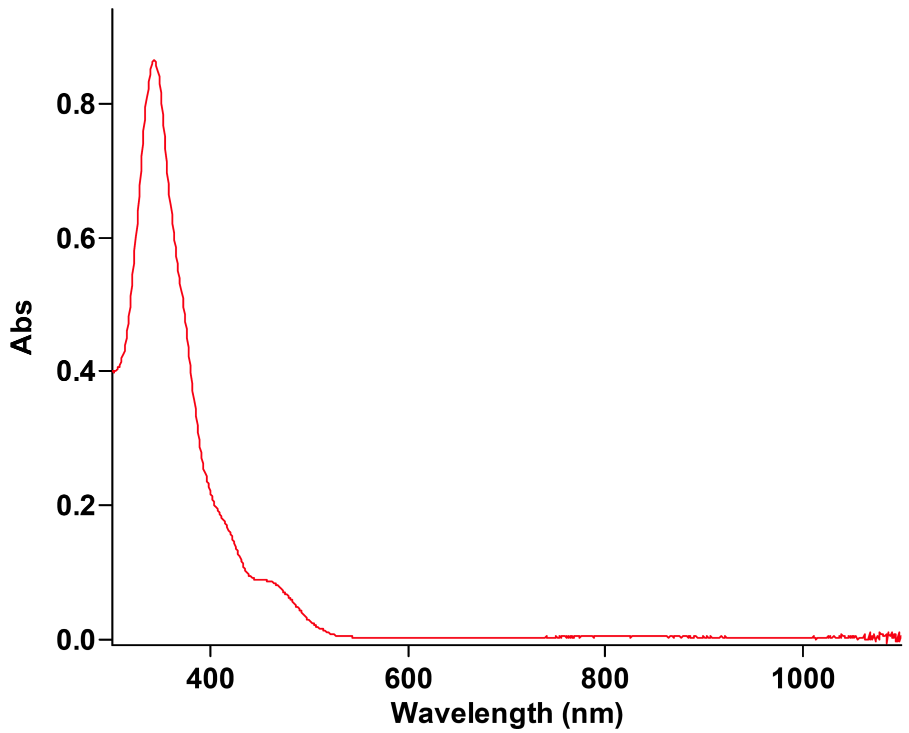

Figure S-46. UV-vis spectrum of $[LFe(\mu\text{-}NH_2)]_2$ (**5**) in toluene, 0.297 mM, path length 0.1 cm.

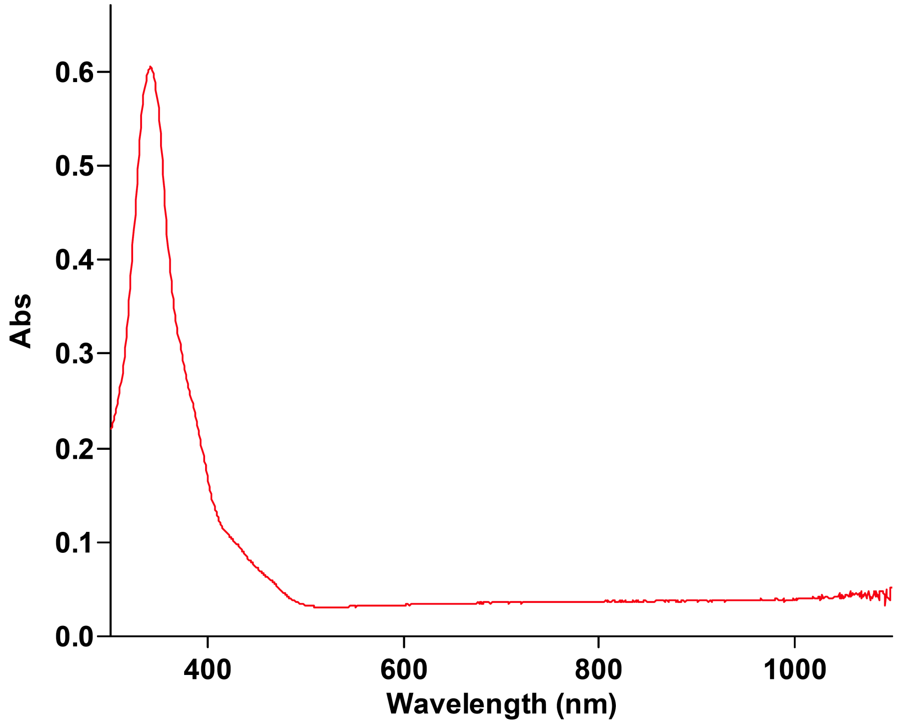

Figure S-47. UV-vis spectrum of [LFe(μ-OH)]$_2$ (**6**) in hexanes, 0.207 mM, path length 0.1 cm.

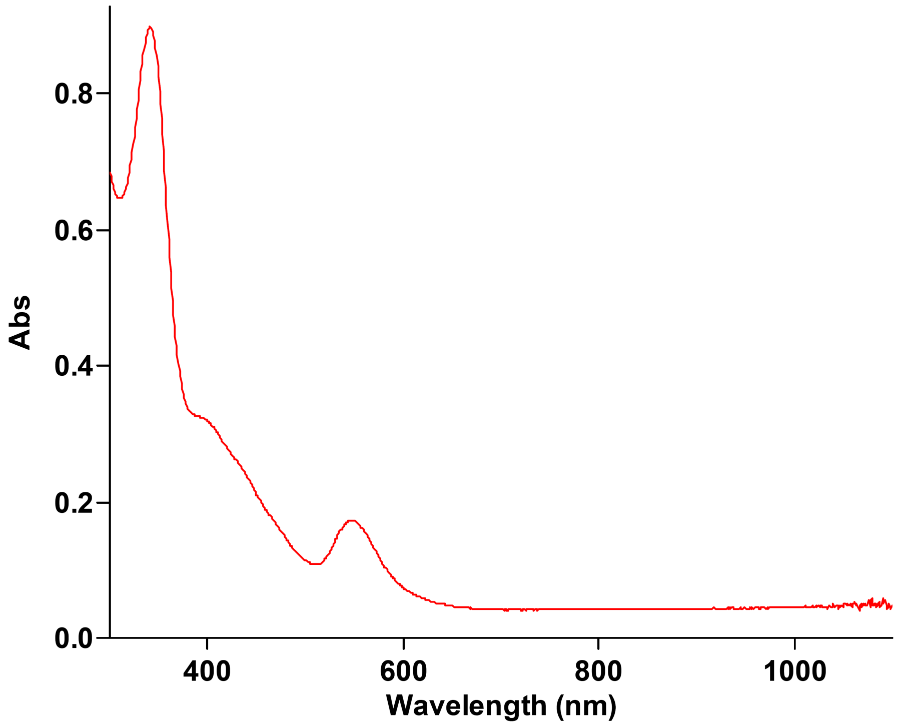

Figure S-48. UV-vis spectrum of [LFe(μ-C≡CPh]$_2$ (**7a**) in toluene, 0.254 mM, path length 0.1 cm.

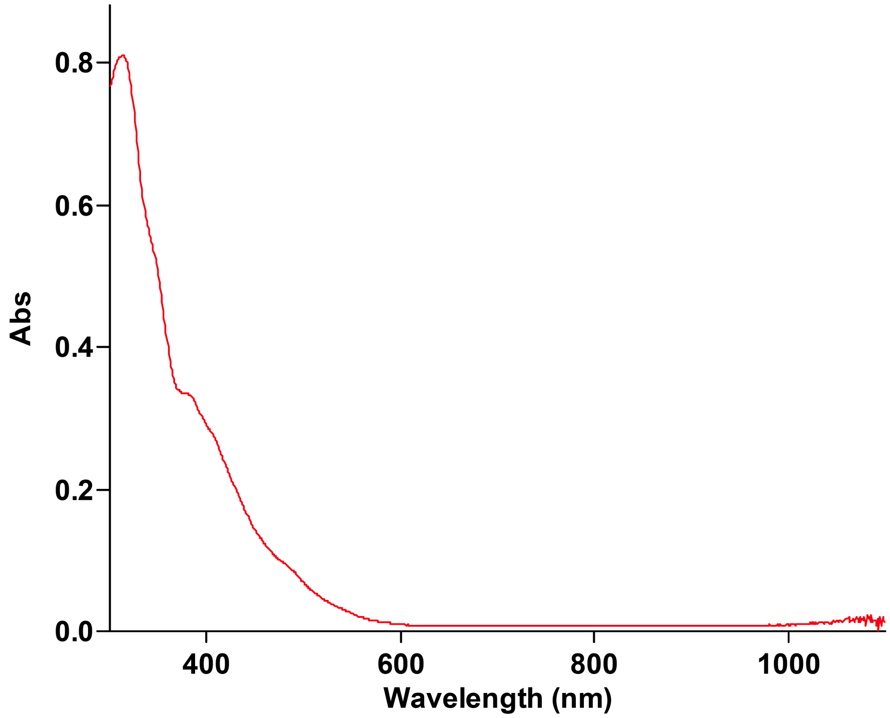

Figure S-49. UV-vis spectrum of LFe(η_5-C_9H_7) (**8**) in hexanes, 0.520 mM, path length 0.1 cm.

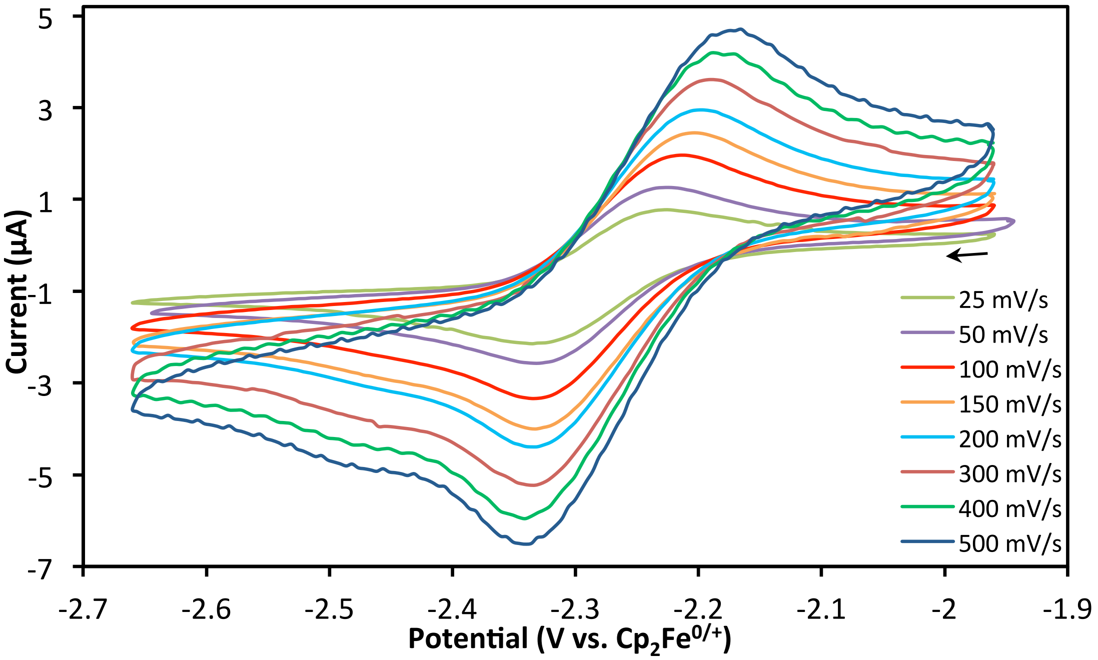

Figure S-50. Cyclic voltammograms of $[LFe]_2(\mu_2\text{-NH})(\mu_3\text{-N})[FeL]$ (**3**) in THF, 1 mM, $E_{1/2}$ = -2.28 V versus $Cp_2Fe^{0/+}$.

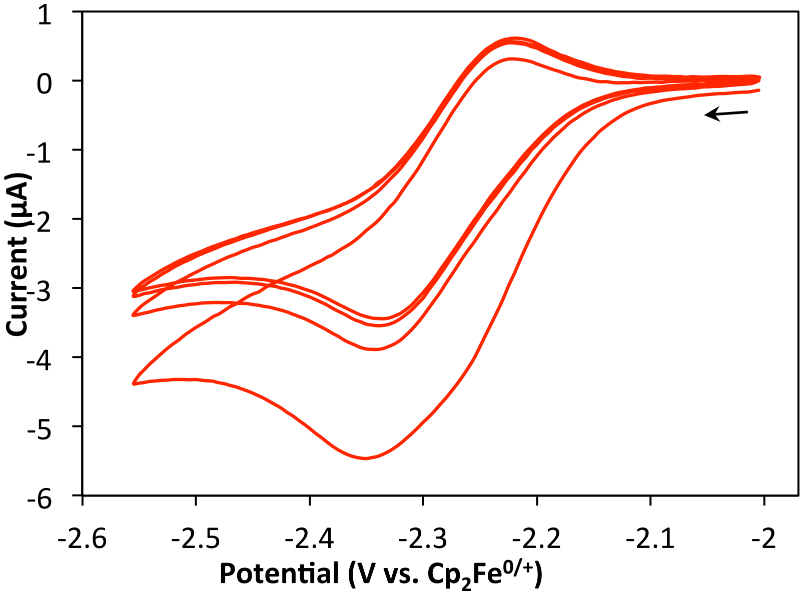

Figure S-51. Cyclic voltammogram of **3** (1 mM) in THF with indene (26 mM). Scan rate = 50 mV/s, 4 scans.

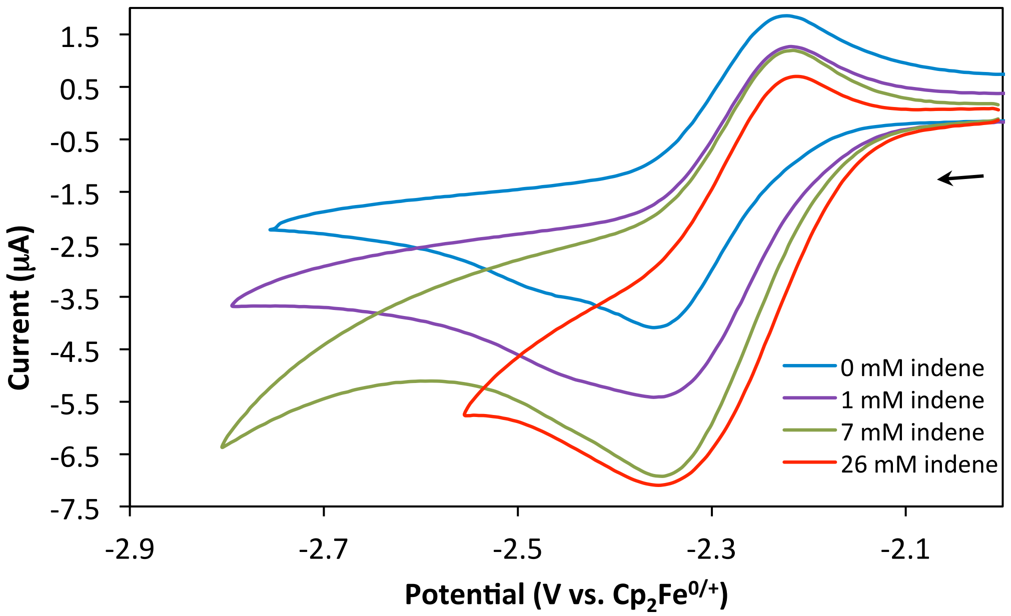

Figure S-52. Cyclic voltammograms of **3** (1 mM) in THF with indene (0 to 26 mM). Scan rate = 100 mV/s.

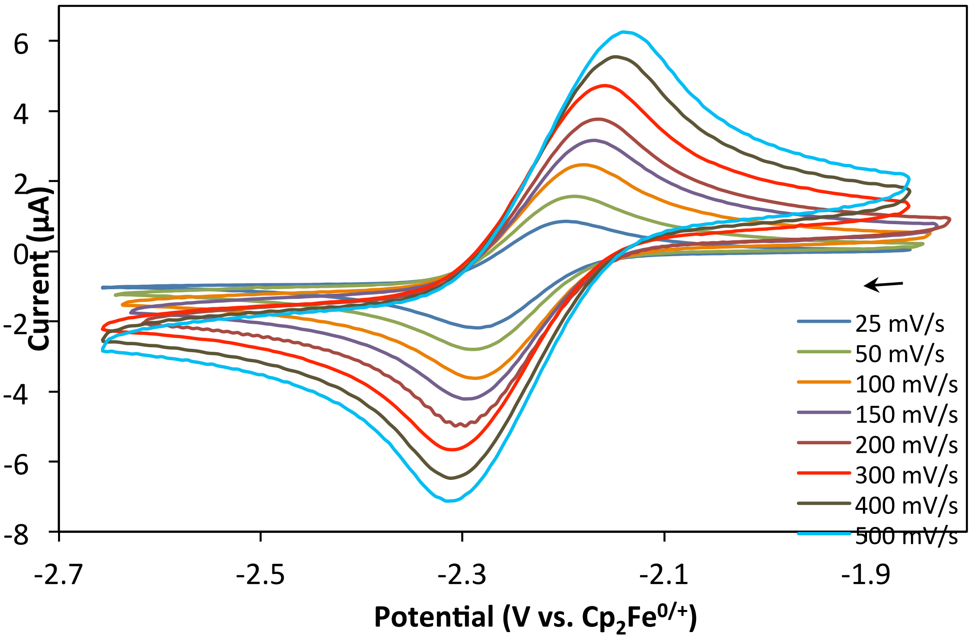

Figure S-53. Cyclic voltammograms of $[LFe]_2(\mu_2\text{-}NH_2)(\mu_3\text{-}N)[FeL]$ (**4**) in THF, 1 mM, $E_{1/2}$ = -2.27 V versus $Cp_2Fe^{0/+}$.

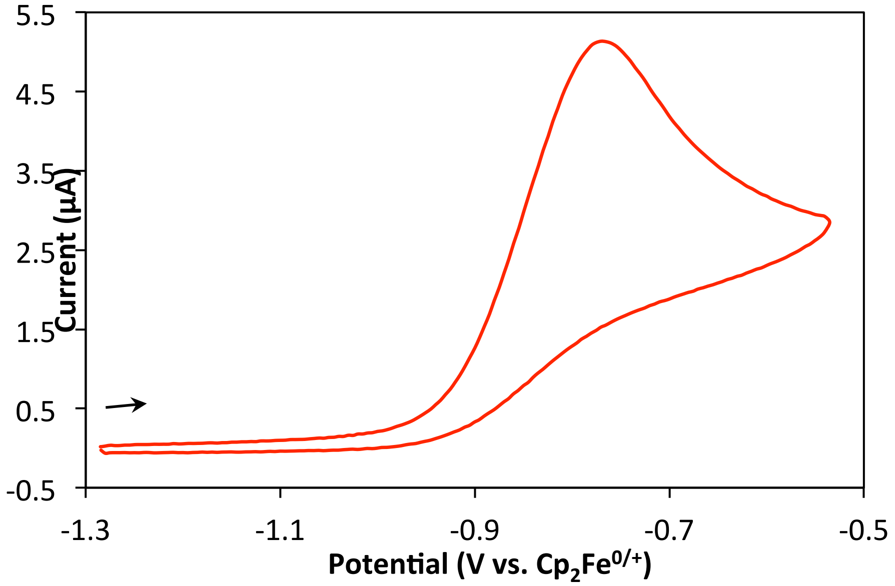

Figure S-54. Cyclic voltammogram of $[LFe]_2(\mu_2\text{-}NH_2)(\mu_3\text{-}N)[FeL]$ (**4**) in THF, 1 mM, E_{ox} = -0.77 V versus $Cp_2Fe^{0/+}$. Scan rate = 200 mV/s.

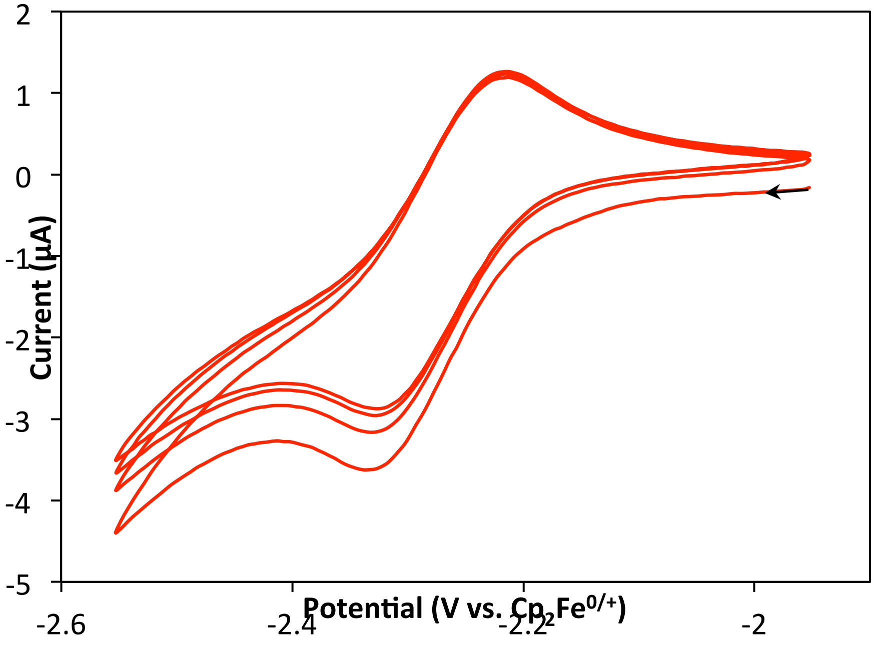

Figure S-55. Cyclic voltammogram of **4** (1 mM) in THF with indene (26 mM). Scan rate = 50 mV/s, 4 scans.

X-ray Crystallography

Low-temperature diffraction data (ω-scans) were collected on a varaity of instrumnets. Compound **3** was collected on a Rigaku MicroMax-007HF diffractometer coupled to a Saturn994+ CCD detector with Cu Kα (λ = 1.54178 Å); compounds **4**, **5**, and **8** were collected on a Rigaku R-AXIS RAPID diffractometer coupled to an R-AXIS RAPID imaging plate; compounds **2** and **7a** were collected on a Rigaku SCX Mini diffractometer coupled to a Rigaku Mercury275R CCD; compound **6** was collected on a Bruker SMART APEX II CCD Platform diffractometer. The diffraction images for **2**, **3**, **4**, **5**, **7a**, and **8** were processed and scaled using the Rigaku CrystalClear software package.[13] These structures were solved with SHELXT and refined against F^2 on all data by full-matrix least squares with SHELXL.[14] The diffraction images for **6** were processed and scaled using the Bruker APEX software package.[15] The structure was solved using SIR97[16] and refined using SHELXL-97.[14] All non-hydrogen atoms were refined anisotropically. Unless stated otherwise, hydrogen atoms were included in the model at geometrically calculated positions and refined using a riding model. The isotropic displacement parameters of all hydrogen atoms were fixed to 1.2 times the U value of the atoms to which they are linked (1.5 times for methyl groups). Full details of the X-ray structure determination are in the CIF included as Supporting Information.

Refinement Details

Compound 3. The hexane solvent is disordered with respect to the crystallographic screw axis. The difference map suggests electron density that could be modeled as a heptane. However, under closer inspection the terminal methyl groups where found to each represent a half of one carbon atom, which suggests that the 5 core carbon atoms are fully occupied, while the terminal carbon atom has two disordered positions. The hydrogen atoms on the hexane molecule were geometrically generated and refined with a riding model to reflect this disorder. No special

restraints were needed for the solvent model. The only constraints used reflect the disordered position of the terminal methyl with respect to the screw axis.

One of the flanking xylene groups is disordered. The atoms are modeled over two positions and involve {C19A, C29A, C39A, C49A, C59A, C69A, C79A, C89A} and {C19B, C29B, C39B, C49B, C59B, C69B, C79B, C89B}. The thermal ellipsoids were expected to have similar directions. The "A" and "B" components of the disorder were each restrained with rigid bond restraints to reflect this fact. The site occupancies of the "A" and "B" components were freely refined and covered at the values of 0.43(3) and 0.57(3), respectively. The atom C69A and C89A were constrained to have the same thermal parameter due to the near superposition of C89A with C89B.

*Compound **4***. Hydrogen atoms H2A and H2B were found in the difference map and freely refined.

*Compound **5***. The hydrogen atoms on N1 were easily found in the difference map and are freely refined. The hydrogen atoms on N2 were more difficult to locate. The electron density suggest that the hydrogen atoms are very close proximity to the heteroatom and in a non-ideal geometry. Consequently, the distances between each of the hydrogen and nitrogen atoms were restrained to to be similar. The distance between the H2A and H2B was also restrained to be nearly the same as H1A-H1B.

*Compound **6***. The hydrogen atoms were found from the difference map, disordered over two positions at each oxygen atom (70:30). The positional and isotropic displacement parameters for those in the major occupancy sites were refined independently from those of the oxygen atoms. The minor occupancy hydrogen atom positions and their isotropic displacement parameters were refined relative to those of the oxygen atoms.

*Compound **8***. Hydrogen atoms H34, H44, H3B, and H4B were found in the difference map and semi-freely refined on the indinyl group.

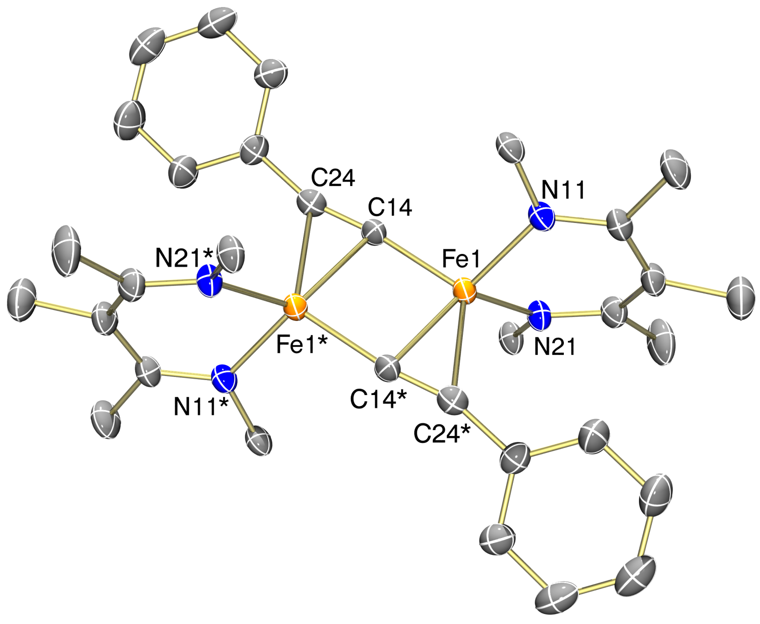

Figure S-56. Thermal-ellipsoid plot of the molecular structure of $[LFe(\mu\text{-}C{\equiv}CPh)]_2$ (**7a**) using 50% thermal ellipsoids. The 2,6-dimethylphenyl groups and all hydrogen atoms are omitted for clarity.

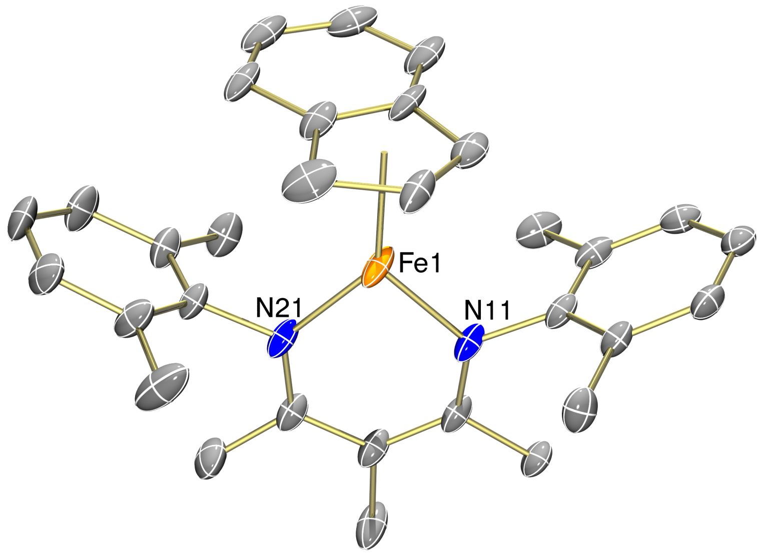

Figure S-57. Thermal-ellipsoid plot of the molecular structure of $LFe(\eta_5\text{-}C_9H_7)$ (**8**) using 50% thermal ellipsoids. All hydrogen atoms are omitted for clarity.

Table S-3. Details of X-ray crystal structures **2**, **3**, and **4**.

Compound	$LFe(OC_6H_2{}^tBu_3)$ (**2**)	$[LFe]_2(\mu_2\text{-}NH)(\mu_3\text{-}N)[FeL] \cdot 0.5(C_6H_{14})$ (**3**)	$[LFe]_2(\mu_2\text{-}NH_2)(\mu_3\text{-}N)[FeL] \cdot C_5H_{12}$ (**4**)
Empirical formula	$C_{40}H_{56}N_2OFe$	$C_{69}H_{89}N_8Fe_3$	$C_{71}H_{95}N_8Fe_3$
FW	636.71	1198.03	1228.09
Crystal system	Monoclinic	Monoclinic	Monoclinic
Space group	$P\,2_1/n$	$I\,2/a$	$I\,2/a$
Wavelength	0.71073	1.54178	0.71073
a (Å)	10.9741(4)	25.7232(18)	26.1506(18)
b (Å)	21.7440(8)	12.6083(4)	12.4838(2)
c (Å)	15.2450(11)	38.521(4)	39.818(4)
α (deg)	90	90	90
β (deg)	96.681(7)	92.547(9)	90.725(9)
γ (deg)	90	90	90
V (Å^3)	3613.1(3)	12481.1(16)	12997.9(16)
Z	4	8	8
ρ (g/cm^3)	1.171	1.275	1.255
μ (mm^{-1})	0.449	5.874	0.708
$R1$, w$R2$ (I > 2σ(I))	0.0697, 0.1171	0.0756, 0.1807	0.0483, 0.1121
$R1$, $wR2$ (all data)	0.1016, 0.1268	0.1130, 0.2180	0.0675, 0.1216
GOF	1.151	0.975	1.044

Table S-4. Details of X-ray crystal structures **5**, **6**, **7a**, and **8**.

Compound	$[LFe(\mu\text{-}NH_2)]_2 \cdot C_7H_8$ (**5**)	$[LFe(\mu\text{-}OH)]_2 \cdot C_4H_{10}O$ (**6**)	$[LFe(\mu\text{-}C{\equiv}CPh]_2$ (**7a**)	$LFe(\eta_5\text{-}C_9H_7)$ (**8**)
Empirical formula	$C_{51}H_{66}N_6Fe_2$	$C_{48}H_{66}N_4O_3Fe_2$	$C_{60}H_{64}N_4Fe_2$	$C_{31}H_{34}N_2Fe$
FW	874.79	858.75	952.85	490.45
Crystal system	Triclinic	Triclinic	Triclinic	Monoclinic
Space group	$P\bar{1}$	P-1	$P\bar{1}$	$P\ 2_1/c$
Wavelength	0.71073	0.71073	0.71073	0.71073
a (Å)	11.6617(4)	12.7872(8)	10.8292(17)	8.1716(2)
b (Å)	12.8275(4)	12.8768(8)	12.1016(19)	18.2611(3)
c (Å)	17.826(3)	14.4731(9)	12.4355(19)	17.0223(12)
α (deg)	110.292(7)	77.782(1)	104.019(7)	90
β (deg)	99.483(8)	84.648(1)	113.332(8)	99.181(7)
γ (deg)	103.940(7)	82.055(1)	108.094(8)	90
V (Å^3)	2335.4(4)	2301.7(2)	1291.9(4)	2507.6(2)
Z	2	2	1	4
ρ (g/cm^3)	1.244	1.239	1.225	1.299
μ (mm^{-1})	0.662	0.673	0.603	0.623
$R1$, w$R2$ (I > 2σ(I))	0.0349, 0.0883	0.0534, 0.1037	0.0734, 0.1680	0.0376, 0.0923
$R1$, $wR2$ (all data)	0.0425, 0.0924	0.1124, 0.1250	0.0940, 0.1794	0.0434, 0.0956
GOF	1.050	1.009	1.171	1.089

References

[1] K. Michiue and R. F. Jordan, *J. Mol. Catal. A: Chem.*, 2008, **282**, 107-116.

[2] V. W. Manner, T. F. Markle, J. H. Freudenthal, J. P. Roth, J. M. Mayer, *Chem. Commun.,* 2008, 256-258.

[3] N. A. Giffin, M. Makramalla, A. D. Hendsbee, K. N. Robertson, C. Sherren, C. C. Pye, J. D. Masuda and J. A. C. Clyburne, *Org. Biomol. Chem.*, 2011, **9**, 3672-3680.

[4] K. Grubel, W. W. Brennessel, B. Q. Mercado and P. L. Holland, *J. Am. Chem. Soc.*, 2014, **136**, 16807-16816.

[5] M. M. Rodriguez, E. Bill, W. W. Brennessel and P. L. Holland, *Science*, 2011, **334**, 780-783.

[6] E. M. Schubert, *J. Chem. Educ.*, 1992, **69**, 62.

[7] A. L. Chaney and E. P. Marbach, *Clin. Chem.*, 1962, **8**, 130-132.

[8] G. W. Watt and J. D. Chrisp, *Anal. Chem.*, 1952, **24**, 2006-2008.

[9] J. Braunschweig, J. Bosch, K. Heister, C. Kuebeck and R. U. Meckenstock, *J. Microbiol. Meth.*, 2012, **89**, 41-48.

[10] K. P. Chiang, P. M. Barrett, F. Ding, J. M. Smith, S. Kingsley, W. W. Brennessel, M. M. Clark, R. J. Lachicotte and P. L. Holland, *Inorg. Chem.*, 2009, **48**, 5106-5116.

[11] (a) X. Wang, J. Zhang, L. Wang and L. Deng, *Organometallics*, 2015, **34**, 2775-2782. (b) N. A. Arnet, T. R. Dugan, F. S. Menges, B. Q. Mercado, W. W. Brennessel, E. Bill, M. A. Johnson and P. L. Holland, *J. Am. Chem. Soc.*, 2015, **137**, 13220-13223.

[12] E. Münck, in *Physical Methods in Bioinorganic Chemistry*, ed. L. Que, University Science Books, New York, 2000, pp. 287-319.

[13] CrystalClear and CrystalStructure; Rigaku/MSC: The Woodlands, TX, 2005.

[14] G. Sheldrick, *Acta Cryst.*, 2008, **A64**, 112-122.

[15] (a) *APEX2*, version 2011.4-1; Bruker AXS: Madison, WI, 2011. (b) *SAINT*, version 7.68A; Bruker AXS: Madison, WI, 2009.

[16] A. Altomare, M. C. Burla, M. Camalli, G. L. Cascarano, C. Giacovazzo, A. Guagliardi, A. G. G. Moliterni, G. Polidori, R. Spagna, *SIR97: A new program for solving and refining crystal structures*; Istituto di Cristallografia, CNR: Bari, Italy, 1999.
